# Abstracts of scientific papers and sessions, CPA Congress 2013

**DOI:** 10.3138/physio.65.supp

**Published:** 2013-05

**Purpose & Objectives / Intention & objectifs:** To systematically review the evidence examining the physical effects of patient isolation due to multidrug-resistant organisms (MROs) and the interventions implemented to address these effects. Increasing numbers of inpatients are isolated in limited spaces due to infection control precautions. It is important to know the effect that isolation has on patients’ health when physical morbidity is a well-documented, longterm result of immobility.

**Relevance/Pertinence:** Physiotherapists are the primary practitioners prescribing and monitoring inpatient mobility and are thus key to the maintenance of patients’ physical capacity and independence.

**Materials & Methods/Matériel & méthods:** Medline, CINAHL, and EMBASE databases were searched from 1955 to December 2011. Papers in English, incorporating evaluation of adverse effects of inpatient isolation for infection control purposes or prolonged inactivity and those reporting the effects of exercise in various inpatient cohorts were included.

**Analysis/Analyse:** The strength of evidence was evaluated using a critical appraisal tool adapted from the Published Guides and Recommended Questions for Appraising Reports of Medical Interventions (Reed, 2005). Study quality was scored on study purpose, rationale (Yes:2, Improvement required:1, or No:0); and objectives, study design, intervention, evaluation, results (Excellent: 4, Good:3, Moderate:2, Poor:1, or No evidence:0).

**Results/Résultats:** The search strategy yielded 58 papers with rankings of 14 (moderate quality) or above. Although the research suggests that immobility is associated with inpatient musculoskeletal, neuromuscular, cardiovascular and functional adverse effects, there are no studies investigating patients on isolation precautions. **Conclusions:** No conclusions regarding the physical effects of patient isolation due to MROs or their treatment can be made due to the absence of investigations into this population.

**Keywords/Mots clés:** Patient isolation, contact precautions, contact isolation, multidrug-resistant organisms, inpatients, confined space, bed rest, immobilization, cardiovascular abnormalities, aerobic capacity, deconditioning, bone density, contractures, skeletal muscle, musculoskeletal abnormalities, venous thrombosis, exercise, aerobic exercise, hospitalized patients, inpatients, hospitalization, intensive care unit, and rehabilitation.

