# Supplementary material for: Abstracts of scientific papers and sessions, CPA Congress 2013
Source: Physiother Can. 2013 May;65(Suppl 1):1–165. doi: 10.3138/physio.65.supp (PMC4098091; doi:10.3138/physio.65.supp)
Supplement: Related texts

# Abstract: A005 – No Differences in Outcomes in Subjects with Low Back Pain who met the Clinical Prediction Rule for Lumbar Spine Manipulation when Non-thrust Manipulation was used as the Comparator

**Published:** 2013-05

**Authors:** C Cook, K Learman

**Affiliations:** * Walsh University, North Canton, Ohio, USA; † Youngstown State, Youngstown, Ohio, US

**Purpose & Objectives / Intention & objectifs:** The purpose of the study was to compare thrust manipulation to non-thrust manipulation in patients who met the clinical prediction rule (CPR) for spinal manipulation.

**Relevance/Pertinence:** The CPR for spinal manipulation has been advocated for use in clinical practice guidelines despite that the majority of physiotherapists do not use thrust manipulation.

**Materials & Methods/Matériel & méthods:** The secondary data analysis (N=71) was extracted from a randomized controlled trial, which compared thrust manipulation to non-thrust manipulation. Whether or not subjects met the lumbar spine CPR was assessed at baseline. Seventeen highly trained manual therapists with a strong background in thrust and non-thrust manipulation provided the techniques. Manipulative techniques were allowed to be tailored to the patients’ comparable findings.

**Analysis/Analyse:** Comparative baseline values were calculated for both groups. Oswestry Disability Index change scores, Numeric Pain Rating Scale for pain change scores, total visits, total days in care, and self- report of rate of recovery were analyzed for each group using standard t-tests with adjustments for multiple measures.

**Results/Résultats:** There were no differences in baseline between groups. There were no significant differences between thrust and non-thrust manipulation in any of the outcomes measures. **Conclusions:** The original CPR studies that validated the tool investigated thrust manipulation versus a comparator of general low back exercises. When a comparator of non-thrust manipulation was used, and when clinicians were allowed to use a method that was focused to the comparable findings of the patient then outcomes are similar between thrust and non-thrust manipulation.

**Keywords/Mots clés:** Low back pain, Non-thrust Manipulation, Thrust Manipulation, Clinical prediction rule

# Abstract: A012 – Quality Improvement and Patient Satisfaction in Pre-operative Rehabilitation Practices for Total Joint Replacement

**Published:** 2013-05

**Authors:** M Klatt, L O’Callaghan, F Coelho, A Krakovsky, P Ellison

**Affiliations:** St. Joseph’s Health Centre, Outpatient Rehabiliation Department, Toronto, ON

**Purpose & Objectives / Intention & objectifs:** This paper presents the results of a Quality Improvement (QI) initiative in pre-operative rehabilitation practices at a community based teaching hospital for total joint replacement (TJR) patients and the results of a patient satisfaction survey evaluating the effectiveness of a newly implemented pre-operative education program.

**Relevance/Pertinence:** Ramifications of inadequate pre-operative preparation for elective TJR filter down to the acute care stay potentially delaying discharge and impacting length of stay (LOS). A literature review supported the implementation of pre-operative education for TJR patients as a means to shorten hospital length of stay.

**Materials & Methods/Matériel & méthods:** A comprehensive pre-operative education program for TJR patients was developed following a QI process and implemented March 2012. Data collection occurred for the first 6 months of the program (n=193). Immediately following each education session patients completed a paper based survey.

**Analysis/Analyse:** Descriptive analysis of patient responses to survey questions.

**Results/Résultats:** Compared to the previous fiscal year, average LOS decreased over the study period. Survey response rate was 87%. Close to 100% of respondents reported that they were better prepared for their surgery after attending the session. Moreover, 97.6% reported they would recommend this pre- operative TRJ education program to other patients. **Conclusions:** Results demonstrated overwhelming satisfaction with the pre-operative TJR education program. Ongoing monitoring of LOS and patient satisfaction will be necessary over the first year of the program as modifications are made to delivery methods. A qualitative study of patient perceptions of pre-operative education following surgery and hospital admission would be a helpful in further evaluating the effectiveness of this program.

**Keywords/Mots clés:** Patient Satisfaction, Total Joint Replacement

# Abstract: A030 – Auditing Physiotherapists’ Documentation in an Electronic Medical Record: One Organization’s Experience

**Published:** 2013-05

**Authors:** C Lambert, M Bradshaw, P Miller, A McKnight, T Mihell, T Moies, D Ravenscroft

**Affiliations:** * Hamilton Health Sciences, Clinical Informatics, Hamilton, ON; † Associate Clinical Professor, School of Rehabilitation; McMaster University, Hamilton, ON; ‡ School of Rehabilitation; McMaster University, Hamilton, ON

**Purpose & Objectives / Intention & objectifs:** To examine adherence to regulatory documentation standards among physiotherapists (PTs) working in an academic organization. Findings could inform strategies to promote adherence and improve functionality of the electronic documentation (edoc) system

**Relevance/Pertinence:** Our experience may be of interest to other clinicians and practice leaders developing and/or using edoc systems.

**Materials & Methods/Matériel & méthods:** An audit tool based on regulatory standards was developed. Stratified randomization was used to identify six charts from five different units of patients discharged in February 2012. Two experienced raters completed the audit.

**Analysis/Analyse:** Descriptive statistics were used to identify the frequency and location of documentation events (DE) for admission, follow-up and discharge records. One record could be comprised of several DE using more than one electronic form (eform).

**Results/Résultats:** Across the 30 charts, there were 405 DE. There were 29 initial assessments (97%) documented on six different eforms. 86 % of follow-up DE were on one eform. Four initial assessments were also discharge assessments. Two patients died. Out of 24 remaining patients, eight (33%) had discharge summaries completed. 75% of these summaries were on one eform. Out of 20 eforms, two were consistently used for all aspects of documentation. eForms with directive questions increased adherence to standards. eForms without questions often yielded notes similar to former paper charts. **Conclusions:** Some documentation standards were not well adhered to, however the study sample was very small. Further audits are required. Collaboration between Clinical Informatics Specialists and PTs will address education of staff and revision of eforms in order to promote greater adherence to documentation standards.

**Keywords/Mots clés:** electronic documentation, audit, professional practice

# Abstract: A035 – Guidelines for the Prescription and Fabrication of Aquaplast-T Ankle Foot Orthotics in Children under the Age of 2 years

**Published:** 2013-05

**Authors:** L Benard, K Hurtubise, B Ramage, S Brown

**Affiliations:** Neurosciences, Alberta Children’s Hospital, Calgary, AB

**Purpose & Objectives / Intention & objectifs:** Develop general guidelines for prescription, fabrication and use of physiotherapist (PT) fabricated Aquaplast-T ankle foot orthotics (AFOs).

**Relevance/Pertinence:** A small number of children are prescribed PT-fabricated Aquaplast-T AFOs each year. PT resource constraints, the inability to access training for new staff, and the availability of other orthotic options prompted a review of this practice.

**Materials & Methods/Matériel & méthods:** We used the Context of Care and Practice Framework to guide the process. We reviewed the current literature for evidence, completed a bench marking survey of Canadian facilities, and conducted two formal case studies with representative children.

**Analysis/Analyse:** Results of were reviewed with both the PT and orthotics groups, using a consensus development process. This included an evaluation of the case studies based on fit and alignment, number of modification visits, family preference, and cost.

**Results/Résultats:** The literature review revealed no relevant evidence to support or refute the use of Aquaplast-T AFOs. Of the 10 cross-Canada survey respondents, most utilized custom-made orthotics. PT- fabricated Aquaplast-T AFOs and pre-fabricated orthotics were reported in two other facilities. One family preferred custom-made orthotics, and the second family preferred the pre-fabricated orthotics. Cost benefit analysis revealed that direct cost to the family favored Aquaplast-T AFOs, yet cost to program favored custom made orthotics. **Conclusions:** We developed general guidelines to facilitate decision making by clinicians as to which type of appliance to prescribe. These were based on the clinical presentation of the child, and the amount and direction of support required.

**Keywords/Mots clés:** Physiotherapy made orthotics, Children under 2 years

# Abstract: A037 – Best Practice Principles in Developmental Coordination Disorder: Implications for clinicians and managers

**Published:** 2013-05

**Authors:** C Camden, B Wilson, C Missiuna, A Kirby

**Affiliations:** * CanChild, McMaster University, Hamilton ON; † University of Whales, Newport, UK

**Purpose & Objectives / Intention & objectifs:** Developmental Coordination Disorder (DCD) is a prevalent health condition that remains relatively unknown. Information on efficient management of DCD is growing but is not easily accessible. Few articles are tailored for physiotherapists. The current study identifies all written documents informing best practice in the identification and management of children with DCD.

**Relevance/Pertinence:** Physiotherapists can help children with DCD and their families, but they need to recognize DCD and implement best practice principles. Results will help managers to effectively organize service delivery and will help clinicians to incorporate principles of best practice into their interventions.

**Materials & Methods/Matériel & méthods:** A scoping review was used to ‘map’ the information available to inform practice. Scholarly and grey literature was searched and a ‘snow-balling’ technique was used in Canada and the United Kingdom to access clinical protocols. Over 500 documents were screened: 34 met inclusion criteria.

**Analysis/Analyse:** Data regarding best practice principles for children with DCD were independently extracted and then compared to achieve consistency and consensus.

**Results/Résultats:** Two over-arching themes with six practice principles emerged: organizing services to efficiently meet the holistic needs of children; and working collaboratively to offer evidence-based services. **Conclusions:** System-level implications include the need to increase awareness of DCD and to implement service delivery models with clear pathways and graduated approaches to the intensity of the intervention. Clinical implications include use of strategies to better incorporate the views of children and families, to identify meaningful goals, to use evidence-based interventions that focus on participation, and that prevent secondary consequences. (Slides in English, presentation in French)

**Keywords/Mots clés:** Developmental Coordination Disorder; Service Delivery; Best practices.

# Abstract: A042 – Behavioural Modification in Cardiac Rehabilitation: An Interprofessional Counselling Approach to Nutritional Outcomes

**Published:** 2013-05

**Authors:** D Hopkins-Rosseel, J Wat, M Cooke, Z Patel, L Zaidi, M Shalchi

**Affiliations:** * Queen’s University, School of Rehabilitation Therapy, Kingston, ON; † Hotel Dieu Hospital, Cardiac Rehabilitation Centre, Kingston, ON

**Purpose & Objectives / Intention & objectifs:** To complete a systematic literature review comparing behavioural modification in cardiac rehabilitation (CR) using one-on-one versus interprofessionally-facilitated group counselling approaches for nutritional outcomes and efficiency of care.

**Relevance/Pertinence:** As primary professionals prescribing and evaluating the outcomes of activity and exercise as well as executing interventions to improve cardiovascular risk factors (including obesity, diabetes and dyslipidemias), physiotherapists are ideally positioned to study emerging models of group behavioural counselling.

**Materials & Methods/Matériel & méthods:** The search strategy included 11 databases, using 18 keywords/phrases. Clinical practice guidelines, association’s publications and article reference lists were also screened for relevance. Inclusion criteria included studies in English, with adult participants and behavioural interventions or outcomes in outpatient CR settings.

**Analysis/Analyse:** Identifiers extracted from each article included authorship, year, methodology, subject characteristics, intervention, practitioners and outcomes/results/conclusions. Studies were then assessed independently by 2 reviewers, including being rated from 1a to 5 on the Oxford levels of evidence.

**Results/Résultats:** The search strategy yielded 44 papers, with 13 studies ranked as levels 1a/b; 15-2a/c, and 16 at/or below 3. Although exercise and education group interventions are common in CR and are associated with improved stages of change for diet, exercise and stress, frequency of physical activity and confidence for self-management, studies are limited by the lack of comparison groups and heterogeneity of methods. **Conclusions:** There are insufficient high quality investigations into effective or efficient delivery of behaviour change counselling in CR. We recommend a control study comparing an interprofessional, co-facilitated group counselling model to a one-on-one model of delivery in CR.

**Keywords/Mots clés:** Behavioural modification, cardiac rehabilitation, nutritional counselling, nutritional outcomes, cognitive behavioural therapy, motivational interviewing, patient education, program evaluation.

# Abstract: A061 – What exercises do people with cancer choose when participating in a community-based exercise program?

**Published:** 2013-05

**Authors:** ME Baldner, J Howard, E Jack, G Pepe, O Cheifetz

**Purpose & Objectives / Intention & objectifs:** To determine exercise choices made by cancer survivors participating in a community-based exercise program.

**Relevance/Pertinence:** Exercise is beneficial and safe for cancer survivors and is commonly prescribed by physiotherapists. It is unclear which exercises people with cancer prefer or what exercises would maximize adherence to physiotherapy programs.

**Materials & Methods/Matériel & méthods:** Community-dwelling adults (n=68) with cancer participated in a supervised exercise program consisting of warm-up, resistance, and aerobic exercise twice a week for twelve weeks. Exercise selections were recorded in the participant’s logbook.

**Analysis/Analyse:** The logbook entries were compiled into an Excel file and analyzed using SPSS 20 to determine exercise frequency overall and by cancer diagnosis.

**Results/Résultats:** The most frequently performed exercises were: (1) warm-up: recumbent bike (44.9%), treadmill (44.3%), elliptical (7%); (2) resistance: triceps extensions (11.3%), biceps curls (10.9%), fly (9.8%), row (9.8%), hamstring curls (9.3%), leg press (9.3%), calf raises (8.6%), shoulder raises (5.3%), knee extensions (4.2%), and squats (3.5%); (3) aerobic: treadmill (44%), Arc Trainer® (17%), recumbent bike (14%). Participants with ovarian cancer, multiple myeloma, and lymphoma used the arm ergometer or recumbent bike most frequently. No trends emerged with resistance exercises by diagnosis. **Conclusions:** Participants performed a variety of upper and lower body resistance exercises, and most frequently utilized the treadmill for warm-up and aerobic exercise. There was little variation between cancer diagnoses. These results provide insight into the exercise selections people with cancer make when participating in a community- based exercise program and may contribute to maximizing exercise adherence strategies.

**Keywords/Mots clés:** Exercise, Cancer, Physical Activity, Exercise Preference

# Abstract: A071 – Incorporating research technology into the clinical assessment of balance and mobility: perspectives of physiotherapists and patients with stroke

**Published:** 2013-05

**Authors:** P Pak, B Lamb, C Tirone, H Jawed, K Brunton, A Mansfield, C Cott, L Inness

**Affiliations:** University of Toronto, Department of Physical Therapy and Rehabilitation Institute, 500 University Ave, Toronto, ON

**Purpose & Objectives / Intention & objectifs:** The purpose of this study is to describe the perspectives of patients with stroke and their physiotherapists on the use of biomechanics technology for assessment of balance and mobility.

**Relevance/Pertinence:** Biomechanics technology has been successfully used in research to provide detailed quantitative information regarding balance and gait control post-stroke, but has not been widely incorporated into clinical practice. Both patient and clinician perspectives are important to ensure patient- centered care and the continued improvement of knowledge-translation initiatives.

**Materials & Methods/Matériel & méthods:** A qualitative study was conducted at a large urban rehabilitation hospital. Semi-structured interviews and a focus group were used with adult inpatients with stroke (n=9) who had completed this technological assessment, and their physiotherapists (n=4).

**Analysis/Analyse:** A line-by-line inductive approach was used to analyze the data and to develop codes and themes.

**Results/Résultats:** Patients expressed an interest in having a more extensive explanation of the assessment beforehand. Most patients mentioned fear related to the challenging perturbation-evoked compensatory stepping test, but felt secure due to the trust they placed in their physiotherapist. Physiotherapists expressed challenges interpreting assessment results, but identified evolving skills in this area. The detailed quantitative results were valuable for reinforcing their clinical impressions and tracking change over time, but did not modify their treatment. They stated an interest in incorporating the biomechanics technology in their treatment. **Conclusions:** Regardless of the technological advances in patient care, physiotherapists should continue to establish a strong therapeutic relationship with their patients. Cultivating this relationship through trust and open communication is essential to ensure patients’ sense of security and patient- centered care.

**Keywords/Mots clés:** balance, patient-centered care, qualitative, stroke, communication, assessment, technology, knowledge translation

# Abstract: A072 – Modified Constraint Induced Movement Therapy guideline: Application to the local context

**Published:** 2013-05

**Authors:** M Metzker, D Cameron, S Slen, L Roxborough, T St John, S Tatla, V McCallum

**Affiliations:** * BC Centre for Ability, Vancouver, BC; † Sunny Hill Health Centre, Vancouver, BC

**Purpose & Objectives / Intention & objectifs:** To update an existing guideline and make applicable to local clinicians by providing implementation tools for treating children with hemiplegia.

**Relevance/Pertinence:** Evidence based practice guidelines integrate research, clinical expertise and client input to promote consistent and optimal care delivery.

**Materials & Methods/Matériel & méthods:** A joint partnership between a provincial and regional rehabilitation Centre was initiated in 2010 to update and adapt an existing evidence based guideline to the local context. A multi-professional working group was established to appraise and advance an existing modified Constraint Induced Movement Therapy guideline with current evidence through a comprehensive literature review and local expert consensus. Feedback from stakeholders including community therapists and families was integral to the process.

**Analysis/Analyse:** We evaluated the guideline using the Agree II instrument, performed systematic and critical literature reviews using the American Academy for Cerebral Palsy and Developmental Medicine grading scales and examined current local clinical practices.

**Results/Résultats:** Between 2007 and 2010, 28 new modified Constraint Induced Movement Therapy studies were identified. Our guideline updates include strategies for goal setting and family-centered care, a group protocol and additional outcome measures. Furthermore, we developed implementation tools based on our guideline recommendations and local therapist expertise to support clinicians in administering modified Constraint Induced Movement Therapy. **Conclusions:** This collaborative partnership demonstrates the process of developing and applying a guideline to the local context by integrating best evidence with stakeholder feedback. The adapted guideline, and its accompanying implementation tools, reflect local needs and practice environments, to promote optimal delivery of modified Constraint Induced Movement therapy.

**Keywords/Mots clés:** Modified constraint induced movment therapy, guideline, Agree II

# Abstract: A073 – Reference Values and Psychometric Properties of the Lower Extremity Motor Coordination Test

**Published:** 2013-05

**Authors:** LF Teixeira-Salmela, MB Pinheiro, GC Machado, AC Carvalho, KK Menezes, PR Avelino, CDCM Faria, AA Scianni

**Affiliations:** Department of Physical Therapy, Universidade Federal de Minas Gerais, Belo Horizonte, BR

**Purpose & Objectives / Intention & objectifs:** To establish the reference values and investigate the psychometric properties of the Lower Extremity Motor Coordination Test (LEMOCOT).

**Relevance/Pertinence:** Reference values are useful to interpret the data generated by assessments, to establish realistic goals for interventions, and assess the individuals’ ability to return to their daily activities.

**Materials & Methods/Matériel & méthods:** The LEMOCOT was administered to 320 healthy individuals (50% women and 50% men), who were stratified into seven age groups: 20-29, 30-39, 40-49, 50-59, 60-69, 70-79, and ≥80 years.

**Analysis/Analyse:** Multiple linear regressions, ANOVAS, intra-class correlation coefficients (ICCs), and the Bland & Altman limits of agreement were employed.

**Results/Résultats:** Higher scores were found for men, younger participants, and the dominant lower limb. Age and gender were able to explain about 48% of the variance in the LEMOCOT scores. No significant differences were found regarding the various means of obtaining the scores (the first, means of two and three trials, and the highest scores of the three trials) and all of them demonstrated excellent reliability levels for both the dominant and non-dominant lower limbs (0.91<ICC<0.99). The agreement levels between the scores obtained by direct and video observations was adequate (limits of agreement: -1.99-1.85 and - 1.55-1.62). In addition, appropriate standard measurement errors 1.97-1.54) and smallest real difference values (5.48-4.26) were found. **Conclusions:** The established reference values could be used to estimate the severity of motor coordination impairments in individuals ≥20 years. The LEMOCOT showed adequate psychometric properties. Only one trial was enough to generate reliable scores, and both direct and video observations could be used to score the test.

**Keywords/Mots clés:** Motor coordination, lower limb, assessment, reference values, psychometric properties

# Abstract: A074 – Validity and reliability of the modified sphygmomanometer test for the assessment of muscular strength of subjects with chronic stroke

**Published:** 2013-05

**Authors:** CDCM Faria, LAC Souza, JC Martins, EM Lara, LT Aguiar, AC Carvalho, JB Moura

**Affiliations:** Department of Physical Therapy, Universidade Federal de Minas Gerais, Belo Horizonte, BR

**Purpose & Objectives / Intention & objectifs:** Muscular weakness is the most common impairment in subjects with stroke and the modified sphygmomanometer test (MST) is a promising method to measure strength, since it is both inexpensive and portable. Therefore, the aim of this study was to investigate the concurrent validity and the intra- and inter-rater reliabilities of the MST to evaluate the strength of the trunk, upper and lower limb muscles.

**Relevance/Pertinence:** Physical therapists working with subjects with chronic stroke need an instrument that provides both valid and reliable strength measurements that can be used in clinical settings. The MST has been identified as an instrument that potentially fulfills these requirements.

**Materials & Methods/Matériel & méthods:** The isometric strength of the trunk, upper and lower limb muscles (paretic and non-paretic) of 45 subjects with chronic stroke (57.78±13.30 years) were assessed using the MST and the hand-held dynamometer by two independent examiners in two sessions within a 1–4 week period.

**Analysis/Analyse:** Pearson correlation coefficients were calculated to determine the concurrent validity and the intra-class correlation coefficient (ICC) were used to investigate both intra- and inter-rater reliabilities (α=5%).

**Results/Résultats:** Significant, positive and moderate to high correlations (0.65≤r≤0.95; p<0.01) were found between all the MST and dynamometric measures. Both intra- (0.88≤ICC≤0.98) and inter-rater (0.69≤ICC≤0.97) reliabilities ranged from good to excellent for all of the assessed muscles. **Conclusions:** The low-cost and portable MST showed good concurrent validity and adequate reliability for the assessment of muscular strength and could be applied for clinical and research purposes with chronic stroke subjects.

**Keywords/Mots clés:** Stroke, muscular strength, assessment, modified sphygmomanometer test, dynamometry.

# Abstract: A086 – Validation of the Questionnaire to Identify Knee Symptoms (QuIKS) for assessing early knee osteoarthritis

**Published:** 2013-05

**Authors:** CB Hamilton, MR Monica, BM Chesworth

**Affiliations:** * Graduate Program in Health and Rehabilitation Sciences; † School of Physical Therapy; ‡ Department of Epidemiology and Biostatistics, Schulich School of Medicine & Dentistry, Western University, London, ON; § School of Rehabilitation Science, McMaster University, Hamilton, ON

**Purpose & Objectives / Intention & objectifs:** To create a unidimensional version of the QuIKS using a multidimensional Rasch analysis approach.

**Relevance/Pertinence:** The QuIKS is a new 13-item self-report questionnaire for used by primary health care clinicians to classify patients with early symptomatic knee osteoarthritis symptoms for effective health management.

**Materials & Methods/Matériel & méthods:** People (n=146, aged 39–65 years) with clinically diagnosed knee osteoarthritis with knee pain or knee pain with no knee osteoarthritis diagnosis completed the QuIKS.

**Analysis/Analyse:** Rasch analysis was conducted on the full 13-item QuIKS using a multidimensional approach which accounted for the questionnaire’s four subscale structure. We then assessed the QuIKS for overall fit to the Rasch model, disordered response category structure, item and person fit, local dependency, differential item function by five different characteristics, unidimensionality, internal consistency reliability, and targeting of sample.

**Results/Résultats:** The 13-item QuIKS did not fit the Rasch model. After rescoring disordered response category structure of 10 items and the deletion of one misfit item, the remaining 12 items formed a multidimensional scale which contained the same four subscale structure. These 12 items were determined to form a unidimensional common latent trait. **Conclusions:** This Rasch Analysis support the validity of this 12-item modified version of QuIKS for in use assessing people with early knee osteoarthritis symptoms with or without knee osteoarthritis being confirmed. Thus, the 12-item QuIKS could be useful when creating management strategies for treating individuals with signs of early symptomatic knee osteoarthritis.

**Keywords/Mots clés:** Rasch analysis, knee osteoarthritis, outcome assessment

# Abstract: A095 – Feasibility of a small group neuromuscular electrical stimulation (NMES) upper limb exercise program in stroke individuals?

**Published:** 2013-05

**Authors:** M Trivino, F Kaizer, S Bergeron, J Charbonneau, M Gadoury, V Gendron, MF Levin

**Affiliations:** Jewish Rehabilitation Hospital, Laval, QC

**Purpose & Objectives / Intention & objectifs:** Upper extremity (UE) hemiparesis is a major impairment leading to functional disability following stroke. Electrical stimulation during functional tasks is an effective treatment for UE motor recovery but not often included in therapy because of time constraints. This pilot study aimed to determine whether a small group neuromuscular electrical stimulation (NMES) UE exercise program in stroke individuals was feasible in a rehabilitation hospital setting.

**Relevance/Pertinence:** Group NMES therapy could be effective for increasing therapy time.

**Materials & Methods/Matériel & méthods:** Five individuals with sub-acute stroke participated in a 3-week group program, consisting of four 1 hour sessions/week using the paretic arm in functional tasks with NMES. Participants had UE deficits corresponding to Chedoke McMaster Stroke Assessment scores of 2–3 out of 7 (arm) and 2–4 out of 7 (hand).

**Analysis/Analyse:** Feasibility was assessed through qualitative and quantitative assessment by the administrator, clinicians, and patients. Descriptive statistics highlighted characteristics of facilitators and barriers.

**Results/Résultats:** General patient satisfaction was maintained throughout the intervention according to the results of a questionnaire adapted from Buschfort et al. (2010). However, results from the Short Feedback Questionnaire showed more variation concerning the level of patient appreciation of the intervention. Therapists supported the intervention and identified many facilitators but were concerned by barriers influencing the patient:therapist ratio. Administrator concerns focused on questions about the optimal number of patients per group and the level of severity for inclusion. **Conclusions:** Group NMES therapy was feasible in a rehabilitation hospital setting but depended on group size, patient availability, paresis severity level, and location of the intervention.

**Keywords/Mots clés:** Stroke, Upper Extremity, Neuromuscular Electrical Stimulation

# Abstract: A106 – Elements of Playground Design to Enhance Participation in School-Aged Children with Developmental Disabilities

**Published:** 2013-05

**Authors:** A Carlucci, P Dinunzio, A Laverdière, Z Lin, M Park, C Perlman, R Todor

**Affiliations:** McGill University, School of Physical & Occupational Therapy, Montreal, QC

**Purpose & Objectives / Intention & objectifs:** To identify which elements of a playground design most influence participation of children with developmental disabilities with all levels of cognitive, sensory, motor, and behavioural abilities

**Relevance/Pertinence:** Playgrounds exist in all communities and represent accessible opportunities for social, motor, cognitive, and sensory stimulation for children. Maximizing participation in these environments is therefore crucial for the overall development of children with developmental disabilities.

**Materials & Methods/Matériel & méthods:** Target populations included students between 5 and 12 years old attending a school for individuals with mild-to-moderate developmental disabilities, their parents, and their professionals (occupational/physical therapists, speech language pathologists, physical education teachers, teacher consultants, and teachers). Observation of students using the outdoor school playground was conducted over two mornings. Questionnaires were sent to 105 parents. Finally, two focus groups were conducted, one involving nine professionals and the other four parents.

**Analysis/Analyse:** Triangulation and thematic analysis were utilized to analyze the data from all methods. An iterative process was then used to classify the emerging themes according to importance.

**Results/Résultats:** Four themes emerged: skills required to play, social interaction and inclusion, safety, and sensory experiences. The most common theme overall related to skills, but the most important theme from the parent focus group related to social interactions. **Conclusions:** When designing new playgrounds, consideration for elements relating to skills, social, safety, and sensory components can strengthen their design and usability. Moreover, skill-related elements were identified as the most important elements because, in order to participate in an environment, children need to be capable of accessing and “using” the environment.

**Keywords/Mots clés:** Participation, developmental disabilities, playground

# Abstract: A013 – An evidence-based approach to the development of an interdisciplinary clinical scenario

**Published:** 2013-05

**Authors:** P Miller, EL Geddes, J Southam, J Koopman, T Sun

**Purpose & Objectives / Intention & objectifs:** This study examined how learning objectives (LOs) generated by rehabilitation students using a newly-developed clinical scenario (CS) aligned with anticipated LOs identified by the developers. CSs are often based on real, client-centered situations, but there is a paucity of evidence about CS development.

**Relevance/Pertinence:** Findings related to developing this CS may assist educators in optimizing student learning and in curriculum development.

**Materials & Methods/Matériel & méthods:** A new CS addressing falls prevention among community- dwelling seniors was developed collaboratively by experienced homecare clinicians, tutors, academics/educators and the investigators. Senior occupational therapy (OT) and physiotherapy (PT) students participated in two mock interprofessional tutorials conducted by an experienced tutor. Students discussed the CS and identified LOs. Tutorial sessions were audiotaped and transcribed; whiteboard notes were photographed.

**Analysis/Analyse:** Qualitative content analysis of the transcripts and notes was conducted to determine the extent to which the CS prompted discussion and identification of the anticipated LOs.

**Results/Résultats:** Nine students (7 PTs, 2 OTs) participated. Both student groups identified three of the six LOs. One group identified an additional LO. Two LOs not identified were discussed briefly with probes from the tutor. Unfamiliarity with the interprofessional tutorial setting was noted as a challenge by some students. **Conclusions:** This carefully-developed CS generated some but not all of the anticipated LOs, highlighting the need to carefully examine and test CSs prior to implementation. The CS has been revised and has the potential to be used in a broader interprofessional student context or with novice clinicians.

**Keywords/Mots clés:** problem-based learning, curriculum development, fall prevention

# Abstract: P004 – Knowledge translation: Identifying and overcoming barriers to knowledge use

**Published:** 2013-05

**Authors:** J Miller, J MacDermid, Hand and Upper Limb Centre, L Brosseau

**Affiliations:** * School of Rehabilitation Science, McMaster University, Hamilton, ON; † St Joseph’s Health Centre, London, ON; ‡ School of Rehabilitation Sciences, University of Ottawa, Ottawa, ON

**Overview & Objectives/Résumé & objectifs:** By the end of the session, participants will be able to:

1. Identify potential barriers and facilitators to knowledge use
2. Discuss the importance of knowledge translation strategies in overcoming barriers to knowledge uptake and utilization
3. Describe how to identify knowledge to action gaps, evaluate potential barriers and facilitators to knowledge use, and knowledge translation strategies to overcome these barriers.

These three objectives will be achieved through discussion of theory and current best-evidence on knowledge translation and through sharing the authors’ experiences developing, implementing, and evaluating three knowledge translation strategies. First, McMaster Premium Literature Service (macPLUS) provides a searchable database of the best evidence from health care literature, an email alerting system, and links to selected evidence-based resources. This knowledge translation strategy, which is currently being investigated in a randomized controlled trial, has been developed to meet the challenges faced by clinicians in effectively searching and evaluating research to answer questions applicable to their patients. Second, People Getting a Grip on Arthritis is a knowledge translation strategy aimed at helping people with rheumatoid and osteoarthritis become more aware of and utilize clinical practice guidelines as they relate to self-management strategies. People Getting a Grip on Arthritis has been evaluated to determine its influence on participant knowledge, skills, and self-efficacy regarding the uptake of clinical practice guidelines as well as participant intention and actual use of clinical practice guidelines. Finally, Manual Therapy and Exercise for Neck Pain: A clinical treatment tool-kit is a knowledge translation tool designed to provide clinicians with the knowledge from 3 systematic reviews and 1 overview regarding treatment options for neck pain in an easy to use and understand format. Barriers and facilitators to its use in a rehabilitation setting have been evaluated using cognitive interviews. Each of these three knowledge translation strategies have involved identifying knowledge to action gaps, recognizing potential barriers and facilitators to knowledge use, and attempting to overcome any barriers to implementation of best-evidence into clinical decision making.

**Relevance/Pertinence:** There is increasing recognition of the importance of evidence based practice in physiotherapy. Research available to inform physiotherapy practices is growing, which provides an opportunity for improving patient care and outcomes. In order for research to improve patient care and outcomes, however, it must be incorporated into clinic decision making. It is important for physiotherapists, practice leaders, and researchers to understand how to identify and overcome potential barriers and facilitators to using evidence in practice. An understanding of how to recognize and overcome barriers to evidence based practice will allow more effective knowledge translation strategies that encourage incorporating the best evidence along-side clinical expertise and patient values in clinical decision making.

**Target Population/Population cible:** This session will provide a learning opportunity for a varied audience including clinicians, managers, practice leaders, educators, and researchers who are interested in increasing the utilization of best practices through knowledge translation initiatives.

**Supporting Evidence/Résumé des faits:** Evidence based practice is the conscientious, explicit and judicious use of current best evidence in making decisions about the care of individual patients (Sackett et al 1996). Despite an increasing volume of evidence available to inform clinical decision making, evidence to practice gaps remain common in the healthcare system (Schuster et al 1998; Asch et al 2006). Evidence suggests the use of active, multi-component knowledge translation interventions can change practice behaviours, but that the effectiveness of knowledge translation strategies vary based on the context in which they are provided ((Menon et al 2009; Larroca R et al 2012). It is important that we understand the context in which the health care is being provided, including any potential barriers or facilitators that may be present. With knowledge of these potential barriers and facilitators can we optimize the effectiveness of our knowledge translation strategies and increase evidence based practice in physiotherapy.

**Session Format/formule de la session:** This session will include three presentations in a lecture format followed by a discussion period during which participants will be encouraged to ask questions and discuss their own knowledge translation experiences

**Conclusions & implications:** Improving evidence based practice in physiotherapy has the potential to improve the health and quality of life of the people receiving care from physiotherapists. Increasing research alone is not enough to improve patient outcomes, however. Knowledge translation strategies aimed at increasing uptake of best-evidence in physiotherapy practice are important. In order for these strategies to be effective, researchers, clinical practice leads, managers, and physiotherapists must collaborate in order to identify barriers and facilitators to the use of the knowledge in a given context. Understanding barriers and facilitators to knowledge use will aid in the development and implementation of knowledge translation strategies that help clinicians incorporate the best available evidence, clinical expertise, and patient values when making shared health care decisions with their patients.

**Keywords/Mots clés:** knowledge translation, patient education, knowledge transfer

# Abstract: P005 – Knowledge translation in the trenches: how to unite evidence, theory and experience to enhance evidence-informed practice using the example of a kt initiative to enhance clinical management of tendinopathy

**Published:** 2013-05

**Authors:** A Hoens, A Scott

**Affiliations:** * Physical Therapy Knowledge Broker and Clinical Professor; † Assistant Professor; ‡ Department of Physical Therapy, University of British Columbia, Vancouver, BC

**Overview & Objectives/Résumé & objectifs:** Learning Objectives

This session will focus on key steps which are important when undertaking a knowledge translation (KT) project. The presenters will lead participants through the KT process by sharing the evidence, theory and experiences underpinning a successful KT initiative to enhance evidence-informed clinical management of tendinopathy. The process followed in developing and disseminating the ‘Tendinopathy Toolkit’ will be used to highlight important components inherent in the knowledge to action cycle (KTA cycle).

Following this session, participants will be able to:

1. Describe the key components for an effective knowledge translation initiative based on the Canadian Institute for Health Research (CIHR) supported KTA cycle.
2. Understand how theories of change and evidence from the field of implementation science can be utilized to inform a KT initiative.
3. Plan, using the principles presented in the course and illustrated with the example of the Tendinopathy Toolkit, a KT initiative.

**Relevance/Pertinence:** Given the evidence that it takes approximately 17 years to get 14% of research findings adopted into health care practice (Westfall et al, 2007) it is imperative that physical therapists incorporate theory and evidence in their efforts to transform clinical practice. Typical efforts, e.g. didactic education sessions, feedback and reminders, have demonstrated minimal change in practice patterns. Accordingly, physiotherapy practice leaders, clinicians, educators and researchers would benefit from some pragmatic guidance in how to undertake a KT activity which, based on evidence and theory, would have a greater potential to enhance evidence-informed practice.

**Target Population/Population cible:** This education session will be of interest to researchers, educators, practice leaders and frontline clinicians who are involved in activities that are relevant to enhancing evidence-informed practice.

**Supporting Evidence/Résumé des faits:** Description of supporting evidence Without widespread accessibility to training in the principles and the application of evidence-informed strategies to support practice change there continue to be significant barriers to adoption of best evidence. However, there is increasing evidence that greater success in practice change is possible through the use of theoretical frameworks (Aijzen, 1991; Rogers, 2003; Grol Wensing & Eccles, 2005) and strategies informed through implementation science (O’Brien et al, 2007; Rivard et al, 2010). Moreover, pragmatic guidance (Graham, 2006) and an increasing number of resources (Straus et al, 2009; Barwick, 2010) are now available to assist those who seek to improve their knowledge translation activities.

**Session Format/formule de la session:** Description of session format This session will utilize both lecture (with opportunities for interaction by participants) and small group formats. The lecture portion will provide participants with the relevant knowledge of the theories and evidence underpinning KT - specifically following the KTA cycle of knowledge creation/synthesis, dissemination, implementation, evaluation and sustainability. The learning will be supported with the presentation of the process undertaken in a recent KT initiative by a unique partnership of physical therapy researchers, educators and expert clinicians to address the gap between evidence and practice in the management of Achilles tendinopathy. Following the lecture component, participants will work in small groups, facilitated by the session faculty, on a worksheet designed to guide the planning of a KT activity using the steps of the KTA cycle.

**Conclusions & implications:** Conclusion Physical Therapy researchers, educators, practice leaders and clinicians struggle with the challenges of changing practice to incorporate recent evidence. An appreciation of the theoretical frameworks and evidence from the implementation science literature, together with lessons learned from successful KT iniatives guided by the KTA cycle, can enhance the effectiveness of efforts to support evidence-informed practice.

**Keywords/Mots clés:** knowledge translation; musculoskeletal; tendinopathy

# Abstract: P006 – The Health Practitioner’s Guide to Motivational Interviewing and Change Talk: How to foster a patient-centred approach for behavior change in your clinic

**Published:** 2013-05

**Authors:** K Houde, T Yardley

**Affiliations:** * University of Ottawa Health Services, Health Promotion Department, Ottawa, ON; † CBI Health Group, Courtenay, BC

**Overview & Objectives/Résumé & objectifs:** By the end of this session, participants will be able to:

1. Understand motivational interviewing research and concepts and be able to apply them within a physiotherapy setting
2. Evaluate how the use of motivational interviewing can strengthen the therapeutic alliance between patient and therapist
3. Identify key motivational interviewing resources to assist them in further developing their motivational interviewing skills

**Relevance/Pertinence:** Motivational Interviewing (MI), first introduced by Miller in 1983 to treat individuals with substance disorders, is defined as a “collaborative, person-centered form of guiding to elicit and strengthen motivation for change”. This approach has been proven through systematic reviews to outperform traditional advice from doctors, nurses and counselors when dealing with patients who are dealing with lifestyle problems and disease such as alcoholism, smoking and obesity. While far less studies exist to show the effectiveness of this approach in the rehabilitation setting, there is still significant evidence to support the use of MI in a number of client populations with whom physiotherapists interact, such as patients with musculoskeletal problems, cardiac and stroke patients, injured workers, and those dealing with chronic pain. Most physiotherapists use some form of motivational interviewing in their communication with patients. However, when time is short, and patients are presenting with more chronic or complex conditions, it can be quite easy to lapse into a style of communication that is more directive and does not encourage the patient to adhere to treatment goals. Studies show that therapists who receive training in MI demonstrate increased confidence when using this approach with patients, and that this approach is successful in producing positive behavior change for clients. Physiotherapy best practices encourage a more patient-centered approach to treatment. While motivational interviewing has demonstrated success in medical settings to assist patients in adopting positive behavior changes, this approach is not as commonly used in physiotherapy practice. This session will serve to demonstrate that motivational interviewing can be successfully incorporated within the rehabilitation setting to produce positive outcomes for both the therapist and the patient.

**Target Population/Population cible:** This session will be of interest to any clinician working in public or private practice who is actively treating patients and is interested in using an approach that will influence patient engagement in and adherence to treatment. No prior experience or training in motivational interviewing is required.

**Supporting Evidence/Résumé des faits:** Significant evidence exists to support the efficacy of MI in its ability to outperform traditional advice in the treatment of behavioral problems and disease. Systematic reviews evaluating MI in more traditional settings (Rubak et al, 2005) indicate that MI promotes positive behavior change in patients. The efficacy of MI use in physical health settings is not yet well established (Knight et al, 2006) but the findings do support the efficacy of motivational interviewing in increasing client motivation, and that MI research in the rehabilitation setting is “worthy of further exploration” (Wagner and McMahon, 2004). Supporting evidence for MI principles, training and best practices will be drawn from a number of sources such as research and training manuals developed by pioneers of MI such as Rollnick and Miller, guidelines for practitioners developed by organizations such as the National Institutes of Health’s National Institute on Alcohol Abuse and Alcoholism, as well as more recent studies that evaluated the efficacy of different MI training approaches on therapist adherence and competence (McGraw et al, 2009) and optimal MI session duration and frequency (Cochrane review, 2010) in influencing patient behavior change, and positive treatment outcomes.

**Session Format/formule de la session:** This session will be delivered via interactive lecture, and live or web- based demonstrations. It will also include breakout sessions where participants will be encouraged to practice motivational interviewing skills and techniques, further enhancing their experiential learning.

**Conclusions & implications:** Motivational interviewing is proven to be effective in helping patients change negative behaviors in the areas of substance abuse, obesity, and smoking cessation. There are fewer studies that show its effectiveness in the rehabilitation setting, although results to date in the areas of cardiac rehab and musculoskeletal health are promising. By understanding MI principles and strategies and using resources and tools to stimulate lifelong practice, therapists can learn to effectively use motivational interviewing to identify their patient’s readiness to change, develop treatment goals in collaboration with their patient, strengthen the therapeutic alliance, improve patient adherence to treatment, and ultimately produce better outcomes for both the therapist and the patient.

**Keywords/Mots clés:** motivational interviewing, change talk, stages of behavior change, therapeutic alliance, treatment outcomes, patient-centred care, compliance, adherence

# Abstract: P007 – Diagnosis and Management of Distal Biceps Tendon Ruptures

**Published:** 2013-05

**Authors:** M Devereaux, K Quan-Velanoski

**Affiliations:** * Fluid Motion Physiotherapy, Mahone Bay, NS; † St. Joseph’s Health Centre, Outpatient Physiotherapy, Toronto, ON

**Overview & Objectives/Résumé & objectifs:** At the end of the session, participants will be able to:

1) Visualize and describe the anatomy and structures affected by distal biceps tendon injury; 2) demonstrate an efficient clinical assessment protocol - including four physical examination manoeuvres- to reliably diagnose instances of complete distal tendon rupture; 3) determine the treatment implications of the diagnostic results; and 4) outline an appropriate rehabilitation protocol for patients with a surgically repaired distal biceps tendon.

**Relevance/Pertinence:** Prior to 1995, there were only 53 published articles on injury to the distal biceps tendon. Since 2007, more than 70 new reports on the topic have been published, suggesting an increasing frequency in injury incidence and detection. There are numerous clinical papers reporting on the classic signs and symptoms of distal biceps tendon rupture, however not all patients present with these characteristic findings. As a result, missed or delayed diagnoses persist. The ability to distinguish complete from partial tears is critical, as the urgency and nature of treatment options differ significantly. As frontline clinicians, physiotherapists may be the first to assess patients with distal biceps tears, and must be able to identify and distinguish these injuries in order to facilitate rapid surgical referral in cases of complete rupture. Clinicians therefore require a standardized, efficient and reliable clinical examination protocol that can accurately identify cases of complete rupture.

**Target Population/Population cible:** This session will be of interest to orthopaedic and sports medicine clinicians with an interest in evidence-based clinical examination techniques.

**Supporting Evidence/Résumé des faits:** Diagnosis of complete distal biceps tendon rupture is frequently missed or delayed on clinical examination. No one clinical test, including MRI, has demonstrated 100% efficacy in assessing the integrity of the distal biceps tendon. Using a prospective cohort study of 48 patients with suspected distal biceps tendon injuries, we evaluated the diagnostic efficacy of combining three validated clinical tests for identifying complete rupture, in concert with other important factors from the patient history and physical exam to maximize a true positive diagnosis for complete distal biceps tendon rupture without the need for confirmatory soft tissue imaging. This session describes these physical examination techniques as part of an overall assessment algorithm to reliably diagnose instances of complete distal biceps tendon rupture. We also present a post-operative rehabilitation protocol based on the functional outcomes of thirty three patients who underwent primary surgical repair of the distal biceps tendon.

**Session Format/formule de la session:** This session will be an interactive lecture format, including video demonstration of physical examination and rehabilitation techniques.

**Conclusions & implications:** Application of our clinical assessment protocol for identifying patients with complete rupture of the distal biceps tendon can facilitate rapid referral for patients most likely to benefit from surgical repair, and indicate when conservative management is appropriate in cases of partial rupture. Application in sequence of the physical examination manouevres outlined in the protocol results in 100% sensitivity and specificity when the outcomes on all three physical tests are in agreement.

**Keywords/Mots clés:** distal biceps; tendon rupture; elbow; avulsion

# Abstract: P008 – An evidence-informed clinical reasoning approach to managing complex orthopaedic cases

**Published:** 2013-05

**Authors:** E Yeung, L Levesque

**Affiliations:** * University of Toronto, Department of Physical Therapy; † Western University, Physiofirst Orthopaedic and Sports Centre

**Overview & Objectives/Résumé & objectifs:** The primary aims of this workshop are to provide participants with an understanding of the key elements of evidence-based practice as applied to the musculoskeletal context, and to provide practical tools that will enable and support participants to engage in an evidence- based approach to clinical decision-making.

Upon completion of this workshop, participants will be able to:

1. Discuss the key elements of evidence-based practice in physiotherapy practice
2. Apply an evidence-based approach to a complex musculoskeletal case
3. Apply specific tools to facilitate an evidence-based approach to clinical reasoning

Evidence-based practice has been defined as the integration of the best of research knowledge with clinical experience and patient values [Sackett 1996]. Thus, an evidence-based clinical reasoning approach must consider multiple sources of knowledge in the clinical decision-making process including that from the patient and the clinician (Akobeng 2005). Sackett’s conceptual framework of evidence-based practice will form the basis for the interactive activities and discussion for this workshop. Without deliberate and explicit cognitive and metacognitive practice, integration of all the relevant sources of knowledge can often pose as a challenge to the clinician, especially in complex cases where ‘evidence’ may be ambiguous or conflicting. This workshop will focus on the use of specific reflective exercises that facilitate critical thinking and metacognitive practice while attending to the different components of Sackett’s model for evidence-based practice. For example, we will use the Rehabilitation Problem Solving Form (Steiner 2002) to assist participants to distinguish between the perspectives held by the patient and those of the health care professional, to capture the constructs within the International Classification of Function and Disability framework and to promote a biopsychosocial perspective in their clinical decision- making. In addition, we will engage participants in reflective practices that will foster reflection upon their own clinical experiences; identifying and testing hypotheses that are both consistent and inconsistent with cases from their clinical experiences.

**Relevance/Pertinence:** Critical and advanced levels of clinical reasoning skills are important to physiotherapy practice. This is evidenced by a number of Canadian post-graduate programs targeted at advancing practice through explicit development of clinical reasoning skills. For example, advanced levels of clinical reasoning are not only one of the desired outcomes of the Canadian Physiotherapy Association clinical specialty program (CPA), but this is also one of the core education standards identified by the International Federation of Orthopaedic Manipulative Physical Therapy (IFOMPT) for post-graduate manipulative physical therapy programs around the world (IFOMPT standards document 2008). This workshop provides the aforementioned target audience an opportunity outside of formal training programs to develop an evidence-based approach to clinical decision-making.

**Target Population/Population cible:** The content of this workshop will appeal to educators, physiotherapists, and physiotherapy students at all levels of their career who have an interest in developing their clinical reasoning skills. Although the content will focus on an orthopaedic case, the knowledge and skills gained through this workshop will be applicable across other practice areas in physiotherapy.

**Supporting Evidence/Résumé des faits:** ‘Evidence’ has traditionally been discussed and understood as research evidence (Forbes 2012). While this is an important component of evidence-based practice, an over- emphasis on this component can devalue, and therefore limit the inclusion of ones ‘clinical experience’ and the patient’s perspective. Rather, a more holistic approach that takes into account all components of Sackett’s evidence-based practice model will likely lead to better patient outcomes. Strategies that foster reflection upon the constituents of evidence-based practice can be one way to improve a clinician’s cognitive and metacognitive processes to assist in managing complex patient cases.

**Session Format/formule de la session:** The workshop will be structured such that the participants will be involved in problem-solving activities related to a specific case history. Using a reflective practice approach, this workshop will emphasize problem-solving and active learning in order to engage workshop participants in discussions about a complex orthopaedic musculoskeletal case. Participants will have opportunities to work in small groups throughout the workshop. Different components of the case will be presented via video or PowerPoint presentation and through group discussions to allow participants to work through the clinical-decision making process.

**Conclusions & implications:** An evidence-based approach to clinical decision-making is a complex process requiring the physiotherapist to integrate multiple sources of knowledge including research knowledge, knowledge of the patient’s perspective and one’s clinical experience. This workshop affords workshop participants a forum for exploration and reflection upon all of these different components in order to improve workshop participants’ understanding and application of the key elements of evidence-based practice.

**Keywords/Mots clés:** Orthopaedics, Evidence-based practice, clinical reasoning

# Abstract: P015 – Distal Radial Fractures in Women – A Fracture Forgotten and a Window for Prevention

**Published:** 2013-05

**Authors:** C Arnold, K Crockett

**Affiliations:** * School of Physical Therapy, University of Saskatchewan, Saskatoon, SK; † College of Medicine Health Sciences Program, University of Saskatchewan and Bourassa and Associates Rehabilitation Centre, Saskatoon, SK

**Overview & Objectives/Résumé & objectifs:** Fractures to the distal radius (wrist) are one of the earliest fragility or minimal trauma fractures to occur in women and therefore is a window of opportunity to address preventative measures to screen and prevent future fall and fracture risk. Unfortunately, women with wrist fractures are not routinely screened for future fall and fracture risk, and may only be sent home with post- cast removal instructions for range of motion exercise rather than be followed for more comprehensive assessment and treatment by a rehabilitation specialist. The purpose of this session is to address the important role physical therapists and other health care providers can provide in assessing and treating the future risks for falls and fragility fracture associated with this common early post-menopausal injury. The causes, biomechanical, physiological, environmental and social factors associated with wrist fracture have not been widely studied, nor is there a clear understanding of the efficacy or role of rehabilitation practice in the functional recovery and prevention of future fracture. We will provide an evidence-based overview of the causes, the biomechanics associated with falling on the outstretched hand, assessment of bone status, functional and fall risk outcomes, the functional recovery in the first year post-fracture and the current state of evidence supporting physical therapy treatment. We will focus on best practice rehabilitation prevention and management for this common injury in older women as well as current research related to musculoskeletal clinical practice. Specifically, after this session, the learners will be able to:

1. Describe the epidemiology, mechanisms and causes of falling on the outstretched hand and the factors influencing serious injury.
2. Relate evidence of functional recovery post-fracture to assessment and treatment choices given specific case histories.
3. Apply knowledge of current fall and fracture risk screening and assessment practices to this population

**Relevance/Pertinence:** Physical therapists commonly see women who have fractured their wrists either as outpatients or in acute care settings. However, the face to face contact may be minimal or not at all, with many patients provided with a set of home exercises to follow on their own. Assessment for osteoporosis and fracture risk is not routinely followed nor are physical therapists necessarily aware of simple prediction tools they can utilize to assess this. Physical therapists and other health professionals are well versed in the role they can play for fall and fracture prevention in adults over the age of 65 years, they may be less aware of the needs for women in their 50s and early 60s. The rehabilitation approach to assessing and treating women post wrist fracture is varied across Canada, and there are no clear evidence-based guidelines to provide best outcome for these women or to prevent future fracture. Understanding the causes, biomechanics, and functional status associated with this type of injury will assist clinicians to apply appropriate assessment and treatment protocols.

**Target Population/Population cible:** This presentation is designed to be applicable to a wide audience including Physical Therapists and other Health Care Providers (i.e. nurses, physicians, occupational therapists, exercise and recreational therapists), Researchers, Funders, Policy-Makers, Consumers.

**Supporting Evidence/Résumé des faits:** The reasons why still remain unclear, but women are 4 – 6 times more likely than men to fracture their wrist, most commonly by falling on an outstretched arm. This could be due to muscle strength differences in the upper arm, more fragile bone during this period of hormonal change, or other unknown fall and fracture risk factors. Observational studies of falling events have found that falling on the outstretched hand can result in other serious injury such as head injury. A case control study found that older women have a diminished ability to control body descent and energy absorption as compared to younger women. This could be due to limitations in muscle strength in controlling the body’s descent of the fall; however there are no studies to support this conclusion and little is known about the biomechanics of this frequent fall event. Retrospective and longitudinal studies have found that a wrist fracture is the most common fragility (minimal trauma) fracture in women, and typically occurs 10 – 15 years earlier than other osteoporotic fractures. History of one or more fragility fracture after age 40 years is one of the strongest predictors of future fracture, including hip fracture, an injury that carries substantial personal and societal costs. In fact, older women aged 50 – 65 years who had sustained a wrist fracture had a three- fold increase in risk of future fracture within the next three years. This presents a window of opportunity for health care providers to prevent future fracture. Determining the appropriate functional and fall risk assessment to use for this population who are typically still working and very active, is challenging. New, easily accessible fracture risk prediction tools are becoming more widely used by physicians, but other health professionals may not be as aware of their utility, particularly in the absence of bone mineral density values. Rehabilitation specialists are experts in functional assessment, including fall risk assessment, and therefore are an important health care professional to identify future risk. However, cross sectional studies would suggest that frequently follow-up for fracture and fall risk assessment is missed, despite the knowledge that one fragility fracture is a high risk factor for another to occur in the future. Although physical therapy is recognized as an important rehabilitation component of post wrist fracture care, the role that they could play in future fall and fracture prevention for these patients should be acknowledged. Unfortunately, in one systematic review of rehabilitation management post-fracture, there was inconclusive evidence to provide any best practice guidelines for PT treatment post- wrist fracture, and little research has described the length of functional recovery and the long term impact of incomplete recovery on future functional ability and fracture risk.

**Session Format/formule de la session:** The format of this presentation will include primarily a lecture presentation, including knowledge translation from original research conducted by our research team in bone health and fracture prevention. The session will be completed with some group discussion and application to cases.

**Conclusions & implications:** Wrist fracture in women during their early, active senior years should be a red flag to the health care team that a future injury and potential fracture is imminent. This provides one of the greatest opportunities to deliver preventative measures that could have a significant impact of reducing the growing cost of falls and fragility fracture in our aging Canadian society. By understanding the causes of the injury, the fracture and fall risk factors preceding the injury, and the types of risk assessment that may help to prevent future fracture, physical therapists may be able to assist women to prevent a more devastating osteoporotic fracture such as a hip fracture later in life. The economic cost of fractures in older adults continues to rise, and in the next 10 – 20 years we will need to address this health care crisis.

**Keywords/Mots clés:** fracture, falls, biomechanics, risk factors, assessment, osteoporosis, female

# Abstract: P017 – Inspiratory muscle training to facilitate weaning from mechanical ventilation - a unique approach

**Published:** 2013-05

**Authors:** JL Kay

**Affiliations:** St Clare’s site of Eastern Health, St. John’s, NL

**Overview & Objectives/Résumé & objectifs:** Mechanical ventilation provides the respiratory system with effective support, when needed, to address respiratory failure from a variety of sources. It can also have the negative effect of decreasing muscle strength and endurance when the need for support is prolonged. Failure to wean has been defined as greater than 15 days of ventilation. As well as individual patient impact of prolonged ventilation, increasing costs to health care are anticipated. United States figures project care of ventilated patients is expected to reach more than $50 billion in the next decade.Thus for many reasons this is an area of ICU care that requires innovation and the application of best practice. At the end of this presentation, participants will be able to: 1) describe the impact of critical illness and mechanical ventilation on the respiratory muscles, with particular emphasis on the diaphragm; 2) identify a subset of patients who may benefit from inspiratory muscle training and 3) create a threshold muscle training option by prescribed, progressive changes of the sensitivity setting on the mechanical ventilator while localizing a diaphram breathing pattern during inspiratory muscle training sessions. This includes measurement of maximum inspiratory pressure.

**Relevance/Pertinence:** Muscle training to regain independent functional power and endurance are hallmark areas of strength for physiotherapists. Deconditioning occurs in muscle groups in general in the critically ill patient and can be significant for respiratory muscles. Patients’ lengths of stays in ICU are protracted as various approaches to weaning from the ventilator prove to be unsuccessful. As physiotherapists, we bring a unique perspective and body of knowledge regarding muscle training. It can be extrapolated first to this critical care setting and then across the continuum of care as the mode of muscle training can be progressed to a portable devise for hospital, rehabilitation or on discharge, to home settings. Implementing inspiratory muscle training as described demonstrates the application of best practice, enhances our role within the ICU team for select patients and enables us to optimize the use of the ventilation technology.

**Target Population/Population cible:** While the information may initially have relevance for physiotherapists practicing in intensive care, the material presented regarding the muscular impact of critical illness and mechanical rest has a broad appeal. The concepts apply to those multisystem, multi impaired patients who are or have recently been discharged from a critical care unit. The information has relevance to physiotherapists working across the spectrum from acute care to rehabilitation and home care for patients with inspiratory muscle dysfunction.

**Supporting Evidence/Résumé des faits:** The sources for muscle weakness in the critical care setting are multifactorial, as Nava and Fasano (2011) outline. They include disuse atrophy secondary to the effects of positive pressure ventilation modes, electrolyte changes, impact on inotropic properties by some medications, as well as the presence of co-morbities such as chronic obstructive pulmonary disease, (COPD). The evidence as to whether inspiratory muscle training has an impact on improved weaning from mechanical ventilation is very limited. One recent systematic review of 3 studies concluded larger studies were needed as the data was insufficient to make a recommendation. Small sample case studies make up the majority of the small body of published evidence. The training approaches used are not consistent, with variation from periods of spontaneous breathing to disconnecting from the ventilator circuit to use a Threshold or flow resistive muscle trainer. One article was published using changes in the sensitivity setting as the training mode, based on achieving 6 to 8 on a scale of perceived dyspea. No studies were found that directed patients to attempt focused diaphragm recruitment during the resisted training sessions. Given the degree of resistance to descent of the diaphragm, it relies on recruiting other muscles to assist inspiration when unable to meet the demand. Yet no article regarding inspiratory muscle training was identified that addressed this issue. A recently published study in a neuroscience journal (de Rugy,2012) raised an interesting thought around this issue. When assessing the muscle response to paralysing one wrist muscle, for example, the subjects recruited all muscles to accomplish the task rather than only the useful muscles. This lack of isolation of muscle input when training may be a factor limiting the success to date of inspiratory muscle training programs in the mechanical ventilation setting.

**Session Format/formule de la session:** The session will be multimodal in presentation. Didactic presentation of material is used regarding respiratory muscle physiology, pathophysiology and impact of mechanical ventilation as well as demonstration, patient video and a short practice session for the attendees.

**Conclusions & implications:** The presentation lays the foundation for the need for threshold muscle training to facilitate respiratory strengthening following periods of prolonged mechanical ventilation. It supports the possibility of creating a threshold muscle training capacity by changing the sensitivity settings on ventilators as determined by maximum ispiratory pressure levels. A progressive training effect can be achieved and increased inspiratory pressure generated to enable successful weaning from ventilation support. The clinical example provided demonstrates the applicability for spinal cord injury patient; a broader based application of the technique has successfully been used by this therapist for a variety of conditions resulting in decreased respiratory muscle power and endurance. This approach has implications for increased physiotherapy involvement in the achievement of optimal performance of the respiratory muscles from a continuum that starts in the intensive care setting and continues to discharge. It broadens interdisciplinary collaboration in the delivery of best practice to achieve a common goal and strengths the position of physiotherapy in the acute care setting.

**Keywords/Mots clés:** Mechanical ventilation, inspiratory muscle training

# Abstract: P020 – What is ’recovery’, and is it really clinically achievable?

**Published:** 2013-05

**Authors:** WM Walton

**Affiliations:** School of Physical Therapy, Western University Canada, London, ON

**Overview & Objectives/Résumé & objectifs:** Overview:

While Physiotherapists are increasingly recognizing the value of outcomes measurement, the notion of recovery and how it should be defined remains elusive. This will be an interactive workshop in which participants will be challenged to consider the nature of recovery, including its operationalization, measurement, and even its appropriateness as a rehabilitation target. The proposition will be forwarded that recovery as it has been traditionally defined is an unreachable target, using Leventhal’s Common Sense Model of Illness Representations. Alternative operationalizations of satisfactory rehabilitation end-points will be described, and participants will be given the opportunity to reflect on these issues and how they affect their clinical practice.

Learning objectives:

1. To understand the Common Sense Model of Illness Representations
2. To critically reflect upon current conceptualizations of recovery through the lens of traditional and new knowledge
3. To discuss different options for defining an appropriate end point of a physical rehabilitation program
4. To understand how satisfaction, happiness, or quality of life can be acceptable alternatives, and measurable, outcomes of physical rehabilitation

**Relevance/Pertinence:** Current understandings of recovery, as a return to some pre-injury state, is conceptually impossible from constructivist and Common Sense philosophical paradigms. However, this notion of ’going back in time’ to pre-morbid status informs much of physiotherapy practice, patient expectations, and many funding models. Constructivist philosophy posits that having experienced an adverse health state necessarily alters one’s sense of what is ’normal’. Traditional understandings of recovery also suggest that reaching a pre-injury status is the best outcome that can be achieved, but does not account for phenomena such as ’survivor pride’ in which people may perceive better-than-previous quality of life after having experienced a traumatic event. Using satisfaction or happiness as an outcome allows greater flexibility in treatment targets and prevents clinicians from working towards otherwise unreachable goals. While these issues are pertinent to any health condition, it is especially relevant for chronic conditions.

**Target Population/Population cible:** This session will be relevant to clinicians, administrators, and policy makers who track outcomes or make discharge decisions following physical rehabilitation.

**Supporting Evidence/Résumé des faits:** Recent research from Walton and colleagues has suggested that recovery, as defined by a return to pre-morbid condition, is unreachable, unmeasurable, and not necessarily what patients desire. Based on theories including Maslow’s theory of Self-Actualization, Higgins’ Self- Discrepency Theory, and Ryan and Deci’s theories on Happiness and Human Potential, Walton (2011) has argued that satisfaction or happiness function as more appropriate rehabilitation outcomes. A subsequent set of focus groups, written reflections and one-on-one interviews with patients and clinicians (under review) has suggested that desirable outcomes for patients go beyond physical capacity towards satisfying re- engagement in valued life roles and with perceived potential for future achievement. There are a variety of published tools for measuring happiness, satisfaction, and quality of life that can serve as reasonable proxies for acceptable outcomes. A new tool will also be presented that has shown promise for use as a measure of personal satisfaction with current and future health status.

**Session Format/formule de la session:** This will be an interactive session in which the facilitator will guide participants through a process of critical reflection on the nature of recovery and how that affects their current practice. Over a one-hour time slot, roughly 20 minutes will serve as an introduction through more didactic presentation of new and novel information in the field. Subsequently, participants will consider methods for evaluating recovery and will reach consensus on appropriate tools. Discussion on tools that are available to all clinicians will ensue, and discussion on basic understanding of measurement properties will help participants critically appraise new tools. A 5 to 10-minute window for summary and wrap-up will be reserved for the end of the session.

**Conclusions & implications:** Anecdotal evidence and clinical experience suggest that clinicians are often challenged to identify reasonable treatment targets and goals, especially in chronic conditions. Much of this challenge can be linked to treatment goals that require a return to a previous state, which is arguably impossible. This session will re-frame clinician’s understandings of reasonable and attainable treatment targets, which will necessarily influence treatment targets. Participants will be encouraged to think critically about the tools they use to operationalize outcomes, which will serve them now and in the future.

**Keywords/Mots clés:** Recovery, measurement, response shift, satisfaction, illness representations, quality of life

# Abstract: P021 – Are we delaying patient recovery by not identifying central sensitization pain?

**Published:** 2013-05

**Overview & Objectives/Résumé & objectifs:** 1) Discuss and demonstrate the subjective and objective presentations of each pain classification including Central Sensitization Pain (CSP), Peripheral Neurogenic Pain (PNP), and Nociceptive pain (NP) using practical demonstrations 2) Discuss the optimum management approaches for each classification category but with focus on Central Sensitization Pain

**Relevance/Pertinence:** Central sensitization pain (CSP) is due to abnormal processing of the cerebral cortex. It is essential for PTs to identify CSP as management focusing on treating specific anatomical structures is likely to be futile. In fact, treatments such as pain relieving modalities, pain medication, specific manual therapy, and specific localized exercises, may all exacerbate the cerebral cortex’s focus on pain and even further contribute to chronicity.

**Target Population/Population cible:** All PTs involved in the evaluation and management of persistent spinal pain

**Supporting Evidence/Résumé des faits:** The most recent 2012 multi-center study found that PTs were able to identify a cluster of symptoms to identify Central Sensitization Pain (CSP) with high levels of classification accuracy (sensitivity 92%, specificity 98%). Pattern recognition of this cluster of signs and symptoms may help PTs identify a dominance of CSP in patients with spinal pain and help better guide management strategies.

**Session Format/formule de la session:** Lecture format discussing the theory and case studies with live practical demonstrations of specific evaluation procedures.

**Conclusions & implications:** As pain science advances, the ability for PTs to recognise and classify patients with Central Sensitization Pain (CSP) early in the assessment process may be of great benefit. Early and accurate identification of CSP may help reduce inappropriate, ineffective and potentially harmful management strategies focusing on patho-anatomy and biomechanics. Timely application of more appropriate treatment strategies specific to CSP may help reduce the risk of chronicity in this subgroup of patient population.

**Keywords/Mots clés:** central, sensitization, pain, diagnosis, classification, management, chronic

# Abstract: P023 – Viscosupplementation: Hype or hope?

**Published:** 2013-05

**Authors:** S Kerslake

**Affiliations:** Banff Sport Medicine, Banff Alberta Canada; Department of Physical Therapy, University of Alberta, Edmonton, AB

**Overview & Objectives/Résumé & objectifs:** At the end of this session, participants will be able to:

1. Discuss the biological properties of viscosupplementation for the treatment of osteoarthritis
2. Evaluate the effectiveness of viscosupplementation for various stages of osteoarthritis
3. Apply evidence-based viscosupplementation knowledge in treatment discussions with patients

**Relevance/Pertinence:** Osteoarthritis is one of the most common reasons that patients seek physiotherapy care and current projections indicate that this disease will affect one in every two people by 2030. Viscosupplementation is emerging a mainstay of treatment for all stages of osteoarthritis. Knowledge about the biology and effectiveness of these products is important for therapists involved in the rehabilitation of this patient population. Protection of hyaline cartilage, reduced pain and improved function are some of the purported benefits of these products but does the research support these claims? In addition to questions about the effectiveness of this treatment modality, therapists must also be aware of restrictions and limitations when rehabilitating patients following viscosupplementation injections.

**Target Population/Population cible:** This session will be of interest to a broad range of therapists, in particular those working in orthopedic and other musculoskeletal practice areas involved in treating patients with osteoarthritis.

**Supporting Evidence/Résumé des faits:** Viscosupplementation injections for the treatment of osteoarthritis have demonstrated beneficial effects on pain and function. Recent studies examining the effect of repeated injections have shown improved hyaline cartilage depth and reduced symptoms resulting in measureable quality of life benefits. Bench research has demonstrated the ability of viscosupplementation products to stimulate synovial fluid production, reduce synovitis and decrease glycosaminoglycan turnover potentially leading to reduced hyaline cartilage degeneration. To date a significant volume of viscosupplementation research and treatment has focused on advanced osteoarthritis outcomes with less attention paid to earlier stages of the disease. In the past few years, viscosupplementation has been used earlier in the disease process including post-operatively. The addition of these treatment applications has implications for physiotherapists treating and advising patients with chondral cartilage pathology and warrants evaluation and discussion.

**Session Format/formule de la session:** This session will be a 45-minute lecture format, with 10–15 minutes for participant discussion of cases and questions.

**Conclusions & implications:** Initial research evidence has demonstrated that viscosupplementation has the potential to dramatically alter the disease course and treatments used in the treatment of osteoarthritis. Based on this developing research, viscosupplementation is emerging as a regular component of osteoarthritis care. Physiotherapy continues to be one of the main pillars of osteoarthritis treatment however the increasingly frequent use of viscosupplementation has implications for PT practice that warrant attention and evaluation.

**Keywords/Mots clés:** Osteoarthritis, Viscosupplementation

# Abstract: P024 – Facilitating Return to Work after Stroke: Creation of a Web-based Toolkit

**Published:** 2013-05

**Authors:** P Gilmore, J Barry, J Blanchard, S Howson, M Scott, M Solomon

**Affiliations:** * London Health Sciences Centre, Southwestern Ontario Stroke Network, London, ON; † Pursuit Health Management, London, ON; ‡ London, ON; § St. Joseph’s Health Centre, Parkwood Hospital, London, ON; ¶ International Neurological Physical Therapy Association, Meaford, ON

**Overview & Objectives/Résumé & objectifs:** The session will focus on an innovative project where a web- based toolkit of resources has been developed based on best practice to assist stroke survivors and health care professionals/providers navigate the process of return to work. This web-based toolkit has been designed as a self management tool navigating users to various resources based on their individual work situation. The presentation will highlight the literature for return to work after stroke as well as provide a guided tour of the web-based toolkit and how to use the resources with stroke survivors who wish to return to work after stroke. Web-based resources that will be reviewed include a self assessment guide designed to help focus an individual’s recovery efforts and inform him/her about his/her current ability to return to work, questions to ask employers and insurers, government financial supports, organizations providing return to work services and volunteering after stroke. At the end of this session, participants will be able to: 1) Discuss recent literature and best practices for return to work after stroke; 2) Describe barriers and enablers to returning to work after stroke; and 3) Access and utilize a web-based toolkit of resources to assist clients with return to work after stroke.

**Relevance/Pertinence:** Assisting clients to prepare to return to work is an important aspect of physiotherapeutic intervention and practice. The establishment of a highly relevant and significant goal can increase a client’s drive and likelihood of participation, mental focus and practice outside of therapy time. Current literature on neuroplasticity has identified these factors as key to maximizing brain recovery. Therefore selecting a relevant and motivating goal can be critical for the rehabilitation program. For a growing proportion of stroke survivors, return to work is a focus or goal that best fits those criteria.

**Target Population/Population cible:** Physiotherapists working with stroke survivors across the continuum of care (acute, inpatient rehabilitation, community) interested in assisting their clients with return to work. As well, the presentation is applicable for physiotherapists working with any person with a chronic disease who is interested in exploring return to work.

**Supporting Evidence/Résumé des faits:** Approximately, 26% of stroke survivors are between the ages of 45- 65, 10% are under the age of 50 and in the prime of their working life. Research indicates that return to work rates after stroke are as low as 7%. Employment is one of the most important social roles that a person fulfills and not working has negative impacts on one’s overall quality of life, health, finances, social isolation and self-efficacy. The Southwestern Ontario Stroke Network completed community engagement forums with stroke survivors, their loved ones and community service providers to determine barriers to living fully in the community after stroke. One of the priorities identified in the forums and documented in the report “Pathways for People with Stroke to Live Fully in the Community” was the need for return to work services as these services are often neglected in rehabilitation after stroke. Evidence indicates that stroke survivors should be evaluated for their potential of returning to work and receive support from knowledgeable professionals regarding return to work as soon as possible after stroke.

**Session Format/formule de la session:** This session will be a lecture format with the demonstration of a web-based toolkit and opportunity for participant discussion regarding their experiences with assisting clients with return to work.

**Conclusions & implications:** Return to work is often neglected in stroke survivors’ rehabilitation. Stroke survivors should be evaluated for their potential of returning to work and receive support from knowledgeable professionals regarding return to work as soon as possible after stroke. The newly created web-based toolkit of return to work resources will assist stroke survivors, loved ones/family and health care professionals/providers with the complex process of return to work.

**Keywords/Mots clés:** Return to Work, Stroke

# Abstract: P025 – From Best Practice Model to Program Reality: How to Succeed in Implementing a Best Practice Model of Self - Management for Chronic Pain Clients in your Community

**Published:** 2013-05

**Authors:** M Beaton, I Zwerling

**Affiliations:** South Shore Health, Bridgewater, NS

**Overview & Objectives/Résumé & objectifs:** Learning Objectives and Session Content:

This session will give a brief history of the specific planning and start-up steps taken to implement the Chronic Pain Self-Management Program as a new client service. Critical success factors that were instrumental to achieving this new practice reality are identified. Application to other practice realities will be discussed.

At the end of this session participants will be able to:

1. Identify given critical success factors in actually introducing and implementing the (Best Practice) Stanford Model known as the Chronic Pain Self-Management Program as a new practice;
2. Evaluate how these identified critical success factors apply to their own practice realities;
3. Identify and evaluate the actual feasibility of implementing this (Best Practice) Model within their own practice realities.

**Relevance/Pertinence:** All health professions today are experiencing a strong political and funding push towards using best practice evidence to deliver quality programs to their clients. Physiotherapists and other allied health professionals are primary health personnel who occupy critical roles as direct client educators and supporters of optimal client independence. It is vital that physiotherapists and leaders of the profession be educated in both proven best practice methods and programs that work so that they may successfully incorporate these into their own practice realities.

**Target Population/Population cible:** This session will be of interest to a broad range of professionals including clinicians, managers, professional leaders, educators and researchers interested in the implementation of best practices.

**Supporting Evidence/Résumé des faits:** There is an almost 20year academic history of supporting scientific evidence for the Chronic Disease Self-Management Program designed at Stanford University by Kate Lorig PhD (Gordon & Galloway, 2008). The Chronic Pain Self- Management Program is designed by Sandra LeFort PhD -of Memorial University- under the approval of Kate Lorig and operates under the same licence (LeFort et al., 1998). As such, these programs are accepted as valid, reliable and effective in their specific content and process. In Canada, the Chronic Pain Self-Management Program is presently being delivered in Quebec, Ontario, Alberta, British Columbia, and Nova Scotia. It is also being delivered in many parts of the United States, Denmark and Australia.

**Session Format/formule de la session:** This session will be short lecture format with power point, followed by focused small group discussion of content and applicability and open discussion of feasibility issues of interest to the audience.

**Conclusions & implications:** Actually incorporating best practice knowledge into real life practice requires identifying critical success factors that transform awareness into reality and the keen judgment to properly assess one’s own environment for program readiness. Success requires that all factors be properly considered.

**Keywords/Mots clés:** innovative practice

# Abstract: D002 – Diagnostic Imaging and Low Back Pain: Just Say No

**Published:** 2013-05

**Authors:** C Cook

**Affiliations:** Walsh University, Division of Physical Therapy, North Canton, Ohio, US

**Objectives/Objectifs:** Upon completion of this session, participants will be able to:

1. Recognize and identify the limitations of use of imaging for diagnosis of LBP
2. Compare and contrast imaging methods with clinical methods
3. Discuss how use of images and the identification of a “label” have affected prognoses of patients
4. Compare and contrast the recommendations of imaging for LBP guidelines.

**Background/Contexte:** Despite the fact that early use of diagnostic imaging for routine conditions of low back pain (specifically, acute low back pain) is not recommend in any of the 39 guidelines for low back pain, diagnostic imaging is still frequently used; even methods such as radiographic imaging.1 Evidence has suggested that use of early imaging may actually be detrimental to recovery from low back pain.

**Relevance/Pertinence:** Physiotherapists, worldwide, have increased their presence as first line providers of musculoskeletal conditions. In some countries as well as in military based health environments, physiotherapists can order imaging. Ordering appropriate imaging is a specific skill set that any first line provider should know. The ability to discuss why imaging is not needed and “why” is also an imperative skill and a purpose of this presentation.

**Target Population/Population cible:** Physiotherapist and allied health care professionals. All experience levels.

**Supporting Evidence/Résumé des faits:** Diagnostic imaging is indicated for patients with low back pain only if they have severe progressive neurologic deficits or signs or symptoms that suggest a serious or specific underlying condition. In other patients, evidence indicates that routine imaging is not associated with clinically meaningful benefits and can actually lead to harmful results. Addressing inefficiencies in diagnostic testing could minimize potential harms to patients and have a large effect on use of resources by reducing both direct and downstream costs.2

**Session Format/formule de la session:** This presentation will involve a lecture-based format with time set aside for discussion and questions from the audience.

**Keywords/Mots clés:** Diagnostic imaging, low back pain, X-Ray, MRI

# Abstract: D007 – Non-Steroidal Anti-Inflammatory Drugs – Indications, Evidence and Relevance for Physiotherapists

**Published:** 2013-05

**Authors:** S Kerslake

**Affiliations:** Banff Sport Medicine, Banff Alberta Canada, Department of Physical Therapy, University of Alberta, Edmonton, AB

**Objectives/Objectifs:** On completion of this session participants will be able to:

1. Identify the current NSAID-specific recommendations and indications for musculoskeletal conditions and injuries
2. Evaluate the research evidence for NSAID use in orthopaedic physical therapy
3. Appreciate the risks and benefits of recommending NSAIDs as an adjunct to physiotherapy treatment

**Background/Contexte:** Knowledge of the pathophysiology and healing processes of orthopedic conditions and injuries is a crucial consideration for clinicians prior to recommending NSAIDs. For example, research has demonstrated that conditions such as Achilles tendonitis are primarily degenerative as opposed to inflammatory processes, although tendonitis was treated as an inflammatory condition for a substantial length of time. Also, the likelihood of adverse effects from NSAIDs are not insignificant and interaction with other medications can cause profound side effects, therefore a sound knowledge of the risks and benefits of this medication class is required. A substantial challenge for physiotherapists in practice is accessing and synthesizing the research in this vast and evolving field.

**Relevance/Pertinence:** Non-steroidal anti-inflammatory drugs are frequently recommended or prescribed for musculoskeletal injuries by orthopaedic health care providers. Physiotherapy- specific research has demonstrated that one third of orthopaedic therapists regularly recommend NSAIDs to their patients. Recommendation of NSAIDs stems from the theory that many musculoskeletal injuries and conditions have a significant inflammatory cause. In terms of healing, the inflammatory process is essential and therefore decreasing inflammation too substantially may result in adverse outcomes.

**Target Population/Population cible:** This session will be of greatest interest to clinicians assessing and treating orthopaedic injuries in acute, surgical and rehabilitation settings. Content will be applicable for novice through to experienced clinicians. Content summaries for indications, evidence and applications of NSAIDs will have direct clinical relevance.

**Supporting Evidence/Résumé des faits:** Recent systematic reviews and randomised clinical trials have demonstrated a higher than previously appreciated risk of adverse events associated with NSAIDs. Although adverse event rates vary by age group, all age groups are affected by these cardiovascular, gastrointestinal, musculoskeletal and immunological side effects. The frequent prescription and widespread over-the-counter availability of NSAIDs also appears to contribute to the belief that the effects of this class of medications are at worst benign and at best remarkably positive. Physiotherapists regularly recommend NSAIDs to their patients and research has shown therapists understand the positive effects but may not appreciate the negative effects of these medications. In practice the challenges for physiotherapists include: accurately determining which patients and conditions will benefit from NSAIDs, how substantial the benefits of NSAIDs are in musculoskeletal care, and which patients are at greatest risk of adverse events.

**Session Format/formule de la session:** Lecture for 45 minutes including case-study discussions among audience members, followed by 10–15 minute question and answer session.

**Keywords/Mots clés:** NSAIDs, Indications, Musculoskeletal, Adverse Effects

# Abstract: D008 – Applying best practice in exercise prescription for special populations: A panel discussion

**Published:** 2013-05

**Authors:** DM Connelly, R Debigaré, J Harris, TL Parsons

**Affiliations:** * Associate Professor, School of Physical Therapy, The University of Western Ontario, London ON; † Associate Professor, Rehabilitation Department, Medicine Faculty, Université Laval, QC; ‡ University of Ottawa Heart Institute, Ottawa, ON; § Assistant Professor, Queen’s University, Kingston, ON

**Objectives/Objectifs:**

1. To outline the type of patient that would benefit from rehabilitation in four specific patient populations (COPD, cardiovascular disease, chronic kidney disease and older adults)
2. To increase awareness of current best practice guidelines and relevant outcome measures in these practice areas
3. To integrate practical considerations when applying this knowledge to the four specific patient populations in a variety of practice settings
4. To reviewappropriate exercise prescription and types of exercise used with these four patient populations

**Background/Contexte:** Exercise & Chronic Kidney Disease (CKD) - Canadians living with CKD experience significant multi-system consequences of the disease which threaten their quality of life and independence. Physiotherapy is clearly indicated in the management of these consequences, yet services are not equally accessible to all Canadians with CKD. One potential barrier is the clinician’s knowledge, skills, and attitudes related to the competent delivery of PT service within a CKD context. The information provided in this session will focus on: a) the state of the evidence in support of exercise training in chronic kidney disease; and b) an overview of the issues and indications across the spectrum of CKD (i.e., pre-end-stage, end-stage, and kidney transplant).

Exercise &Cardiovascular Disease (CVD)- Exercise is well established as one of the most important treatments for cardiovascular disease. Evidence continues to mount demonstrating that exercise will not only increase the life span for those with CVD, but also improve the quality of life for people living with symptomatic heart disease (Warburton et al;, 2010). While physiotherapists have long been advocates of promoting physical activity and exercise, they are also keenly aware that there are different kinds and intensities of exercise appropriate for different populations. The goal of the exercise prescription for the cardiac client is to provide both a safe program of exercise and one that is effective in reducing risk and optimizing functional capacity. The Canadian Guidelines for Cardiac Rehabilitation and Cardiovascular Disease Prevention (CACR, 3rd Edition) recommend that adults should accumulate a minimum of thirty minutes and up to sixty minutes of aerobic type activity most days of the week and that participation in exercise and physical activity programs should be incorporated into a lifelong program of heart healthy living. Released in 2011, the harmonization of guidelines for the prevention and treatment of cardiovascular disease: The C-Change Initiative, extends this recommendation by adding that it is also beneficial to add muscle and bone strengthening activities to the exercise program (Tobe et al., 2011). This presentation will give a brief overview of the evidence supporting exercise in the cardiac client, the guidelines that are used to develop appropriate exercise prescriptions and a discussion on the transition of cardiac clients from hospital/clinic-based exercise to a lifelong habit of exercise in their own communities.

Exercise & COPD - Chronic obstructive pulmonary disease (COPD) is a progressive and irreversible lung disease mainly characterized by dyspnea and cough. Exercise intolerance is present in this population, however, respiratory dysfunction is not the sole contributing factor. Indeed, peripheral muscle dysfunction is present and increases in severity as the lung disease progresses. Changes in fibre type composition, increased glycolytic metabolism and loss in muscle mass are linked to poor endurance, muscle weakness, low quality of life and increased mortality. Exercise training is the main component of pulmonary rehabilitation program. Resistance- as well as endurance-type training programs have been effective in modulating exercise and muscular parameters. Demonstration of its effectiveness emphasizes the adoption of an aggressive approach in enhancing the use of exercise training in this population. Unfortunately, modest accessibility to pulmonary rehabilitation is a strong barrier for patients and clinicians.

Exercise & Older Adults - The purpose of this presentation is to provide an overview of the age-related changes to the musculoskeletal, neuromuscular and cardiorespiratory systems and the implications of these changes as they relate to exercise mode, prescription and monitoring in healthy, older adults. The new Canadian Physical Activity Guidelines for Older Adultsadvocates that adults aged 65 years and older should accumulate at least 150 minutes of moderate- to vigorous-intensity aerobic physical activity per week, in bouts of 10 minutes or more. As well, adding muscle and bone strengthening activities using major muscle groups, at least two days per week, are suggested. Those with poor mobility are advised to engage in physical activities to enhance and prevent falls. And finally, greater health benefits are provided with more physical activity. Physical therapists, who understand the importance of exercise and physical activity in older adult populations (Chodzko-Zajko et al., 2009), can play a major role in assisting older adults with interpreting these recommendations for physical activity and advising them about any modifications to exercise needed to suit their level of function, interests, goals and existing health conditions.

**Relevance/Pertinence:** The escalating prevalence of chronic disease and the impact of an aging population have resulted in the increased complexity of patients requiring physiotherapy intervention in all practice settings. Patients are increasingly presenting with a wide range of co-morbidities that have the potential to significantly affect and impact physiotherapy practice. This interactive session is intended to provide physiotherapists with the current best practices regarding the role of exercise in the management of older adults, and patients with three specific chronic diseases [renal, cardiovascular and chronic obstructive pulmonary disease (COPD)]. Moreover, the information presented will provide an opportunity for physiotherapists to consider the different exercise prescriptions and types of exercise that can be utilized with these populations. Lastly, via a moderator-led discussion with this panel of presenters, this session will address practical considerations in applying this knowledge to various practice settings.

**Target Population/Population cible:** Target audience is physiotherapists with a basic understanding of the concepts and terms of exercise prescription and a basic understanding of the terms associated with our four patient populations (older adults, COPD, cardiovascular disease and renal disease).

**Supporting Evidence/Résumé des faits:** The information presented in this session will be based upon the currently available best practice guidelines and recommendations for exercise prescription and rehabilitation in each patient population. These include but are not limited to:

1. Chodzko-Zajko WJ, Proctor DN, Fiatarone Singh MA, Minson CT, Nigg CR, Salem GJ, Skinner JS. Exercise and Physical Activity for Older Adults. Medicine & Science in Sports & Exercise 2009;41(7):1510-1530.
2. Canadian Society for Exercise Physiology. Canadian Physical Activity Guidelines for Older Adults – 65 Years & Older. Available at: http://www.csep.ca/CMFiles/Guidelines/CSEP-InfoSheets-older%20adults-ENG.pdf)
3. Optimizingpulmonaryrehabilitation in chronic obstructive pulmonarydisease – practical issues: A Canadian Thoracic Society Clinical Practice Guideline. Can Respir J. 2010 Jul-Aug; 17(4): 159–168.
4. Heiwe, S., & Jacobson, S. H. (2011). Exercise training for adults with chronic kidney disease. Cochrane Database of Systemic Reviews, (10), CD003236.
5. Stone JA, Arthur HM, Suskin NG editors. The Canadian Guidelines for CardiacRehabilitation and CardiovascularDisease Prevention.3rded. Winnipeg (MB): Canadian Association of CardiacRehabilitation 2009.
6. American Thoracic Society/EuropeanRespiratory Society Statement on PulmonaryRehabilitation. Am J RespirCrit Care Med. 2006 Jun 15;173(12):1390-413.

The panel will offer their opinions on the practical application and integration of this knowledge through a moderator led discussion period.

**Session Format/formule de la session:** The first hour will be lecture format and the second hour will be a moderator-led discussion with the panel.

**Keywords/Mots clés:** exercise, COPD, older adults, cardiovascular disease, renal disease, best practice

# Abstract: D010 – Provoked Vestibulodynia (PVD): Defining the dysfunction and providing effective management

**Published:** 2013-05

**Authors:** MJ Lord, M Morin, C Pukal, S Thibault-Gagnon

**Objectives/Objectifs:** At the end of this session, participants will be able to discuss the current state of evidence defining muscle dysfunction in women with provoked vestibulodynia (PVD) and identify priorities for future directions, evaluate the effectiveness of a multidisciplinary approach to treating women with PVD, and provide evidenced based rationale for current best practice strategies when treating women with PVD.

**Background/Contexte:** PVD is the most common form of dyspareunia (recurrent pain during sexual intercourse) in pre-menopausal women and is commonly treated by pelvic health physiotherapists. PVD affects 12–15% of women and has serious negative effects on sexual functioning, psychological well-being, and quality of life (Arnold et al., 2001). PVD is characterized by severe burning pain felt at the vulvar vestibule when pressure is applied to this area, resulting in painful vaginal penetration during sexual intercourse as well as non-sexual activities such as tampon insertion and gynaecological examinations (Bergeron et al., 2001). Although the etiology of PVD is largely unknown and there remains controversy in the conceptualization of PVD, clinicians and researchers agree that it is a multidimensional condition in which biological, psychological, and social influences play a role, and consideration of all associated factors is necessary for proper management of the condition. It has been suggested that an integrated model taking into consideration the biomedical, cognitive, affective, behavioural, and interpersonal factors associated with sexual impairment be adopted in the treatment of PVD (Bergeron et al., 2009).The fragmentation of scientists into specific disciplines, ongoing classification issues, and lack of sound research designs all impede progress concerning the identification of etiological factors and the development of tailored interventions for women with PVD. In order to progress in the management of a multifactorial condition such as PVD, our research methodologies require expansion and the involvement of cross-discipline approaches.

**Relevance/Pertinence:** The implementation of a biopsychosocial model in the assessment of women with PVD can help establish a successful management program for women with PVD. Each panelist has extensive experience in identifying dysfunctions associated with PVD and implementing treatment strategies to rehabilitate women with PVD. Each panelist contributes to the biopsychosocial model in a unique and collaborative way. The combination of panelists will create a comprehensive understanding of a multifactorial problem that will directly translate into improved clinical awareness during the assessment phase and will lead to improved clinical outcomes. In addition, future directions for research will be identified and discussed.

**Target Population/Population cible:** The target audience for this session would be clinicians, educators, and researchers working in the area female pelvic health. The session will offer a multifaceted overview of physical dysfunctions and psychological factors associated with PVD, as well as the current physiotherapy management of PVD. The session content will be appropriate for those who have little background knowledge on PVD as well as those with greater knowledge as the speakers will be presenting on the latest understanding and management strategies for women with PVD.

**Supporting Evidence/Résumé des faits:** The etiology of PVD is unknown, many factors are suspected to play a role. The pelvic floor muscles have been implicated and pelvic floor rehabilitation appears to be a beneficial treatment (Morin and Bergeron, 2009). However, there is a lack of valid and reliable methods for assessing pelvic floor muscle function (Bergeron et al., 2011). Pelvic floor USI can be used to assess pelvic floor muscle (PFM) morphology and function, and in recent years it has gained popularity amongst physiotherapists in the research and clinical settings. The use of ultrasound imaging in combination with dynamometry and electromyography allows for a comprehensive evaluation of muscle function that can help identify dysfunction and ultimately direct treatment decisions.

Pelvic floor rehabilitation aims to increase awareness and proprioception of the pelvic floor muscles, improve muscle relaxation, normalize muscles tone, increase extensibility of the vaginal tissues, desensitize the painful area, and decrease fear of vaginal penetration (Bergeron and Lord, 2002). Pelvic floor rehabilitation achieves this through the use of education, biofeedback, manual therapy, insertional techniques, as well as electrotherapy modalities. Pelvic floor muscle exercises are the foundation of pelvic floor rehabilitation and are often prescribed to improve muscle control and to promote relaxation. Studies have demonstrated the effectiveness of comprehensive pelvic floor rehabilitation programs (Bergeron et al., 2001; Goldfinger et al.,

2009) and multidisciplinary approaches (Spoelstra et al., 2011; Backman et al., 2008) for the treatment of PVD. A comprehensive program has been shown to be effective in significantly reducing pain associated with intercourse and gynecological examinations, improving sexual function and decreasing pain cognitions, increasing vestibular pain thresholds, normalizing pelvic floor muscle tone and activity, improving relaxation capacity, as well as decreasing pelvic floor muscle contractile responses when a painful pressure stimulus is applied to the vestibule (Bergeron et al., 2002; Goldfinger et al., 2009; Gentilcore-Saulnier et al., 2010).

Physical dysfunction is just one aspect of PVD, it is equally important to consider the cognitive, emotional, behavioral, and interpersonal factors that are associated with PVD when developing treatment programs. Currently, a multidimensional viewpoint in which biological, psychological, and social factors are all seen as fundamental to understanding and treating PVD is accepted by most researchers and clinicians (Goldstein & Burrows, 2008; Weijmar et al., 2005). Cognitive-behavioural therapy (CBT) is a commonly used treatment option for the management of various chronic pain conditions, including PVD. Success rates ranging from 39 to 86% have been reported in several studies in which sex therapy and pain management were combined in a group format (Abramov et al., 1994; Bergeron et al., 2001; Bergeron et al., 2008; Weijmar-Schultz et al.,

1996). Results from a randomized trial indicate that although the effects of group CBT may take longer to present, it is as effective as vestibulectomy in reducing intercourse pain intensity (Bergeron et al., 2001; Bergeron et al., 2008). These results indicate that a directed treatment approach including both cognitive and behavioural techniques for pain management may yield better outcomes and greater satisfaction than a less directive approach.

Overall, there is a need to investigate a variety of treatment options for PVD. There is still much that needs to be investigated in terms of the role of adherence and other factors in predicting and explaining the process of improvements. There is also a need to develop methods for objective quantification of PFM functioning in women with PVD in order to reliably measure functional impairments at baseline and investigate change in PFM components following treatment.

**Session Format/formule de la session:** Four panelists will present on the management of PVD. Panelist 1 and

2 will report on the most current understanding of muscle morphology and dysfunction in women with PVD based on the evaluation using ultrasound imaging, dynamometry, and EMG. The third panelist will speak to psychological factors and non-medical treatments associated with PVD, as well as highlight interesting findings from studies on pelvic floor rehabilitation in women with PVD and a randomized study comparing pelvic floor rehabilitation with cognitive behavioral therapy. The fourth panelist will examine the current physiotherapy best practice strategies for treating women with PVD. All four will emphasize where the literature stands now and make recommendations for future directions.

**Keywords/Mots clés:** Provoked vestibulodynia, pelvic floor muscle dysfunction, pelvic floor muscle re- education

# Abstract: D011 – Lumbopelvic Motor Control: Moving Evidence Into Action

**Published:** 2013-05

**Authors:** D Teyhen

**Affiliations:** US Army-Baylor University, Waco, TX

**Objectives/Objectifs:**

1. Identify the sub group of patients likely to succeed with motor control training exercises within the context of a comprehensive classifications system for patients with LBP.
2. Identify manual therapy and therapeutic exercise treatments and progression strategies to enhance motor control and clinical outcomes.
3. Understand the importance of translating evidence into clinical practice
4. At the completion of this presentation, participants will understand the utilization of appropriate diagnostic imaging modalities to assist with motor control rehabilitation.

**Background/Contexte:** Although the management of low back pain continues to be considered an “epidemic” in healthcare systems around the world, physical therapists offer many evidence-based and cost- effective solutions that result in optimal outcomes. This presentation will help translate the current evidence to facilitate enhanced clinical decision-making with practical applications across the spectrum of patients with low back pain. Specifically, this presentation will focus on the latest evidence regarding motor control training strategies and functional progression to optimize outcomes of care.

**Relevance/Pertinence:** Persistent lumbopelvic pain is a recalcitrant condition to standard medical approaches to treatment. It is important that physiotherapists can recognize those patients at risk of poor recovery and/or at risk of non-responsiveness to treatment. The research evidence also suggests that some patients, especially those with poor outcomes demonstrate a complex clinical picture that will likely require an integrated approach to management from several providers. The physiotherapist, by virtue of our assessment skills, will play a unique and important role in the integration of patient care.

**Target Population/Population cible:** This presentation is aimed at graduate physiotherapists who are involved in the management of patients with WAD.

**Supporting Evidence/Résumé des faits:** This presentation will provide physiotherapists with an introduction to the skills required to assess the patient with lumbopelvic pain, taking into account all aspects of the condition both physical and psychological. This lays the foundation for enhanced management and improved patient outcomes.

1. Childs JD, Teyhen DS, Benedict TM, et al. Effects of sit-up training versus core stabilization exercises on sit-up performance. Med Sci Sports Exerc. 2009;41:2072-2083.
2. Childs JD, Teyhen DS, Casey PR, et al. Effects of traditional sit-up training versus core stabilization exercises on short-term musculoskeletal injuries in US Army soldiers: a cluster randomized trial. Phys Ther. 2010;90:1404-1412.
3. Fritz JM, Koppenhaver SL, Kawchuk GN, et al. Preliminary investigation of the mechanisms underlying the effects of manipulation: exploration of a multivariate model including spinal stiffness, multifidus recruitment, and clinical findings. Spine (Phila Pa 1976). 2011;36:1772-1781.
4. George SZ, Childs JD, Teyhen DS, et al. Brief psychosocial education, not core stabilization, reduced incidence of low back pain: results from the Prevention of Low Back Pain in the Military (POLM) cluster randomized trial. BMC Med. 2011;9:128.
5. George SZ, Childs JD, Teyhen DS, et al. Predictors of occurrence and severity of first time low back pain episodes: findings from a military inception cohort. PLoS One. 2012;7:e30597.
6. George SZ, Teyhen DS, Wu SS, et al. Psychosocial education improves low back pain beliefs: results from a cluster randomized clinical trial (NCT00373009) in a primary prevention setting. Eur Spine J. 2009;18:1050-1058.
7. Koppenhaver SL, Fritz JM, Hebert JJ, et al. Association between changes in abdominal and lumbar multifidus muscle thickness and clinical improvement after spinal manipulation. J Orthop Sports Phys Ther. 2011;41:389-399.
8. Koppenhaver SL, Fritz JM, Hebert JJ, et al. Association between history and physical examination factors and change in lumbar multifidus muscle thickness after spinal manipulation in patients with low back pain. J Electromyogr Kinesiol. 2012;22:724-731.
9. Teyhen D, Miltenberger C, Deiters H, et al. The use of ultrasound imaging of the abdominal drawing-in maneuver in subjects with low back pain. J Orthop Sports Phys Ther. 2005;35:346-355.
10. Teyhen DS. Rehabilitative ultrasound imaging for assessment and treatment of musculoskeletal conditions. Man Ther. 2010;16:44-45.
11. Teyhen DS, Bluemle LN, Dolbeer JA, et al. Changes in lateral abdominal muscle thickness during the abdominal drawing-in maneuver in those with lumbopelvic pain. J Orthop Sports Phys Ther. 2009;39:791- 798.
12. Teyhen DS, Childs JD, Dugan JL, et al. Effect of Two Different Exercise Regimens on Trunk Muscle Morphometry and Endurance in Soldiers in Training. Phys Ther. 2012.
13. Teyhen DS, Childs JD, Stokes MJ, et al. Abdominal and lumbar multifidus muscle size and symmetry at rest and during contracted States. Normative reference ranges. J Ultrasound Med. 2012;31:1099-1110.
14. Teyhen DS, Flynn TW, Bovik AC, Abraham LD. A new technique for digital fluoroscopic video assessment of sagittal plane lumbar spine motion. Spine. 2005;30:E406-413.
15. Teyhen DS, Flynn TW, Childs JD, Abraham LD. Arthrokinematics in a subgroup of patients likely to benefit from a lumbar stabilization exercise program. Phys Ther. 2007;87:313-325.
16. Teyhen DS, Flynn TW, Childs JD, et al. Fluoroscopic video to identify aberrant lumbar motion. Spine. 2007;32:E220-229.
17. Teyhen DS, Rieger JL, Westrick RB, et al. Changes in deep abdominal muscle thickness during common trunk-strengthening exercises using ultrasound imaging. J Orthop Sports Phys Ther. 2008;38:596-605.
18. Teyhen DS, Williamson JN, Carlson NH, et al. Ultrasound Characteristics of the Deep Abdominal Muscles During the Active Straight Leg Raise Test. Arch Phys Med Rehabil. 2009;90:761-767

**Session Format/formule de la session:** One hour session: 45 mins lecture with 15 mins of questions from audience

**Keywords/Mots clés:** Lumbopelvic Pain; Motor Control; Low Back Pain; Stability; Exercises

# Abstract: A014 – Ethics Content in Physical Therapy University Programs in Canada

**Published:** 2013-05

**Authors:** M Laliberté, A Hudon, V Sonier, V Badro, M Hunt, DE Feldman

**Affiliations:** Programme de physiothérapie, École de réadaptation, Faculté de Médecine, Université de Montréal, Montréal, QC et School of Physical and Occupational Therapy, McGill University, Montréal, QC

**Purpose & Objectives / Intention & objectifs:** Ethics training for students in health care professions is an essential component of the preparation of future clinicians. Currently, there is a lack of information on the content and format of ethics training for physiotherapy students, and variations between programs. The objective of our study is to explore ethics teaching in Canadian university physiotherapy programs. The rationale is that by knowing what is actually being taught, we will be better placed to assess and improve or adapt ethics training in line with current needs.

**Relevance/Pertinence:** Moral development is essential for becoming a conscientious physiotherapist. For aspiring physiotherapists it involves both a solid clinical training but also opportunities to develop skills necessary to address and resolve clinical moral challenges.

**Materials & Methods/Matériel & méthods:** This inquiry includes two components: We reviewed Canadian university physiotherapy program websites followed by a content-analysis of ethics course syllabi; and are currently surveying those responsible for teaching ethics within rehabilitation programs across Canada.

**Analysis/Analyse:** A mixed-methods analysis was used.

**Results/Résultats:** Fourteen universities offer physical therapy programs in Canada. Content-analysis of websites revealed that 60 courses were relevant as they mentioned ethics. Of these, 23 syllabi were analyzed and among them 14 emphasised organizational issues. These reflect practical themes namely prioritization, legal obligations and healthcare delivery when third-party payers are involved. The credits attributed to these issues ranged from 0–7% by program. **Conclusions:** There is diversity between university physical therapy programs with regards to ethics teaching. Our ongoing survey will further illuminate which domains are addressed in training and what pedagogical methods are employed.

**Keywords/Mots clés:** Canadian university, ethics teaching, mixed-methods

# Abstract: A015 – Developing a New Assessment Tool for Canadian Physical Therapy Students in Clinical Education: Step 1: Item Reduction and Rating Scale Confirmation – A Delphi process

**Published:** 2013-05

**Authors:** B Mori, D Brooks, J Herold, D Beaton

**Affiliations:** * Department of Physical Therapy, Faculty of Medicine, University of Toronto, ON; † Centre for Faculty Development, University of Toronto at the Li Ka Shing International Healthcare Education Centre, St. Michael’s Hospital, Toronto,ON; ‡ Department of Medicine, Faculty of Medicine, University of Toronto, ON; § Keenan Research Centre, St. Michael’s Hospital; Occupational Sciences and Occupational Therapy, University of Toronto; ON

**Purpose & Objectives / Intention & objectifs:** The purpose was to determine which items from the Canadian Essential Competency Profile for Physiotherapists (ECP) should have a rating scale, a comment box and gain consensus on the rating scale to be used in the new national measure to assess physiotherapy (PT) in clinical education.

**Relevance/Pertinence:** A standardized tool to assess PT students’ clinical performance specific to Canadian needs does not exist. The Clinical Performance Instrument (CPI) is currently used to assess students in clinical education across Canada. Clinical Instructors and educators have expressed concerns that the CPI is time consuming, not always applicable in the practice setting and may have an American bias. PT educators have agreed that a new tool to assess Canadian PT students in clinical education component should be developed using the ECP as the framework for this tool.

**Materials & Methods/Matériel & méthods:** An online Delphi process was completed with an Expert Consultant Panel comprised of Academic Chairs/Directors and Clinical Coordinators of Canadian PT Programs (n=47).

**Analysis/Analyse:** Data were descriptively analysed. Consensus was set at 80% agreement.

**Results/Résultats:** Twenty-nine individuals responded to Round 1, 22 in Round 2 and 20 in Round 3. We gained consensus (85%) that the draft tool would have rating scales for 16 items and 9 comment boxes. Agreement on the rating scale was also achieved. **Conclusions:** Through the Delphi process, we were able to come to agreement on a draft tool to assess Canadian PT students in clinical education. Next steps will be to investigate face and content validity of this tool.

**Keywords/Mots clés:** physiotherapy, assessment, clinical education

# Abstract: A025 – Clinical Decision Making of Physical Therapy Graduates: Has it changed?

**Published:** 2013-05

**Authors:** PJ Manns, J Darrah

**Affiliations:** Department of Physical Therapy, University of Alberta, Edmonton, AB

**Purpose & Objectives / Intention & objectifs:** All physical therapy schools in Canada provide clinical education at a Master level. The purpose of this study was to compare decision making strategies of physical therapy graduates with graduate and undergraduate training.

**Relevance/Pertinence:** Changes in physical therapy education and their influence on practice behaviours is highly relevant.

**Materials & Methods/Matériel & méthods:** Invitations were mailed to physical therapy graduates of the University of Alberta. Recruitment focused on four cohorts (1996-2000; 2003-2005; 2005-2007; 2010), representing different combinations of years of experience and degree designation. An individual interview was conducted with each participant focusing on two clinical scenarios. Participants described their intervention decisions related to the scenarios, and identified the resources used to make decisions. Use of research evidence in decision making resulted in higher scenario scores. Three scores were derived from the interviews (total scores; clinical decision making scores on scenarios; general evidence based practice [EBP] scores).

**Analysis/Analyse:** A one-way analysis of variance was used to determine if there were differences between cohorts on the interview scores.

**Results/Résultats:** Eighty physical therapy graduates (57 females, 23 males) participated. Years since graduation ranged from 1–15 years. There were no differences among the cohorts on scenario scores, or the total score. Cohorts educated in a Master degree curriculum had higher scores on general EBP knowledge. **Conclusions:** Physical therapists educated in a revised (Master degree) curriculum had more theoretical knowledge of evidence based practice, but the resources that they used for clinical decision making were similar to physical therapists with undergraduate training.

**Keywords/Mots clés:** curriculum, clinical decision making, evidence based practice

# Abstract: A032 – Are physiotherapy continuing education courses a waste of weekends or do they actually positively change clinical practice?

**Published:** 2013-05

**Purpose & Objectives / Intention & objectifs:** To evaluate the efficacy of a short physiotherapy continuing education (CE) program on return to work outcomes of patients with work related low back injury.

**Relevance/Pertinence:** To date little research has been done on the efficacy of CE courses on direct patient outcomes of PTs who complete them.

**Materials & Methods/Matériel & méthods:** Fourteen PTs across the province of New Brunswick were selected to attend a CE course with focus on the treatment-based classification system of low back pain and 14 PTs who were not selected were used as the control.

We compared patient outcomes one-year pre-CE course to one-year post-CE course.

**Analysis/Analyse:** The independent t-test procedure and 2-tailed t-value were used to compare pre- and post-intervention cases.

**Results/Résultats:** In the year prior to the CE course 65% of the patients returned to work full-time full- duties, in the year following the CE course 85% of the patients returned to work full-time full-duties (p=0.007). The average number of sick days was reduced from 126 to 69 days in the year following the CE (p=0.001). There were no significant differences in patient outcomes in PTs who did not attend the CE course. **Conclusions:** To our knowledge this is the first time that PT attendance at a CE course has demonstrated actual changes in practice behaviour resulting in improved direct patient outcomes. Attendance at a CE course focusing on treatment-based classification was associated with significant improvements in clinical outcomes for patients with work related low back injury.

**Keywords/Mots clés:** continuing education, efficacy, low back injry

# Abstract: A044 – BONE FIT™: Knowledge and Attitudes of Physiotherapists and Kinesiologists

**Published:** 2013-05

**Authors:** D Hatzoglou, E Karkouti, L Cheng, J Laprade, L Giangregorio, R Jain, C Evans

**Affiliations:** * Department of Physical Therapy at the University of Toronto, Toronto, ON; † University of Waterloo, Kitchener, ON; ‡ Osteoporosis Canada, Toronto, ON

**Purpose & Objectives / Intention & objectifs:** The Bone Fit™ course includes online learning and a weekend workshop and was developed by Osteoporosis Canada to provide physiotherapists and kinesiologists with training on assessment and exercise prescription for individuals with osteoporosis. The purpose of this study was to determine whether the Bone Fit™ course resulted in a change in the knowledge and attitudes of participants regarding osteoporosis management. ’

**Relevance/Pertinence:** Physiotherapists and kinesiologists as part of a multidisciplinary team are in an ideal position to help clients manage osteoporosis.

**Materials & Methods/Matériel & méthods:** All learners enrolled in Bone Fit™ courses across Canada in 2012 were invited to participate in the current study. Bonefit is taught by a physiotherapist and combines online learning with a weekend workshop. A 27-item questionnaire was administered to those who consented using a web-based survey prior to and following course participation, and it included questions about osteoporosis knowledge (16 items), practice knowledge (4 items), attitudes (7 items) and demographic characteristics.

**Analysis/Analyse:** Pre- and post-course scores were compared using McNemar and Student t tests.

**Results/Résultats:** Thirty three of the 55 individuals who completed Bone Fit™ in 2012 participated in the study. There was a significant change in proportion of correct knowledge responses in ten items related to screening, medication, biomechanics and exercise (p<0.001) and two practice items (p<0.001). Participants were more confident about their role after the course in six items such as prescribing exercises and follow-up with physicians (p<0.001). **Conclusions:** Bone Fit™ improves physiotherapists’ and kinesiologists’ knowledge and attitudes related to osteoporosis. Future research will examine impact on practice.

**Keywords/Mots clés:** osteoporosis, physiotherapy, education, exercise

# Abstract: A054 – What Canadian Physical Therapy Clinical Instructors Want: Perspectives for a New Assessment Tool for Students in the Clinical Environment

**Published:** 2013-05

**Authors:** C Anderson, M Cosgrove, D Lees, G Chan, BE Gibson, M Hall, B Mori

**Affiliations:** * Department of Physical Therapy, University of Toronto, ON; † Department of Physical Therapy, University of Alberta, AB

**Purpose & Objectives / Intention & objectifs:** The purpose of this study was to gather Canadian Clinical Instructors’ (CIs’) perspectives on rating scales, training methods and format for the future development of a new tool to assess Physical Therapy students in clinical education.

**Relevance/Pertinence:** Many Canadian physical therapy programs use the Clinical Performance Instrument (CPI) to evaluate students in their clinical placements. Recent evidence suggests CIs’ discontent with the CPI and indicates a need to develop a new assessment tool. Consulting with CIs to inform the development of the tool will hopefully lead to the development of a feasible tool.

**Materials & Methods/Matériel & méthods:** Five focus groups were held across Canada. CIs who had supervised at least one Canadian student in clinic and who were English speaking were eligible for the study.

**Analysis/Analyse:** Focus groups were recorded and transcribed then thematically analysed.

**Results/Résultats:** Participants identified concerns with the CPI and indicated a preference for: 1) more objective rating scales with clearly defined anchors, 2) the availability of both in-person and online training methods, and 3) a tool which could be completed and reviewed on paper or online. Participants agreed that the Essential Competency Profile was an appropriate framework for the tool. **Conclusions:** CIs affirmed the need for the development of a new assessment tool. Results of the study will be used in the development of a new assessment tool to better evaluate Canadian physical therapy students in the clinical setting.

**Keywords/Mots clés: Key Words:** clinical education, physical therapy, students, assessment tool

# Abstract: A064 – Social Media – A New Tool for Increasing Public Awareness of Chronic Conditions

**Published:** 2013-05

**Authors:** S Prasanna, M Simmonds

**Affiliations:** * School of Physical and Occupational Therapy, McGill University, Montreal, QC; † Centre for Interdisciplinary Research in Rehabilitation of Greater Montreal (CRIR), Montreal, QC

**Purpose & Objectives / Intention & objectifs:** Providing health information to clients forms an integral part of health promotion and disease prevention strategies. Social media such as Twitter and Facebook are being increasingly used to provide and share information. Therefore, it is timely to examine the potential of social media in health promotion.

**Relevance/Pertinence:** The role of physiotherapists is expanding from being primarily involved in illness management to providing health promotion and preventive services. Social media can be a useful tool to educate clients.

**Materials & Methods/Matériel & méthods:** A narrative review was conducted. PubMed, CINAHL and Embase databases were searched using pre-determined search terms.

**Analysis/Analyse:** Relevant literature in social media and health promotion was summarized and discussed in the context of the use of social media to improve layperson awareness of chronic conditions.

**Results/Résultats:** Social media is a group of online applications allowing users to create and exchange content. Types include – collaborative projects, social networking sites, content communities, blogs and microblogs, virtual social worlds and virtual game worlds. Social media is mainly used by key target populations for prevention campaigns namely young and middle-aged adults. Previous research has shown that chronic diseases are social in nature and adoption of health behaviours is socially motivated. Hence these media can be useful. It can contribute to greater engagement of the public and increase information sharing contributing to spreading important health messages. It can help promote empowerment and self- efficacy of the client. **Conclusions:** Understanding the utility of social media will help rehabilitation professionals to strategically use novel risk reduction/health promotion approaches in their practice.

**Keywords/Mots clés:** social media, chronic diseases, health promotion

# Abstract: A076 – Correlation between Global Rating Scale and Specific Checklist Scores for Professional Behaviour of Physical Therapy Students in Practical Examinations

**Published:** 2013-05

**Authors:** K Turner, M Bell, L Bays, C Lau, C Lai, T Kendzerska, R Davies, C Evans

**Purpose & Objectives / Intention & objectifs:** The purpose of this study was to determine whether or not checklist and global rating scale scores are correlated.

**Relevance/Pertinence:** The checklist only considers specific items students must perform, and is not truly indicative of professional behaviour in the clinical setting. Alternatively, the global rating scale is more suitable as it allows examiners to capitalize on their experience to identify more subtle behavours.

**Materials & Methods/Matériel & méthods:** Professional behaviour was evaluated using both the checklist and global rating scale for 183 students in three practical skills examinations.

**Analysis/Analyse:** Mean, standard deviation, and correlation for checklist and global rating scale scores were calculated for each station, within each practical skills examination. Pass rate for checklist and global rating scale was determined for each practical skills examination, as well as for each individual checklist item, within each practical skills examination.

**Results/Résultats:** Overall, pass rate was high for both checklist and global rating scale evaluations of professional behaviour in all practical skills examinations. Generally, mean scores for the checklist and global rating scale were high, with low standard deviations, resulting in low data variability. Spearman correlation between total checklist and global rating scale scores were statistically significant for two of five stations in practical skills examination number one, five of six stations in number two, and three of four stations in number three. **Conclusions:** The global rating scale is comparable to the checklist for evaluation of professional behaviour in physical therapy students and appears to become stronger in the assessment of more advanced students.

**Keywords/Mots clés:** global rating scale; checklist; clinical skills examination; physical therapy; students

# Abstract: A077 – Program evaluation of a model to prepare internationally educated Physiotherapists for the Physiotherapy Competency Examinations

**Published:** 2013-05

**Authors:** A Greig, D Dawes, S Murphy, G Parker, B Loveridge

**Affiliations:** * Department of Physical Therapy, The University of British Columbia, 212 - 2177 Wesbrook Mall, Vancouver, BC; † UBC Faculty of Medicine, 2211 Wesbrook Mall, Koerner Pavilion, Vancouver, BC

**Purpose & Objectives / Intention & objectifs:** To create a program comprised of the educational tools and infrastructure to support internationally educated physiotherapists (IEPs) in their preparation for entry to practice in Canada by improving their pass rate on the national competency examination.

**Relevance/Pertinence:** The demand for Physiotherapists in Canada continues to increase. The program was developed to provide support for IEPs in their preparation for taking the National Physiotherapy competency examinations.

**Materials & Methods/Matériel & méthods:** The program was developed using a logic model and evaluated using program evaluation methodology. Program tools and resources were developed and refined based on feedback from clinical experts, IEPs and clinical physical therapy mentors.

**Analysis/Analyse:** Program Evaluation was initiated with the formation of a program logic model followed by analysis of the outcomes defined in the model. The differences between National exam results for IEPs who had participated in the IEP Program compared with other internationally educated Physiotherapists who had not participated in the program were tested using Fisher exact test.

**Results/Résultats:** The pass rate for IEPs taking the national written exam between 2008 and 2012 is 0.67, and for those taking the national practical exam is 0.92. These rates are significantly higher (p<0.05 and p<0.001, respectively using Fisher exact test) than the pass rate for all the internationally-educated physiotherapists who the exams (0.53 and 0.68, respectively). **Conclusions:** The program has proven to be successful and sustainable. This program model could be replicated to support the successful integration of IEPs in other provinces across Canada.

**Keywords/Mots clés:** Internationally educated physiotherapist; exam preparation; educational program development

# Abstract: A078 – Utilisation d’exemples résolus («worked-examples») en ligne pour développer le raisonnement clinique d’intervention chez des étudiants en physiothérapie

**Published:** 2013-05

**Authors:** JO Dyer, A Hudon, K Montpetit-Tourangeau, S Mamede, Gog T van

**Affiliations:** * Programme de physiothérapie, École de Réadaptation, Université de Montréal, Montréal, QC; † Département de psychologie, Erasmus University Rotterdam, Rotterdam 3062 PA, Pays-Bas

**Purpose & Objectives / Intention & objectifs:** Les exemples résolus sont des outils pédagogiques où l’apprenant étudie la solution d’un problème déjà résolu (Atkinson, 2000). Ils ont été utilisés avec succès pour développer le raisonnement clinique diagnostique chez des étudiants en santé (Boekhout, 2010). Aucune étude n’a évalué l’impact de ces exemples sur le raisonnement d’intervention. Cette étude vise à évaluer: 1- si l’étude d’exemples résolus améliore le raisonnement d’intervention chez des étudiants débutants en physiothérapie ; 2- si l’ajout d’étude de cartes conceptuelles modèles ou de construction de cartes améliore l’apprentissage.

**Relevance/Pertinence:** Le développement du raisonnement d’intervention est un objectif important des programmes de physiothérapie. Il importe donc d’évaluer les meilleures stratégies pour faciliter son apprentissage.

**Materials & Methods/Matériel & méthods:** 67 étudiants de 2ième année en physiothérapie ont participé à une séance de 2 heures d’apprentissage du raisonnement d’intervention à l’aide d’exemples résolus par des experts, dans un environnement numérique d’apprentissage. De plus, selon une répartition aléatoire en 3 groupes, ils ont également fait soit: de l’étude personnelle (groupe A) ou l’étude de cartes conceptuelles modèles (B) ou la conception de cartes conceptuelles (C).

**Analysis/Analyse:** Des analyses de variance multi et univariées (Tukey post-hoc) ont été réalisées.

**Results/Résultats:** L’étude des exemples résolus d’intervention a amélioré la performance à un test de raisonnement clinique en ligne. De plus, l’étude des cartes conceptuelles modèles a entraîné une amélioration de la performance significativement supérieure à la construction des cartes. **>Conclusions:** Les exemples résolus pourraient être utiles à l’apprentissage du raisonnement d’intervention chez des étudiants débutants en physiothérapie.

**Keywords/Mots clés:** Raisonnement clinique, environnement numérique, apprentissage, exemples résolus, cartes conceptuelles

# Abstract: A079 – Documenting students’ exposure to a variety of clinical experiences using an electronic tool

**Published:** 2013-05

**Authors:** M Denis, I Savard, H Moffet, G Bourdeau, A Elkadhi

**Affiliations:** Département de réadaptation, Faculté de médecine, Université Laval, Québec, QC

**Purpose & Objectives / Intention & objectifs:** Universities’ physiotherapy program must demonstrate they expose their students to a variety of clinical placement experiences.

**Relevance/Pertinence:** To meet this requirement, we developed a new electronic tool to monitor clinical exposure. The objectives of this report are to: 1) describe its functions, 2) present preliminary results.

**Materials & Methods/Matériel & méthods:** The developed tool is adapted from a pre-existing tool. It is supported by a local server and confidentiality is ensured using passwords and individual identifiers. Critical information to collect was identified by the program and development was ensured by a system architect. It was implemented in September 2010.

Students are responsible for collecting information on a day to day basis during clinical placements. More precisely, information on the characteristics of the clients, exposure to PT expertise competency components and their level of responsibility in each clinical situation is collected. The tool is user-friendly providing choices in a dropdown menu. Supervisors validate online the information captured by students.

**Analysis/Analyse:** Summaries (tables and graphs) of exposure are generated. The Faculty responsible for the clinical placements has accessed to these summaries while individual information is provided to each student and corresponding supervisors.

**Results/Résultats:** Preliminary results show that, at the middle of the fourth year (66 % of the placements completed), students were exposed to situations involving the musculoskeletal system (65%; neurologic and cardiorespiratory, 23% and 10%) in adults over 50 years (56%; children 13%). More comprehensive summaries presented to PEAC were appreciated. **Conclusions:** We will continue to refine this tool knowing that its appropriateness is demonstrated.

**Keywords/Mots clés:** clinical placement exposure, electronic tool

# Abstract: A080 – Unpacking the Advocate Role: Exploring How Physical Therapy Departments in Canadian Universities Can Further Develop the Advocate Role

**Published:** 2013-05

**Authors:** M McGuire, J Yu, K Kelland, E Hoe, A Andreoli, S Nixon

**Affiliations:** * Department of Physical Therapy, University of Toronto, Toronto, ON; † International Centre for Disability and Rehabilitation; University of Toronto, Toronto, ON; ‡ University Health Network - Toronto Rehabilitation Institute, Toronto, ON

**Purpose & Objectives / Intention & objectifs:** To explore the perspectives of cutting-edge advocates on how physical therapy departments in Canadian universities can develop the advocate role found within the Essential Competencies Profile for Physiotherapists in Canada.

**Relevance/Pertinence:** The importance of the advocate role in physical therapy is highlighted by its inclusion as one of seven core competencies in the Essential Competency Profile for Physiotherapists in Canada. Results from this study will identify ways that physical therapy departments in Canadian universities can further develop this competency.

**Materials & Methods/Matériel & méthods:** This descriptive qualitative study involved semi-structured interviews conducted with 17 Canadian cutting-edge advocates with knowledge of the workings of physical therapy departments.

**Analysis/Analyse:** Transcribed interviews were coded and analyzed using thematic analysis.

**Results/Résultats:** Participants identified that physical therapy departments can develop the advocate role in four key areas: promoting a curriculum that emphasizes this role in students, engaging in advocacy- promoting research, role-modeling advocacy among faculty and engaging in policy-oriented advocacy. Participants recommended that curricula should include complementary educational approaches that are multimodal, integrative and progressive and should present the advocate role as an integration of the other core competencies as well as three additional attributes: perseverance, passion and humility. **Conclusions:** Physical therapy departments in Canadian universities can play a central role in developing the advocate role within the physical therapy profession. Future research should evaluate the advancement of the advocate role within physical therapy departments and explore the perspectives of other stakeholders.

**Keywords/Mots clés:** advocate role; advocacy; physiotherapy; competency-based education; curriculum

# Abstract: A082 – Clinical Placements: Do we really know what students are doing?

**Published:** 2013-05

**Authors:** S Murphy, A Greig

**Affiliations:** Department of Physical Therapy, University of British Columbia, Vancouver, BC

**Purpose & Objectives / Intention & objectifs:** Physical therapy students undertake clinical placements in a wide variety of clinical settings with diverse caseloads. Accurate tracking of clinical experiences is challenging and has not been widely carried out.

**Relevance/Pertinence:** A detailed record of individual learning experiences allows identification of individual student learning needs and effective planning for future clinical exposure during physical therapy education.

**Materials & Methods/Matériel & méthods:** Software developed for tracking clinical learning experiences of medical residents was redesigned for Physical Therapy use, with the goal of generating a detailed clinical log for individual students in the Master of Physical Therapy program, as well as providing detail on cohort experiences. Clinical experts as well as students were involved in the development process.

**Analysis/Analyse:** Simple statistical analysis of system-generated reports provided data related to student caseload, student participation in the clinical setting, and individual student performance compared to class norms.

**Results/Résultats:** The system has enabled the collection of new data with significant detail related to student experience in the clinical setting. Information related to the frequency of diagnosis encountered, assessment and treatment techniques performed, and student level of independence was easily extracted from the system. Individual student performance against class norms could also be easily identified.

**Conclusions:** Our web based tool provided access to data related to student performance and caseload in the clinical setting, enhancing the identification of learning needs and development of individualized learning plans for physical therapy students. Future uses for system data include curricular planning and student assessment.

**Keywords/Mots clés:** Clinical Education, Clinical Log

# Abstract: A083 – Impacts d’une formation sur le raisonnement clinique adressée aux superviseurs de stages en physiothérapie de l’Université de Montréal

**Published:** 2013-05

**Authors:** J Montreuil, C Besner

**Affiliations:** * Programme de Physiothérapie, Université de Montréal QC; † Programme de Physiothérapie, Université de Montréal, QC

**Purpose & Objectives / Intention & objectifs:** Mesurer les impacts d’une formation interactive offerte aux superviseurs cliniques au programme de physiothérapie sur l’encadrement du raisonnement clinique (RC) des stagiaires.

**Relevance/Pertinence:** Le RC est la pierre angulaire du cadre pédagogique de notre programme de physiothérapie. La supervision des étudiants en milieu clinique représente une occasion privilégiée d’accompagnement du développement du RC. En 2010, une formation sur le RC a été développée pour les superviseurs de stage en physiothérapie afin d’arrimer ceux-ci à notre cadre pédagogique.

**Materials & Methods/Matériel & méthods:** Questionnaire en ligne anonyme (10 questions) avec échelle de Likert à 4 niveaux sur les impacts de la formation du RC conduit auprès de 123 superviseurs.

**Analysis/Analyse:** Une analyse quantitative des résultats a été conduite en mars 2012.

**Results/Résultats:** Des 69 répondants, 42 avaient supervisé des stagiaires et mis en pratique les concepts acquis dans cette formation. Les résultats spécifiques à l’impact de la formation démontrent que, parmi les 42 superviseurs, l00% se disent capables de poser un diagnostic pédagogique, 95% de déterminer des objectifs de développement et 93% de mettre en place des stratégies pour améliorer le RC des stagiaires. Tous les superviseurs confirment que la formation leur a permis de mieux encadrer le développement du RC des stagiaires. **Conclusions:** Les résultats nous démontrent que la formation a des impacts positifs sur le sentiment de compétence des superviseurs à mieux encadrer le développement du RC de stagiaires. Une seconde étude nous permettrait de mesurer les retombées de cette formation au niveau de l’encadrement du RC en stage.

**Keywords/Mots clés:** raisonnement clinique, enseignement clinique, stagiaires

# Abstract: A084 – Reliability of practical skills assessment in a distributed physical therapy education context

**Published:** 2013-05

**Authors:** A Richter, GP Bostick, E Parent, M Barnes, C Brososky

**Affiliations:** * Faculty of Rehabilitation Medicine, University of Alberta, Edmonton, AB; † Assistant Professor, Department of Physical Therapy, University of Alberta, Edmonton, AB; ‡ Associate Professor, University of Alberta and Clinical Scientist Glenrose Rehabilitation Hospital, Edmonton, AB; § Physical Therapist, Glenrose Rehabilitation Hospital, Edmonton, AB; ¶ Physical Therapist and Director, Westend Physical Threapy Clinic; Edmonton, AB

**Purpose & Objectives / Intention & objectifs:** Evaluate the reliability of student practical skills assessment scores obtained from video recordings.

**Relevance/Pertinence:** Reliable video assessment of physical therapy (PT) students’ clinical skills increases accessibility to distance learning and offers flexibility in assessment.

**Materials & Methods/Matériel & méthods:** Practical skills assessment of 68 MScPT students was scored by three raters. Each student was assessed face-to-face on a spine mobilization and an exercise prescription task using a checklist. The exam was recorded by the Lifesize videoconferencing system. A total score was calculated for each student. Raters each scored 22 or 24 students face-to-face and after three months re- scored performances using video-recordings of all 68 students. Qualitative data about factors perceived to influence video scoring were also recorded.

**Analysis/Analyse:** Intraclass correlation coefficients ICC(2,1) with 95% confidence interval were calculated for each rater’s pair of face-to-face and video-recording scores (intra-rater/inter-method reliability) and agreement across raters’ video-recorded scores (inter-rater reliability).

**Results/Résultats:** For total scores, intra-rater ICC coefficients for two examiners were acceptable [0.75(0.50,0.88) and 0.70(0.40,0.86)] and poor for the other examiner [0.47(0.06,0.74)]. The poor intra- reliability may be due to intermittent background noise and instances where students’ did not face the camera. The inter-rater ICC coefficient for the video assessment total scores was poor [0.42(0.24, 0.60)]. This may be due to the less experienced raters providing higher scores and also demonstrating less variance in their scores compared to the experienced examiner. **Conclusions:** Evaluating practical skills via distance education demonstrated acceptable levels of intra-rater reliability when optimizing recording quality. Further investigation is planned to ascertain the source of the poor inter-rater reliability.

**Keywords/Mots clés:** Reliability, Distance or Distributed Education, Physical Therapy

# Abstract: A085 – Development of a feedback tool for intraprofessional practice

**Published:** 2013-05

**Authors:** W Jelley, N Larocque, M Borghese

**Affiliations:** * University of Ottawa, Physiotherapy Program, Ottawa, ON; † La Cité Collégiale, OTA & PTA Program, Ottawa, ON; ‡ University of Ottawa, Faculty of Health Sciences, Ottawa, ON

**Purpose & Objectives / Intention & objectifs:** The purpose of this research project was to develop, pilot and refine a feedback tool that could be used by physiotherapy and physiotherapist assistant students during clinical placements.

**Relevance/Pertinence:** Collaboration between physiotherapists and physiotherapist assistants is necessary for effective and efficient patient care. We designed a tool that encourages reflection and communication between physiotherapy and physiotherapist assistant students during clinical placements. We hope this will help to encourage collaboration.

**Materials & Methods/Matériel & méthods:** A draft of the tool in English and French was developed by educators in a physiotherapy and physiotherapist assistant program. Key elements of the tool came from the results of the essential competencies for intraprofessional practice survey. For content validity, a group of French and English subject matter experts from education, research and the clinical community reviewed the tool. These individuals were chosen for their expertise in intraprofessional practice or instrument development. The revised tool was then reviewed by a larger audience, with French and English versions compared for equivalency.

**Analysis/Analyse:** In April 2012, 3 teams of physiotherapy and physiotherapist assistant students piloted the 16-item tool during paired clinical placements. The students and their clinical instructors were encouraged to use the tool to reflect, self-evaluate and give feedback at mid-placement.

**Results/Résultats:** Twelve participants used the tool. Qualitiative feedback from the physiotherapy students suggested that the tool stimulated discussion about collaboration and strategies for effective patient care.

**Conclusions:** The encouraging results from the pilot suggest the feedback tool for intraprofessional collaboration shows potential for use with physiotherapy and physiotherapy assistant students during clinical placements.

**Keywords/Mots clés:** Physiotherapist, physiotherapist assistant, Intraprofessional, collaboration, feedback

# Abstract: P003 – Bridging for Success: The Development, Implementation and Role of Bridging Programs for Internationally Educated Physical Therapists

**Published:** 2013-05

**Authors:** S Switzer-McIntyre, B Norton, C Puri

**Affiliations:** * Department of Physical Therapy, University of Toronto, Toronto, ON; † Department of Physical Therapy, University of Alberta, Edmonton, AB; ‡ Department of Physical Therapy, University of Alberta, Edmonton, AB

**Overview & Objectives/Résumé & objectifs:** At the end of the session the participant will be able to:

1. Appreciate first hand, through dialogue with a recent Bridging Graduate, the journey internationally educated physical therapists travel to become licenced to practice in Canada
2. Describe the knowledge and skill gaps of the internationally educated physical therapist
3. Describe the purpose and curriculum of Internationally Educated Physical Therapy Bridging Programs in Alberta and Ontario
4. Describe the distributed distance teaching and learning strategies utilized
5. To understand how currently licensed physical therapists can support integration initiatives for the internationally educated physical therapists

**Relevance/Pertinence:** Bridging Programs are designed to assist physical therapists educated outside of Canada (IEPT’s) to bridge their educational and clinical practice gaps and facilitate successful integration into the Canadian health care system. Increasing demand for physical therapy services related to the aging population and chronic long term conditions, the projected workforce shortages, and the expanding cultural diversity within Canada makes IEPTs and bridging programs important to physical therapy as a profession and to the health of Canadians.

**Target Population/Population cible:** This session will be of interest to licensed physiotherapists who work directly or indirectly with IEPTs, managers of PT departments/clinics who hire physiotherapists and IEPTs currently working as Physiotherapy Assistants.

**Supporting Evidence/Résumé des faits:** CIHI Data, as reported in the Workforce Trends in Physiotherapists in Canada, 2009, substantiates the evidence that Ontario has a skills shortage. More than 25% of physiotherapists practicing in Ontario are over 50 years of age and many of these physiotherapists are expected to retire within the next ten years. While the number of new Canadian physiotherapy graduates mitigates some of the potential attrition, there are not enough new graduates to replace those expected to retire. IEPTs are, and will remain, an important source of physical therapy supply in Ontario.

Based on the analysis of the Ryerson Pilot Project, the initial cohort at the University of Toronto and the pass rates in the National Physiotherapy Competency Examination it is evident that gaps in independent practice and evidence based practice are major learning challenges for IEPTs requiring significant attainment of new knowledge, skills and clinical judgment. The gaps indicate IEPTs were not primary care professionals in their source countries and relied on a physician for assessment, diagnosis and developing a treatment plan.

**Session Format/formule de la session:** This presentation will feature an internationally educated physical therapist bridging graduate as well as faculty members from the universities who will highlight the key curriculum areas of the Internationally Educated Physical Therapy Bridging Programs in Alberta and Ontario. These curricular areas will be mapped against the learning goals of an IEPT as they progress towards their goal of licensure to practice in Canada. The key factors that affect successful integration of IEPT’s will be highlighted, as will the collaborative projects across the country and the many opportunities available for currently registered physical therapists to become involved in the IEPT’s journey.

The session will involve active participation of the audience in the form of role playing and the use of i- clickers to enhance the participants understanding of some of the cultural and learning issues faced by the IEPT’s.

**Conclusions & implications:** At the end of the session, participants will have a better understanding of the learning journey that IEPTs travel as they immigrate to Canada. They will also understand the role of the bridging programs in that journey as well as the potential roles they may assume to participate in the journey.

**Keywords/Mots clés:** Internationally Educated Physical Therapist Bridging Program

# Abstract: P009 – Social Learning and Professional Development

**Published:** 2013-05

**Authors:** M Prior

**Affiliations:** University of Ottawa, Faculty of Education, Lamoureux Hall (LMX), 145, Jean-Jacques-Lussier Private, Ottawa, ON

**Overview & Objectives/Résumé & objectifs:** The following session will be based on a study that looked at the professional development of physiotherapist working in long-term care. As part of the literature review and conceptual framework, concepts on professional socialisation, professional culture, professional regulation, communities of practice and social learning were explored.

A survey was created based on relevant literature for this study. It was reviewed and piloted before an email invitation was sent to all members of the Ontario Physiotherapy Association. 44 participants responded to the survey which represents 10% of physiotherapists working in long-term care at that time.

The results indicate that physiotherapists are isolated from their physiotherapist peers and lack access to communities of practice, professional socialisation, professional culture and social regulation. Although physiotherapists’ interactions with interprofessional teams added breadth to their knowledge, these interactions did not enhance their profession-specific skills. Many physiotherapists are seeking professional community and social supports in healthcare settings outside of the long-term care context.

The implications of this study are that physiotherapists, their professional associations, and their college must understand the importance of professional socialization in learning, and ensure that physiotherapists working in long-term care have access to and seek such social support. Physiotherapy service providers in long-term care should provide mentoring, support and opportunities for social learning for their clinicians.

This session will explore professional development in physiotherapy and social learning in work place. Learning objectives:

1. Understand the importance and impact of social learning in the workplace
2. Understand and discuss use of social learning activities in the workplace to enhance professional development
3. Understand the barriers to implementation of social learning activities

**Relevance/Pertinence:** Physiotherapists prefer social learning activities. Professional socialisation is also important for clinical reasoning and critical thinking in physiotherapy. It is important that we recognize the importance of social learning and that we capitalize on our current knowledge resources in the work place.

**Target Population/Population cible:** Physiotherapy managers, clinical educators and clinicians who want a better understanding of effective professional development activities.

**Supporting Evidence/Résumé des faits:** Numerous systematic reviews (Onion & Bartzokas (1998); Pennington et al. (2005); Bero (1998); Grimshaw, Shirran & Thomas (2001)) have confirmed that continuing education activities which promote research uptake have achieved non-significant results. In support of this argument, an ethnographic study of nurses and medical doctors was carried out by Gabbay & le May (2004), it concluded that healthcare professionals rarely accessed and used current research literature to gain knowledge. As part of Hall’s 2005 survey, when asked what method of continuing education physiotherapists preferred, they listed, among other choices, searching the internet for evidenced based practices. Hall (2005) emphasized the difference between searching the internet for information and using the internet for the purpose of on-line academic research. It was found that most therapists in the United States had limited access to online journals and academic data bases. In Rappolt and Tassone’s 2002 survey, they also found that literature searches were secondary sources of information and in their study, more than half of the participants reporting rarely or never using this type of information. (Rappolt and Tassone, 2002). Physiotherapists are not effective consumers of research, they also lack access to online journals and academic data bases.

Bleakley (2006) states that the research on learning theories in medical education continues to focus on the individual learner, rather than the socio-cultural context of learning. Vygotsky hypothesised that learning and development at the individual level are the product of social and cultural environments (Davidov, 1995). The social and physical contexts that give shape to individual thinking include conceptual artefacts like language and physical ones like tools and computers, as well as collaboration with peers and teachers. A learner’s interaction with his/her environment and with communities of other learners thus play a central role in Vygotsky’s ideas. This social constructivist approach is echoed by Kuhn, Greeno, Lave, Simon and Brown (Lui & Matthews, 2005).

Professional practice culture is “the medium through which people’s understanding of work practices, attitudes and behaviour are learned and shaped” (Richardson, 1999). Physiotherapy practice culture is developed through social interactions which take place in physiotherapy departments, such as hospitals, private clinics and rehabilitation departments (Richardson, 1999). Through professional practice culture, physiotherapists’ receive professional socialisation and professional regulation, which influences their practice and work behaviours. Professional learning is thus also professional socialisation in which “individuals learn the values, attitudes and beliefs of their chosen profession and develop a commitment to a professional career” (Richardson, 1999). Through situational social exchanges physiotherapist assimilate professional values and social consensus of professional behaviours (Lave, 1988: Richardson, 1999). A physiotherapy practice culture can be seen as an unrecognized community of practice (Wenger et al., 2002) in which members discuss practice issues over lunch and between consultations with clients. These informal conversations become the main source of knowledge for the group. Their concern is their patients and their passion is physiotherapy.

Modelling behaviour during social interactions is an important part of professional socialization. “Modelling [is] used to learn to perform practical skills, communication skills and importantly, thinking (or reasoning) skills, in a way similar to that of mentors and colleagues” (Ajjawi & Higgs, 2008). Socialization with mentors and peers is also crucial to challenging learners’ preconceived values and behaviours. This often occurs during professional schooling; however it also occurs in the workplace. “Students already have their own sets of values and beliefs, which may change during the socialisation process to reflect the values the profession holds in high esteem. The change in values will lead to a change in behaviour and to the formation of a professional identity” (Ajjawi & Higgs, 2008).

Another important aspect of professional socialisation is professional or social regulation. As an individual learner, one is not always capable of assessing his or her own limitations or gaps in knowledge. This was a conclusion in Eva & Regehr’s (2005): “Self assessment does not appear to be a stable, global skill that is easily acquired or developed but rather it is situationally bound and context specific; therefore, feedback from ‘reliable’ others is necessary to inform our ability to judge our actions and decisions”. Physiotherapists in long-term care lack such reliable others and the feedback loop they offer. This may negatively influence their ability to critically reason and reflect on their practice.

**Session Format/formule de la session:** 50% lecture base on social learning in the work place, power point presentation and handout. 50% discussion regarding strategies of implementation of social learning in the work place.

**Conclusions & implications:** Social learning is an important component of professional development and must be fostered in our work places. Physiotherapists and their professional associations must ensure that physiotherapists have access to social learning forums. These social learning forums could take the form of nights of education, rounds, in-services, videoconferencing, teleconferencing, monthly in-person meetings, or social media forums such as Facebook. The professional associations should also educate physiotherapists on the effects of isolation on their learning and clinical reasoning, as well as on strategies to counter its effects such as seeking professional social contact and peer support for their professional development. Physiotherapy service providers also need to ensure that professional socialisation is available to their therapists.

**Keywords/Mots clés:** Social learning, professional development, physiotherapy

# Abstract: P022 – Practice what we preach: Implementing interprofessional student placements using a collaborative placement model

**Published:** 2013-05

**Authors:** N Littke, M Hall

**Affiliations:** * Glenrose Rehabilitation Hospital, Edmonton, AB; † Department of Physical Therapy, University of Alberta, Edmonton, AB

**Overview & Objectives/Résumé & objectifs:** The purpose of this session is to present the experiences and lessons from a group of rehabilitation clinicians, faculty members and students involved in establishing and enhancing an interprofessional student service within an outpatient program at a rehabilitation hospital. In an effort to improve patient access, enhance interprofessional collaboration and increase student placement opportunities, the partners in this program (rehabilitation clinicians, managers and university educators) collaborated to establish an innovative interprofessional student placement with the purpose of providing student centered, interprofessional clinical education opportunities within an existing rehabilitation program. One clinician in each of Occupational Therapy, Physical Therapy and Speech-Language Pathology was hired to provide clinical supervision and mentorship to students. Each clinical supervisor supervises 3–5 students at a time and provides access to these placements continually throughout the year to both local and out of province students to ensure continuity of service. The three clinicians collaborate to enhance the quality and number of student placement opportunities in an interesting practice setting, and together with their students, improve patient care through the development of innovative service delivery models in both individual and group formats. This session will describe the processes involved in establishing a student focused clinical service, explore the challenges and successes associated with establishing such a service, and discuss the changes undergone in the program over 15 months based on student, clinician and patient feedback to enhance the interprofessional and collaborative practice opportunities for the student participants through the provision of formalized interprofessional education.

The learning objectives of this session are threefold: 1) To describe the growth of this program from an idea to actuality over a two year period. 2) To present the challenges faced in offering this alternative placement model and highlight the solutions to these challenges as they developed over the course of the first year. 3) To provide implementation recommendations for the purpose of developing similar models of clinical education and patient care at other health institutions.

**Relevance/Pertinence:** Clinical education is an integral component of physiotherapy student training; however, the pressures of the healthcare environment coupled with increasing student enrolment to meet physiotherapy workforce requirements places increasing demands on frontline clinicians to meet current and future clinical placement requirements. With the move toward truly client-centered care, and the need to manage limited healthcare resources, the need to practice both collaboratively and interprofessionally within the healthcare environment is becoming a priority with providers of healthcare at all levels. The institutions educating new professionals must strive to instill this practice philosophy within its students to ensure they can both practice this model of healthcare delivery and facilitate the growth of existing clinicians from the more traditional, multi-disciplinary type of practice within the medical model of healthcare delivery to true interprofessional practice. Although the academic institutions can provide excellent theoretical knowledge and learning opportunities to students from all of the medical disciplines, adult learning theories identify the need for students to practice this within their clinical placements as crucial to their learning. This model of clinical education delivery may begin to mitigate some of the clinical placement shortages, may improve patient outcomes while enhancing interprofessional student experiences and serve as a basis for the development of similar programs across the country.

**Target Population/Population cible:** This session will be of interest to a broad range of professionals including clinicians, managers, professional and academic leaders, educators and researchers interested in the implementation of and delivery of interprofessional educational opportunities.

**Supporting Evidence/Résumé des faits:** Interprofessional education and collaborative practice is viewed as a strategy to address the current crisis in health workforce and service delivery. Collaborative practice occurs when professionals from different disciplines work together, as opposed to alongside one another, with families and caregivers to deliver the highest standard of patient centered care and ultimately improve health outcomes (World Health Organization, 2010). Successful implementation of collaborative practice requires structural support (such as an environment which supports collaboration, administrative support, and space for collaboration), knowledge of the collaborative process and personal commitment from the participants (Bronstein, 2003).

Interprofessional education is a key concept in ensuring new professionals are prepared for a collaborative practice work environment. Although clinical competency in each discipline must be ensured through the education provided, competency in four areas of interprofessional practice has been identified as crucial to the provision of collaborative patient care. The National Interprofessional Competency Framework (2010) identifies these competencies as they relate to communication, conflict resolution, role clarification among providers and collaboration. However, the provision of interprofessional clinical opportunities for students is challenging in an environment where there is a shortage of clinical placements and front line clinicians working in more traditional practice models are overwhelmed with workloads and patient acuity.

Student-led and student focused treatment clinics are one alternative to mitigate the shortfall in student placement opportunities, while enhancing interprofessional student collaboration, particularly in underserved populations (Ellett, Campbell & Gonsalves, 2010; Meah, Smith & Thomas, 2009). Students are placed at the forefront of patient care and clinical decision making while providing a necessary service, adding richness to their overall clinical education (Ellett et al. 2010). In this context, students can also increase the profile of collaborative practice in the work setting of the treatment clinic.

**Session Format/formule de la session:** This session would be a combination of lecture and small group discussion to brainstorm issues apparent with the current model of clinical education as well as barriers and strategies to address implementing an interprofessional, multiple students to clinical educator model.

**Conclusions & implications:** A shortage of clinical placement opportunities is apparent in many practice settings and areas of practice in regions across the country. As well, the need to provide collaborative, interprofessional care is a priority in the delivery of health care around the world.

An interprofessional student-focused care delivery model within an existing outpatient service is an innovative and exciting strategy that will begin to address clinical placement shortages, increase patient access to care, increase facility productivity through force multiplication while facilitating the provision of client focused interprofessional care.

The goal of this session is to address the idea of offering an increased number of, collaborative and interprofessional clinical placements to students across the country and to learn successful and innovative strategies from the experiences of clinicians, clinical supervisors, managers, educators and students for implementation elsewhere

**Keywords/Mots clés:** Interprofessional Education, Collaborative placements, Clinical Education

# Abstract: D009 – How Can I Be a Transformational Leader? Exploring and Applying Transformational Leadership: Leadership Division Workshop

**Published:** 2013-05

**Authors:** Lowery C Damp, L Sinclair

**Affiliations:** * Holland Bloorview Kids Rehabilitation Hospital, Toronto, ON; † Centre for Interprofessional Education, University of Toronto, Toronto, ON

**Objectives/Objectifs:** Upon completion of this session, participants will be able to:

1. Identify the current fields of emerging cognitive leadership theories
2. Describe and define transformational leadership within the physiotherapy profession
3. Explore the application of transformational leadership in cases and in their own practice

**Background/Contexte:** Opportunities for physiotherapists to take on leadership roles are diminishing in publicly funded institutions. Private practice clinicians are increasingly required to act as leaders in their business of health care. As the pool of leaders in physiotherapy decreases, the Leadership Division is creating a Leadership Education series to help grow the leadership competencies of the profession. The vision of the Leadership Division is to build curriculum of core competencies in professional self-awareness, modeling leadership behaviours and acknowledging qualities in others within the profession, and the health care system through networking, communication, education and research. Using a well-developed Logic Model for Leadership Education Series, the division will help to provide resources to physiotherapists to improve their leadership skills and help physiotherapists move into leadership positions.

From Sept 2010 through June 2011, the Leadership Division Executive designed a project charter and in July 2011 hired a consultant with expertise in professional curriculum and the development of leadership skills. This innovative curricular program based on current evidence, literature and best practices in leadership was shared at Congress 2012 to a large audience.

The Leadership Division would now like to share the current state of leadership theories and highlight emerging cognitive constructs for leadership. Transformational leadership will be discussed in detail and participants will be encouraged to reflect and apply these skills to practice cases and personal situations.

**Relevance/Pertinence:** It is critically important for the profession of physiotherapy to create a pool of physiotherapists with core leadership competencies. The Leadership Division would like to discuss transformational leadership as a key approach to assist aspiring physiotherapists in developing key leadership skills and in moving into leadership roles. This program will also help current physiotherapy leaders further develop their skills and thus meet their leadership challenges.

**Target Population/Population cible:** This session will be of interest to a broad range of professionals including clinicians, managers, professional leaders, educators, and researchers interested in developing or further developing leadership skills and the application of those skills to all areas of the health care system.

**Supporting Evidence/Résumé des faits:** Many professionals in the health care system are challenged to continue to grow and lead in the current realities of competing priorities and fiscal restraints. The profession of physiotherapy has made strong recent resources available to help all physiotherapists identify both roles and competencies for effective practice in Canada. The National Physiotherapy Advisory Group, and partners, published the Essential Competency Profile for Physiotherapists in Canada in October 2009. This profile describes the essential competencies required by physiotherapists in Canada at the beginning and throughout their career and provides guidance on how to build competencies over time. The framework for the Profile was adapted from the Royal College of Physicians and Surgeons of Canada Competency Framework for Physicians (the CanMEDS Roles). Throughout all seven roles for both professions, (Expert, Communicator, Collaborator, Manager, Advocate, Scholarly Practitioner and Professional) is the need for leadership knowledge, skills and behaviours. Leadership and management training are one way that can help support life-long learning for professionals as needed in the quickly changing nature of health care practice environments.

There are many current leadership theories and emerging cognitive constructs for leadership. Transformational leadership, where one engages followers to be leaders, is a methodology used in leadership education. Some theorists would suggest that leadership is not a moral or ethical question, but an early theorist, James McGregor Burns (1978) who created the Burns Transformational Leadership Theory describes this as when one or more people engage with others in such a way that leaders and followers raise one another to higher levels of motivations and morality. More recently Avolio (2009) outlines that transformational leadership is leader behaviours that transform and inspire followers to perform beyond expectations while transcending self-interest for the good of the organization.

In these rapidly changing and complex times, leaders need to be clear about what the ultimate goal of leadership is and why one should be a leader. The Leadership Division wants to help empower our profession with physiotherapy specific leadership knowledge, skills and behaviours.

**Session Format/formule de la session:** This session will be in a 120 minute workshop format. Participants will first learn, in lecture format, about the current state of leadership theories and highlight emerging cognitive constructs for leadership. Transformational leadership definitions from the literature will be discussed. Participants will then discuss cases of physiotherapy leadership scenarios in small groups and then share examples and applications to their own practice and settings. An interactive large group discussion will end the workshop where participants will be encouraged to gain insight into their own capacity to be a transformational leader.

**Keywords/Mots clés:** Leadership, Education

# Abstract: A003 – Effect of interdialytic fluid gain on muscle strength in people with end-stage kidney disease on haemodialysis: A pilot project

**Published:** 2013-05

**Authors:** A Sawant, DM Connelly, BM Chesworth, TJ Doherty, AA House, J Gati, R Bartha, TJ Overend

**Affiliations:** * Program in Health and Rehabilitation Sciences, Physical Therapy Field, Western University; Physiotherapist, London Health Sciences Center, 339 Windermere Rd, London, ON; † Associate Professor, School of Physical Therapy, Western University, London ON; ‡ Assistant Professor, School of Physical Therapy, Western University, London ON; § Associate Professor, Clinical Neurological Sciences & Rehabilitation Medicine, Western University London, ON; Chair, Physical Medicine and Rehabilitation, Parkwood Hospital, 801 Commissioners Rd E, London, Ontario, ON; ¶ Professor of Medicine, Associate Chair, Western University, Division of Nephrology, London Health Sciences Centre, 339 Windermere Rd, London, ON; ** Facility Manager, The Centre for Functional and Metabolic Mapping, Robarts Research Institute, 100 Perth Dr, London, ON; †† Scientist, Center for functional and Metabolic mapping, Robarts Research Institute; Associate Professor of Medical Biophysics, Western University, London, ON; ‡‡ Associate Professor and Director, School of Physical Therapy, Western University, London, ON

**Purpose & Objectives / Intention & objectifs:** Inconsistent findings in strength gains following exercise interventions across studies in people with end-stage kidney disease on haemodialysis have been related to multiple catabolic processes associated with haemodialysis and fluctuation in body hydration. The purpose of this study was to investigate the effects of interdialytic fluid on tibialis anterior strength in people with end- stage kidney disease on haemodialysis.

**Relevance/Pertinence:** Physiotherapy practice includes providing appropriate and effective exercise interventions to improve strength and function in people with haemodialysis.

**Materials & Methods/Matériel & méthods:** We recruited 17 participants into each, a control and an experimental, group. Intra and extra-cellular water in tibialis anterior was measured using segmental bioelectrical impedance analysis and magnetic resonance acquired T2 relaxation times before and after haemodialysis, and on two occasions for the control group. Peak isometric strength was measured using an isometric dynamometer.

**Analysis/Analyse:** The effect of haemodialysis on all outcomes between and within groups was compared using independent and paired sample t tests. Significance was assumed at p<0.05.

**Results/Résultats:** Mean differences between groups were significantly lower in haemodialysis group for tibialis anterior peak isometric strength by 9.15 and10.56 Nm, p<0.05, respectively and higher extra-cellular fluid by 9.04 and 6.80 msec, p<0.05, respectively. Within the haemodialysis group, mean differences after haemodialysis in peak isometric strength (-1.54 Nm, p<0.05), intracellular fluid (-0.05 liters, p<0.05) and extracellular fluid (-2.38 msec, p<0.05) were significant. Conclusions: Participants receiving haemodialysis had decreased strength following this treatment. This could be related to greater extracellular fluid and loss of intracellular fluid in the tibialis anterior muscle following haemodialysis.

**Keywords/Mots clés:** Haemodialysis, tibialis anterior, isometric strength, Bioelectrical impedance spectroscopy, T2 relaxation times.

# Abstract: A004 – Physiotherapists’ Perceptions and Experiences of the Discharge Planning Process in Acute Care General Medicine Units in Ontario

**Published:** 2013-05

**Authors:** L Matmari, J Uyeno, CS Heck

**Affiliations:** * Toronto Western Hospital, UHN, Allied Health, Physiotherapy, 3EW-408, 399 Bathurst St, Toronto, ON; † Scientist, Allied Health, University Health Network, 4E-306 Toronto General Hospital, 200 Elizabeth St, Toronto, ON

**Purpose & Objectives / Intention & objectifs:** To identify issues and factors considered by Ontario physiotherapists in discharge planning of acute care general internal medicine patients

**Relevance/Pertinence:** A key role for physiotherapists in acute care is the completion of discharge readiness assessments. Having a better understanding of physiotherapy-specific factors, barriers and facilitators may improve this process

**Materials & Methods/Matériel & méthods:** A quantitative cross-sectional study using an online questionnaire was sent to participants in November 2011

**Analysis/Analyse:** Subject’s demographic characteristics and ranking of factors were calculated using descriptive statistics. T-tests were performed to determine group differences by practice type, geographic location, and number of beds. Constant comparative analysis with coding of themes was used to analyze responses to open-ended questions

**Results/Résultats:** Responses were received from 55 physiotherapists (67.9 percent response rate). Mobility status was identified as the key factor in determining discharge readiness. Other factors considered included availability of social supports and community based healthcare resources. While interprofessional communication was identified as important, communication processes were often informal. Discharge policies, lack of other discharge options, and the pressure for early discharge impacted discharge planning. A lack of training regarding discharge planning was reported. Stories of ethical dilemmas experienced by respondents supported these themes. Conclusions: Physiotherapists consider many factors beyond the patient’s physical function during the discharge planning process. Improving team communication, timely patient and family education regarding discharge policies and the reality of community supports, and incorporating realities of discharge planning into the physiotherapy curriculum are some areas that should be considered to improve the process and the physiotherapists’ experience

**Keywords/Mots clés:** Discharge Planning, Acute Care, General Internal Medicine

# Abstract: A006 – Side Differences in Ground Reaction Forces During Walking and Sit-to-Stand Tasks in Individuals at Four Months Post Knee Arthroplasty: Preliminary Data

**Published:** 2013-05

**Authors:** S Nadeau, G Gagnon, H Moffet, M Tousignant

**Affiliations:** * Laboratoire de pathokinésiologie, Centre de recherche interdisciplinaire en réadaptation (CRIR), Institut de réadaptation Gingras-Lindsay-de-Montréal, Montréal, QC; † École de réadaptation, Université de Montréal, Montréal, QC; ‡ Centre interdisciplinaire de recherche en réadaptation et intégration sociale (CIRRIS), Institut de réadaptation en déficience physique de Québec and; § Département de réadaptation, Université Laval, Laval, QC; ¶ Centre de recherche sur le vieillissement Institut Universitaire CSSS-IUGS and; ** École de réadaptation, Université de Sherbrooke, Québec, QC

**Purpose & Objectives / Intention & objectifs:** We compared asymmetry of ground reaction forces under sit- to-stand and walking conditions in knee arthroplasty participants and determined impact of knee strength deficits.

**Relevance/Pertinence:** Rising from a chair and level walking are demanding tasks but essential to independent living. Performance data collected after knee surgery is relevant for guiding a home-based exercise program.

**Materials & Methods/Matériel & méthods:** Fourteen individuals who had received 8 weeks of physiotherapy participated in a study four months after undergoing knee arthroplasty. Impairments and function were assessed using a standard clinical evaluation. Participants walked at natural and fast speeds and rose from a chair using three foot positions: spontaneous, symmetrical and asymmetrical with the operated foot back. Ground reaction forces were recorded using force plates and compared between sides while walking and rising from a chair.

**Analysis/Analyse:** Asymmetry was compared between conditions and tasks using descriptive statistics and ANOVAs. Pearson coefficients were employed to assess the relationship between strength deficits and level of asymmetry.

**Results/Résultats:** During walking, the propulsive force differed between sides regardless of the walking speed. For the sit-to-stand tasks, participants had unequal weight-bearing distribution under the foot, although they were able to perform symmetrically when the foot position was modified. Overall, asymmetry is greater during sit-to-stand transfers than during walking. At four months, the remaining mean strength deficit is 30%. Weight-bearing asymmetry is highly associated with a bilateral knee strength difference. Conclusions: At four months post surgery, the knee arthroplasty participants did not perform the functional task symmetrically. Physiotherapists should provide this population with specific exercises for strengthening the knee muscles.

**Keywords/Mots clés:** Asymmetry, Knee Arthroplasty, Sit-to-stand Transfer, Level walking, Strength

# Abstract: A007 – Trunk muscle temporal patterns remain altered in a sub-acute low back injured population despite subjective reports of recovery

**Published:** 2013-05

**Authors:** J Moreside, A Quirk, C Hubley-Kozey

**Affiliations:** * Schools of Health and Human Performance and; † Physiotherapy, Dalhousie University, Halifax, NS

**Purpose & Objectives / Intention & objectifs:** To compare trunk muscle electromyographic (EMG) patterns between 2 groups during a controlled exercise: 1) those with recent (≤12 weeks) low back injury (LBI), now deemed recovered and pain-free; 2) an asymptomatic group (ASYM) with no similar injury history.

**Relevance/Pertinence:** Altered temporal and amplitude trunk muscle patterns have been identified during acute or chronic low back pain episodes, but little is known about motor changes during the sub-acute phase of recovery. Such metrics are required to objectively determine work readiness.

**Materials & Methods/Matériel & méthods:** In this cross-sectional comparison study, eighty-one (30-LBI, 51- ASYM) volunteers performed a supine trunk/pelvis stability test (two levels of difficulty, 3X each). EMG data from 24 trunk muscles were full wave rectified, low pass filtered, time normalized to 100% and amplitude normalized to maximum voluntary contractions.

Principal component (PC) analysis was used to objectively quantify temporal and amplitude EMG patterns.

**Analysis/Analyse:** EMG ensemble average waveforms for all participants were entered into 2 PC analysis models (abdominals and back separately). Resulting PC scores explaining 90% of the variance were entered into a mixed model ANOVA.

**Results/Résultats:** There were significant group (ASYM vs LBI) effects for PCs 1and 4(p<0.05). LBIs demonstrated higher overall amplitudes. Group*muscle interactions highlighted altered temporal patterns in PCs 2,3 consistent with more continuous activation patterns, less ability to fine tune muscles according to the applied moment. **Conclusions:** Despite subjective reports of recovery, objective measures showed temporal and amplitude EMG characteristics remain altered during the sub-acute recovery phase. This highlights the importance of objective metrics rather than subjective report when determining work readiness.

**Keywords/Mots clés:** electromyography, sub-acute low back pain, principal component analysis, trunk stability test

# Abstract: A009 – What can we learn from the ‘liberation’ procedure phenomenon? A qualitative study among people with multiple sclerosis

**Published:** 2013-05

**Authors:** M Ploughman, C Murray, M Murdoch, C Harris, S Hogan, M Stefanelli

**Affiliations:** * Eastern Health Authority and Faculty of Medicine, Memorial University, St. John’s, NL; † Faculty of Nursing, Memorial University, St. John’s, NL; ‡ Coalition of Persons with Disabilities, St. John’s, NL

**Purpose & Objectives / Intention & objectifs:** Many people with multiple sclerosis (MS), upon hearing of the research findings by Dr. P. Zamboni and anecdotal evidence, have strongly endorsed the ‘liberation’ procedure, often placing them at odds with health care and research communities. We explored how people with MS made decisions when considering the controversial ‘liberation’ procedure as a treatment for MS.

**Relevance/Pertinence:** Physiotherapists find themselves conflicted between evidence-based practice and patient demands. Understanding how critical health decisions are made helps place these conflicts in perspective.

**Materials & Methods/Matériel & méthods:** Fifteen participants from a local MS clinic (8 who had the procedure within the previous 6 weeks and 7 who had decided against having the procedure) participated in face-to-face open-ended semi-structured audiotaped interviews.

**Analysis/Analyse:** Transcribed text was independently coded by three investigators experienced in content analysis. We modified the coding scheme through an iterative consensus-based approach. Themes and subthemes were interpreted and described.

**Results/Résultats:** Participants highly valued immediate communication with trusted sources. Trust was proximity dependent; meaning that participants placed trust in advisors who were perceived to be intimately impacted by the health decision (other people with MS, family and friends). Decisions were also influenced by the participant’s perception of their disease progression (desperation), hope, community fundraising and educational background. Conclusions: The ‘liberation’ procedure has highlighted a new health information and decision-making reality. The health care consumer weighed the ‘evidence’ gleaned from multiple non- traditional sources and placed trust on immediate communication from those more intimate with the potential outcome.

**Keywords/Mots clés:** CCSVI, multiple sclerosis, qualitative, Zamboni

# Abstract: A010 – Assessing NeuroFIT, a community-based exercise program for people with moderate to severe neurological disabilities

**Published:** 2013-05

**Authors:** J Shears, M Ploughman, C Harris, S Hogan, S Squires, J McCarthy

**Affiliations:** * Eastern Health Authority and Faculty of Medicine, Memorial University, St. John’s, NL; † Ches Penney Family Y, St. John’s, NL

**Purpose & Objectives / Intention & objectifs:** We partnered with a community organization (YM/YWCA) to develop, implement and test a community exercise program to improve participation among people who rarely progress their mobility outside their homes or participate in former life roles.

**Relevance/Pertinence:** Physiotherapists are challenged to find safe community programs for people who are not yet community ambulators.

**Materials & Methods/Matériel & méthods:** Participants were recruited following discharge from an outpatient rehabilitation service. Inclusion criteria included: neurological diagnosis, able to walk at least 3m with assistance and walking aid and be evaluated by a doctor. The program, modified from other programs and supervised by a physiotherapist, ran 2Xwk for 10 weeks and consisted of progressively challenging functional circuit activities. Participants (10 per group) were also instructed on the use of gym equipment. We recruited and trained students to provide 1:1 assistance. Participants’ mobility (Timed Up and Go), mood (Hospital Anxiety and Depression Scale), participation (Frenchay Activities Index) and quality of life (QoL) were measured before and after the program and again at 4 month follow-up.

**Analysis/Analyse:** We used a pre-posttest design with follow-up. Since a power analysis was not completed, the outcomes presented are descriptive.

**Results/Résultats:** The 29 participants (stroke n=18, ABI n= 7, Parkinson’s n=2 MS n=2) made improvements in mobility (4.8±8.9s Timed Up and Go). They increased participation (2.6/45±3.9 Frenchay) and reported better QoL (14.5/100±26) and mood (-2.6/42±5.9 HADS) which was maintained at follow-up. Oversight by a physiotherapist was required for safety. Conclusions: This carefully-designed, supervised and physiotherapist-monitored community exercise program improved participation, mood and QoL among persons with moderate to severe neurological disability

**Keywords/Mots clés:** community, participation, brain injury

# Abstract: A011 – Development of a clinical prediction rule to identify patients at risk of poor outcomes following total knee arthroplasty

**Published:** 2013-05

**Authors:** E Lungu, F Desmeules, CE Dionne, EL Belzile, PA Vendittoli

**Affiliations:** * Maisonneuve-Rosemont Hospital Research Center, University of Montreal Affiliated Research Center, Montreal, QC; † Biomedical sciences, Faculty of Medicine, University of Montreal, Montreal, QC; ‡ School of Rehabilitation, Faculty of Medicine, University of Montreal, Montreal, QC; § URESP du Centre de recherche FRSQ du CHA Universitaire de Québec, Quebec City, QC; ¶ Department of Rehabilitation, Faculty of Medicine, Université Laval, Quebec City, QC; ** Laval University Hospital Center (CHUQ), Quebec City, QC; †† Departement of Surgery, Faculty of Medicine, University of Montreal, Montreal, QC

**Purpose & Objectives / Intention & objectifs:** To develop a clinical prediction rule (CPR) to identify patients enrolled on presurgical wait lists who are at the greatest risk of poor outcomes 6 months after total knee arthroplasty.

**Relevance/Pertinence:** TKA is the recommended treatment for patients with severe forms of arthritis, but as much as one third of patients reports poor outcomes. Identification of these patients before the intervention would allow for their better selection and preparation and, eventually, improved outcomes.

**Materials & Methods/Matériel & méthods:** 141 patients scheduled for TKA were recruited from the wait lists of 3 hospitals in Quebec City, Canada. Knee pain, stiffness and function were measured 6 months after TKA using the Western Ontario and McMaster Osteoarthritis Index (WOMAC) and participants in the lowest quintile for the WOMAC total score were considered to have poor outcomes. Demographic, socioeconomic, psychosocial, clinical factors and the WOMAC score at enrolment on the wait lists (baseline) were considered potential predictors.

**Analysis/Analyse:** The prediction rule was built with recursive partitioning.

**Results/Résultats:** The best prediction was provided by 5 items of the baseline WOMAC score (taking off socks, getting on/off toilet, performing light domestic duties, rising from bed, and stiffness after the first wakening in the morning). The rule had a sensitivity of 82.1% (95%CI: 66.7-95.8), a specificity of 71.7% (95%CI: 62.8-79.8), and positive and negative likelihood ratios of 2.9 (95%CI: 1.8-4.7) and 0.3 (95%CI: 0.1-0.6) respectively. Conclusions: The CPR developed in this study is a promising tool to identify patients at risk of worse outcomes 6 months after TKA and could improve the management of these patients.

**Keywords/Mots clés:** Osteoarthritis, disability, Total knee arthroplasty, clinical prediction rule, validation

# Abstract: A016 – Effectiveness of In-Home Telerehabilitation on functional ability and knee mobility after a first Total Knee Arthroplasty

**Published:** 2013-05

**Authors:** H Moffet, M Tousignant, S Nadeau, C Mérette, P Boissy, H Corriveau, F Marquis, F Cabana, P Ranger, E Belzile, P Larochelle, R Dimentberg

**Affiliations:** * Université Laval, Quebec, QC; † Centre for Interdisciplinary Research and Social Integration, Quebec, QC; ‡ Université de Sherbrooke and Research Center on Aging, Sherbrooke, QC; § Université de Montréal and Centre for Interdisciplinary Research in Rehabilitation of Greater Montreal, Montreal, QC; ¶ Centre de recherche de l’Institut universitaire en santé mentale, Quebec, QC; ** CHUQ, Quebec, QC; †† CHUS, Sherbrooke, QC; ‡‡ Hôpital Jean-Talon, Montreal, QC; §§ CSSS-Arthabaska-Les-Érables, Arthabaska, QC; ¶¶ McGill University and St. Mary’s Hospital, Montreal, QC

**Purpose & Objectives / Intention & objectifs:** To determine if an in-home telerehabilitation (TR) approach is as effective as a face-to-face home visit approach (Control; CTL) after hospital discharge in persons with total knee arthroplasty (TKA).

**Relevance/Pertinence:** Finding a cost-effective alternative to home physiotherapy visits for post-surgical rehabilitation is a major issue considering the increasing need for home care services and shortage of health resources.

**Materials & Methods/Matériel & méthods:** 205 persons were randomly assigned to the TR or CTL group. Both groups received the same rehabilitation intervention over the 2 first months after hospital discharge. Participants were evaluated 4 times: before TKA (E1), at discharge (E2), 2 months (E3) and 4 months (E4; 2 months post-intervention) post-discharge. The primary outcome measure was the WOMAC questionnaire at E4. Secondary measures included: Six-minute walk test and Range of knee motion.

**Analysis/Analyse:** A per-protocol analysis was performed (main hypothesis tested: H0: μCTL-μTR≥ 9%).

**Results/Résultats:** 177 subjects were considered in the per-protocol analysis (TR, n=82; CTL, n=95). Subjects of both groups had similar characteristics at baseline. WOMAC gains at E4 did not differ between groups. Mean differences between groups (CTL-TR) adjusted for E1 [95% confidence intervals] were close to zero and, on average, slightly favored the TR group: Total: -1.48% [-5.51, 2.54]; Pain: -1.67% [-6.02, 2.68]; Stiffness: 0.03% [-6.25, 6.32]; Function: -1.65% [-5.79, 2.49] and confidence intervals were all within 9%. No significant difference between groups was found in secondary measures at E4. **Conclusions:** Our results support the non-inferiority of the in-home TR approach and its consideration as an effective alternative to conventional physiotherapy service delivery.

**Keywords/Mots clés:** Telerehabilitation, Clinical Trial, Arthroplasty

# Abstract: A017 – Cumulative Occupational Physical Load as a Risk Factor for Knee Osteoarthritis

**Published:** 2013-05

**Authors:** AM Ezzat, J Cibere, M Koehoorn, EC Sayre, LC Li

**Affiliations:** * University of British Columbia, Vancouver, BC; † Arthritis Research Centre of Canada, Vancouver, BC; ‡ Child and Family Research Institute, Vancouver, BC

**Purpose & Objectives / Intention & objectifs:** To determine the association between cumulative occupational physical load (COPL) and knee osteoarthritis (OA), defined as Symptomatic Radiographic Osteoarthritis (SOA) or Magnetic Resonance Imaging Osteoarthritis (MRI-OA).

**Relevance/Pertinence:** Understanding the complete risk profile for patients, including work risk factors, can help physiotherapists with education and prevention of knee OA in the population.

**Materials & Methods/Matériel & méthods:** This was a cross-sectional analysis of symptomatic and asymptomatic knee cohorts. Participants received a knee exam, knee radiographs and MRI, and completed a questionnaire, which included occupational history that was used to calculate quartiles (QCOPL). SOA was defined by the Kellgren Lawrence x-ray grade > 2, plus knee pain. MRI-OA was defined using a recently published novel definition.

**Analysis/Analyse:** Weighted logistic regression examined the association between QCOPL and the presence of SOA and MRI-OA, after adjusting for age, sex, body mass index, and interactions.

**Results/Résultats:** Participants were on average 58.5(SD=11.0) years old with a BMI of 26.3(SD=4.7). A monotonic statistically significant relationship was found between QCOPL and SOA with adjusted odds ratio (OR) of 8.16(95%CI=1.89,35.27) for QCOPL 4 (highest) vs. QCOPL 1 (lowest), and 5.73(95%CI=1.36,24.12) for QCOPL 3 vs. 1. Adjusted OR for MRI-OA were also monotonic and statistically significant: QCOPL 4 vs. 1 (OR=9.54; 95%CI=2.65,34.27); QCOPL 3 vs. 1 (OR=9.04;95%CI=2.65,30.88); QCOPL 2 vs. 1 (OR=7.18;95%CI=2.17,23.70). Conclusions: Occupational loading is a significant risk factor for knee OA. A dose response relationship between COPL and both SOA and MRI-OA was found. Public education on the role of occupation in knee OA is warranted.

**Keywords/Mots clés:** Occupation, Osteoarthritis, risk factor

# Abstract: A018 – Neuromuscular partitioning of supraspinatus: Implications for rotator cuff pathology and rehabilitation

**Published:** 2013-05

**Authors:** J Hermenegildo, SY Kim

**Affiliations:** Division of Anatomy, Department of Surgery, Faculty of Medicine, University of Toronto, Toronto, ON; School of Physical Therapy, College of Medicine, University of Saskatchewan, Saskatoon, SK

**Purpose & Objectives / Intention & objectifs:** To confirm the presence of neuromuscular partitions within the supraspinatus muscle by investigating the intramuscular innervation pattern of the suprascapular nerve (SSN).

**Relevance/Pertinence:** The supraspinatus is composed of anterior and posterior regions. Muscle fiber type compositions and dynamic assessment of fiber bundle changes suggests the anterior and posterior regions are distinct neuromuscular partitions. By investigating the intramuscular innervation pattern of supraspinatus we can further confirm this hypothesis which can have great implications for understanding and treating rotator cuff pathology.

**Materials & Methods/Matériel & méthods:** The supraspinatus muscle was exposed in ten formalin embalmed specimens without any tendon pathology. The SSN at the suprascapular notch was identified and digitized sequentially throughout the muscle volume using Microscribe™ G2X digitizer. The digitized data was modelled using Maya®.

**Analysis/Analyse:** The three-dimensional computer models were used to document the innervation pattern and to relate branches to distinct regions of the muscle.

**Results/Résultats:** The SSN has two primary motor branches that innervate the supraspinatus: anterior and posterior. The anterior branch further subdivides into two branches: medial and deep. The anterior region is innervated by the anterior branch and its medial branch. The posterior region has duel innervation, where the lateral aspect is innervated by the posterior branch and the medial aspect by the deep branch of the anterior. Conclusions: This is the first study to confirm the presence of two neuromuscular partitions within supraspinatus. The location of the posterior branch makes it vulnerable to damage with cuff tears and tendon repair surgery. Function of the normal and pathologic supraspinatus along with rehabilitative exercises need to be re-evaluated with this new insight.

**Keywords/Mots clés:** supraspinatus, suprascapular nerve, neuromuscular partitions, computer modelling, rotator cuff

# Abstract: A019 – Randomised Clinical Trial of At-Arthroscopy Viscosupplementation (hylan G-F 20) for Post- operative Pain and Function: A Pilot Study

**Published:** 2013-05

**Authors:** LA Hiemstra, A Kerslake, SM Heard, GML Buchko

**Affiliations:** * Banff Sport Medicine, Banff, AB; † Department of Surgery University of Calgary, Calgary, AB; ‡ Department of Physical Therapy, University of Alberta, Edmonton, AB

**Purpose & Objectives / Intention & objectifs:** The primary purpose of this pilot study was to determine the feasibility of conducting a randomised clinical trial of at-arthroscopy viscosupplementation following arthroscopic meniscectomy.

**Relevance/Pertinence:** Reducing time to return to activities following knee arthroscopy is a primary goal of patients and therapists.

**Materials & Methods/Matériel & méthods:** Thirty male subjects with a confirmed diagnosis of medial meniscus tear and mild osteoarthritis were randomly assigned to one of 2 treatment groups following knee arthroscopy for partial medial meniscectomy. Group one received a 6ml viscosupplementation injection (hylan G-F 20), and group two received a 6ml placebo injection (saline). Patients were assessed at 1,6 and 12 weeks post-operative by a physiotherapist via a standard orthopaedic clinical examination, the Western Ontario Meniscal Evaluation Tool (WOMET), the Knee Osteoarthritis Outcome Score (KOOS), and a 100mm visual analog scale (VAS) pain rating.

**Analysis/Analyse:** Independent t tests were used to determine if meaningful differences in pain and function existed between the groups.

**Results/Résultats:** The viscosupplementation group demonstrated lower mean VAS pain ratings than the placebo group at 6-weeks (5.86 v 17.86) and 12-weeks (2.62 v 14.36) post-operative. Independent t tests demonstrated a significant between groups difference in VAS pain rating at 12-weeks, p = 0.029. Data trends indicated the viscosupplementation group achieved greater improvement in WOMET (p = 0.13), KOOS function (p = 0.042) and KOOS sports (p = 0.11) domain scores at 12-weeks post-operative. Conclusions: These pilot study results indicate the feasibility of conducting an RCT investigating an at-arthroscopy viscosupplementation injection (hylan G-F 20) to improve pain and function following partial meniscectomy.

**Keywords/Mots clés:** Meniscectomy, Viscosupplementation, Pain, Function, Knee

# Abstract: A020 – Music Supported Therapy To Improve Upper Extremity Function After Stroke: A Pilot Study

**Published:** 2013-05

**Authors:** M Villeneuve, A Lamontagne

**Affiliations:** McGill University, School of Physical and Occupational Therapy, Montreal, QC

**Purpose & Objectives / Intention & objectifs:** Previous studies have shown that piano training can induce improvements in fine and gross motor skills in acute stroke survivors. However, whether such intervention can be used in chronic stroke survivors and lead to longer-term improvements remain unknown. The purpose of this study was to estimate the short and long-term effects of a 3-week piano training program on upper extremity (UE) function in persons with chronic stroke.

**Relevance/Pertinence:** This intervention could be used as a rehabilitation resource-effective adjunct therapy.

**Materials & Methods/Matériel & méthods:** Four subjects with a chronic (6–24 mo) stroke participated in a piano training of 9 sessions (60min) in addition to a home program including piano exercises. A MIDI-piano program was used to teach songs involving all 5 digits of the paretic hand. As the participant progressed, the song’s frequency, complexity (finger sequences) and duration increased. Manual dexterity and UE function were assessed pre-, post-intervention and at 3-week follow-up using the Nine Hole Peg Test (NHPT), the Box and Block (B&B) and the Jebsen.

**Analysis/Analyse:** Descriptive statistics were used to quantify changes post-intervention and at follow-up.

**Results/Résultats:** Fine and gross manual dexterity improved, as shown by a mean reduction of 24.79s on the NHPT and a mean increase of 6 blocks on the B&B. Increments ranging from 24% to 51% were observed on all subtests of the Jebsen, indicating improvements in the functional use of the paretic UE. Changes were maintained at the 3-week follow-up. Conclusions: These findings support the feasibility of using music- supported therapy to improve UE function in chronic stroke survivors.

**Keywords/Mots clés:** Neurology, Stroke, Music, Rehabilitation, Plasticity, Motor Learning

# Abstract: A021 – Cognitive status and use of feedback for arm motor recovery in chronic stroke

**Published:** 2013-05

**Authors:** SK Subramanian, G Chilingaryan, H Sveistrup, MF Levin

**Affiliations:** * School of Physical and Occupational Therapy, McGill University, Montreal, QC; † Department of Social and Preventive Medicine, Université de Montréal, Montreal, QC; ‡ School of Rehabilitation Sciences, University of Ottawa, Ottawa, ON; § Feil and Oberfeld Research Centre, Jewish Rehabilitation Hospital site of Center for Interdisciplinary Research in Rehabilitation (CRIR), Laval, QC

**Purpose & Objectives / Intention & objectifs:** Along with sensorimotor impairments, stroke survivors (~65%) have cognitive impairments (depression, memory, attention, mental flexibility, problem-solving and visuoperception) which can influence motor learning. Explicit task performance feedback may improve learning with the impaired arm. However, whether cognitive impairments limit the ability to use explicit feedback for motor recovery remains unclear. We evaluated the association between these factors.

**Relevance/Pertinence:** Results will inform neurological physical therapy practice about which post-stroke patients may benefit from feedback provision to enhance motor learning.

**Materials & Methods/Matériel & méthods:** Participants (n=24; 61.2±10yrs) practiced pointing movements in a random sequence 72times/session to 6 targets for 4wks (12trials/target; 3sessions/wk).Terminal auditory feedback was provided about movement speed and trunk displacement. Changes in movement kinematics (speed, trunk, shoulder horizontal adduction (ShHor) and flexion (ShFl) movement) were assessed after practice (post) and 3 months later (follow-up). Depression and cognitive functioning were assessed.

**Analysis/Analyse:** Multiple regression analyses determined the relationship between cognitive scores (predictors) and motor improvements (dependent variables).

**Results/Résultats:** Greater endpoint speed was associated with better memory and problem solving ability post-practice (26%) and at follow-up (42%). Depression and memory explained changes in trunk displacement post-practice (28%) and mental flexibility, attention and visuoperception explained changes at follow-up (50%). Increased ShHor range post-practice was explained by mental flexibility and attention (25%). For ShFl, improvements post-practice were associated with better memory and mental flexibility (37%) and with problem-solving ability and visuoperception at follow-up (25%). Conclusions: Cognitive functioning levels were associated with the ability to use feedback. Information about cognitive deficits can help select appropriate interventions to maximize arm motor recovery post-stroke.

**Keywords/Mots clés:** feedback, recovery, rehabilitation, kinematics, upper-limb

# Abstract: A022 – A Model of Community Ambulation after Stroke – a Mixed-Methods Study

**Published:** 2013-05

**Authors:** R Barclay-Goddard, J Ripat

**Affiliations:** * School of Medical Rehabilitation, University of Manitoba, Winnipeg, MB; † Department of Physical Therapy; ‡ Department of Occupational Therapy

**Purpose & Objectives / Intention & objectifs:** Objectives were to: Develop a model of community ambulation after stroke; verify the model and explore community ambulation from the perspective of individuals with stroke.

**Relevance/Pertinence:** Community ambulation is an important rehabilitation outcome for many individuals with stroke.

**Materials & Methods/Matériel & méthods:** An explanatory sequential mixed-methods design (quantitative before qualitative) was used. Secondary data analysis of the “Getting on with the Rest of Your Life after Stroke” trial was conducted to develop the model. Two focus groups of individuals with stroke were conducted to verify and explain the model.

**Analysis/Analyse:** Structural equation modeling was used to develop measurement and structural models from baseline evaluations. A content analysis approach was used to analyse focus group transcripts.

**Results/Résultats:** Data from 226 participants contributed to the model: 142(63%) were male; age was 63.4(12.0) years; years since stroke 2.6(2.5). Eleven participated in the focus groups: 6(55%) were male; age 61.4(6.9) years; years since stroke 5.8(3.3).The structural model had a reasonable fit with three latent variables: mobility, gait speed, and health perceptions (normed χ2 = 1.8, RMSEA=0.060(0.043; 0.075)). Health perceptions and gait speed may help to explain mobility. Depression may help explain health perceptions, indirectly explaining mobility. Participants verified the model, emphasizing the importance of physical and social environment considerations that influence community ambulation. **Conclusions:** A model of community ambulation after stroke was developed and verified by individuals living with stroke.Identifying important aspects of community ambulation may assist physiotherapists in determining community ambulation goals, needs, and opportunities in collaboration with clients.

**Keywords/Mots clés:** community, ambulation

# Abstract: A023 – Factors That Influence Length of Stay in an Out-patient Rehabilitation Center for Patients with Stroke

**Published:** 2013-05

**Authors:** M Gandhi, N Karunaratne, R Vaccariello, Y Zhao, I Hamel-Hébert, M-J Malo, J Spahija

**Affiliations:** * School of Physical and Occupational Therapy, McGill University, Montreal, QC; † Constance Lethbridge Rehabilitation Hospital, Centers for Interdisciplinary Research in Rehabilitation (CRIR), Montreal, QC; ‡ Feil/Oberfeld Research Centre, Jewish Rehabilitation Hospital site of Center for Interdisciplinary Research in Rehabilitation (CRIR), Laval, QC; § Hôpital du Sacré-Coeur de Montréal, Université de Montréal, Montreal, QC

**Purpose & Objectives / Intention & objectifs:** Studies have looked at length of stay in in-patient rehabilitation, but no existing study has investigated the factors pertinent to an out-patient center. This study aimed to determine the factors that influence length of stay for patients with stroke at an out-patient phase-three rehabilitation center.

**Relevance/Pertinence:** Identifying the factors that influence length of stay can provide insight into treatment needs and facilitate better allocation and management of rehabilitation resources.

**Materials & Methods/Matériel & méthods:** A literature search and focus group meeting with professionals were performed to identify factors considered as important contributors to length of stay. Fourteen factors were retained for study. A retrospective chart review from individuals followed post-stroke at the center from June 2009 to May 2012 was performed.

**Analysis/Analyse:** Univariate and multiple regression analyses were used to identify the main predictors of length of stay.

**Results/Résultats:** From 115 charts reviewed, 86 fulfilled inclusion/exclusion criteria. Median length of stay was 250 days (mean, 263 days; range, 70 -763 days). Significant relationships were identified for 1) number of functional problems on admission, 2) number of goals set for treatment, 3) number of professional disciplines following patients, and 4) age. The most significant independent predictors were number of goals set for treatment and age, with the fitted model explaining 30% of the variance in the length of stay.

**Conclusions:** The results show that individuals who are younger and who set more goals with their therapists tend to stay longer in out-patient rehabilitation. Identifying these individuals at risk may enable clinicians to offer more individualized care and strategies for optimized management.

**Keywords/Mots clés:** stroke, rehabilitation, length of stay, out-patient

# Abstract: A024 – Attitudes, willingness and preparedness towards adults with intellectual disability: A survey of Ontario Occupational and Physical Therapy students

**Published:** 2013-05

**Authors:** K Vermeltfoort, A Staruszkiewicz, K Anselm, A Badnjevic, K Burton, R Balogh, E Yeung, S Switzer-McIntyre

**Affiliations:** University of Toronto Department of Physical Therapy, 500 University Ave, Toronto ON

**Purpose & Objectives / Intention & objectifs:** To examine attitudes, willingness, and preparedness of Ontario Masters Occupational Therapy (MScOT) and Physical Therapy (MScPT) students towards adults with intellectual disability (ID).

**Relevance/Pertinence:** This study addresses gaps in our understanding of rehabilitation students’ attitudes towards adults with ID. The results could inform curricular reform in the rehabilitation professions so that future clinicians are better prepared to provide support for this population.

**Materials & Methods/Matériel & méthods:** A quantitative cross-sectional survey study was conducted. An electronic questionnaire was distributed to 1255 MScOT/PT students at five Ontario universities via email, using a modified Dillman approach.

**Analysis/Analyse:** Descriptive statistics were conducted to describe experiences, attitudes, willingness and preparedness. Mann-Whitney U tests were used to find associations between attitudes and experience, attitudes and willingness, and attitudes and preparedness.

**Results/Résultats:** Overall response rate was 17.9%. A total of 96.0% of respondents felt “quite” or “very willing” to deliver rehabilitation to adults with ID. However, 50.7% of respondents felt “not at all” or “a little prepared” to interact with this population in a clinical setting. Of those who felt unprepared, 75.4% reported it to be due to inadequate knowledge. There was a significant association between cognitive attitudes and willingness to provide rehabilitation (p = 0.037). Additionally, Ontario MSc PT/OT students have neutral attitudes towards adults with ID. Conclusions: This study indicates that many MScOT/PT students are willing to deliver rehabilitation to adults with ID, however, a large percentage do not feel adequately prepared to interact with this population in a clinical setting.

**Keywords/Mots clés:** intellectual disability, physical therapy, occupational therapy, students, attitudes, preparedness, willingness

# Abstract: A026 – Contributors to the decision to supervise a physiotherapy student: Results from a national survey

**Published:** 2013-05

**Authors:** M Hall, C Poth, P Manns, L Beaupre

**Affiliations:** * Department of Physical Therapy; † Department of Educational Psychology, University of Alberta, Edmonton, AB

**Purpose & Objectives / Intention & objectifs:** To identify factors influencing Canadian physiotherapists’ decision to supervise physiotherapy students, and provide physiotherapy education stakeholders with a framework to address placement shortages.

**Relevance/Pertinence:** Clinical education is a critical component of physiotherapy training; however clinical coordinators report increasing difficulty in securing sufficient, appropriate placements. Evidence of the benefits and barriers for student supervision has been reported in a limited capacity, but factors contributing to Canadian physiotherapists’ decisions to supervise physiotherapy students have not been explored nationally.

**Materials & Methods/Matériel & méthods:** A web-based, 53 closed-item survey was developed with experts in clinical education using standard survey development methodology. All practicing Canadian physiotherapists were invited to participate through provincial regulatory colleges’ list-serves during May,2012.

**Analysis/Analyse:** Exploratory factor analysis was conducted using principal axis extraction and direct oblimin transformation performed to assess reliability of factor structure. Subsequent analysis compared factor scores between supervising and non-supervising physiotherapists and across geographic regions.

**Results/Résultats:** Analysis from a representative sample of 3148 (17%) physiotherapists suggests a six- factor structure contributing to the decision to supervise students: Clinical Instructor Stress, Efficiency, CPI, Preparation to Evaluate, Student, and Professional Role & Responsibility; findings consistent with recent studies. Significant differences were found between supervisors and non-supervisors and across geographic regions related specifically to Clinical Instructor Stress, Preparation to Evaluate, and Professional Role and Responsibility (p < 0.001). Conclusions: The decision to supervise students is multifactorial, complex, and includes factors outside the control of physiotherapy schools. Successful clinical education requires commitments from schools, employers and the profession.

**Keywords/Mots clés:** Clinical Education, Barriers, Factor analysis, Survey

# Abstract: A033 – Kinesiotape: Does it have an effect beyond stretching the skin? A pilot study investigating its influence on motor excitability

**Published:** 2013-05

**Authors:** SL Karam, F Tremblay

**Affiliations:** University of Ottawa, School of Rehabilitation Sciences, Guindon Hall, 451 Smyth Road, Room 3071, Ottawa, ON

**Purpose & Objectives / Intention & objectifs:** Kinesiotape has been increasing in popularity worldwide in the treatment and prevention of musculoskeletal injuries.

**Relevance/Pertinence:** Kinesiotape is applied in clinical practice and used by various athletes, despite the lack of evidence supporting its benefits.

**Materials & Methods/Matériel & méthods:** Ten healthy females aged 19–26 were recruited. Kinesiotape was applied over tibialis anterior(TA) and calf muscles. Transcranial magnetic stimulation(TMS) was used to evaluate changes in cortico-motor excitability related to Kinesiotape. Motor evoked potentials(MEPs) were evoked at rest and at 750ms/1500ms intervals during ankle dorsiflexion/plantarflexion movements with/without KT. Condition order was counterbalanced between participants;TMS delivery time was randomized. Three series of resisted dorsiflexion/plantarflexion contractions were performed as an index of MVC level. MEP amplitude/silent period(SP) means were obtained for the TA and soleus(SOL);average EMG amplitudes were recorded.

**Analysis/Analyse:** A repeated-measures ANOVA(2x2x2) was performed to examine the main effects of tape condition, movement direction, and stimulus time on MEP amplitude(μV) and silent period(ms) for each muscle (p<0.05). A 2x2-ANOVA was executed to examine effects of taping condition and stimulus time on average background-EMG level.

**Results/Résultats:** The application of Kinesiotape had no significant effect on TA(F=0.9,p=0.77) or SOL(F=0.1,p=0.98) MEP amplitude. There was no effect on TA MEP SP(F=0.15,p=0.71) or on EMG levels(F<1.5,p>0.22). Conclusions: Our findings do not support the claims that proprioceptive tape can translate into motor performance gains through an increase in afferent stimulation to the skin. Evidence supporting the application of Kinesiotape in the management of musculoskeletal injuries is inconclusive. Further studies are warranted given its widespread use in clinical practice and sports all over the world.

**Keywords/Mots clés:**

# Abstract: A034 – Canadian physiotherapy management after lower limb botulinum toxin injections for children with cerebral palsy

**Published:** 2013-05

**Authors:** L Benard, B Ramage, S Leew, S Goldstein

**Affiliations:** Neurosciences, Alberta Children’s Hospital, Calgary AB, Faculty of Medicine, University of Calgary, Calgary, AB

**Purpose & Objectives / Intention & objectifs:** Describe current PT practice strategies across Canada for children with CP after BtA injection.

**Relevance/Pertinence:** Botulinum toxin (BtA) injection is a common treatment for children with cerebral palsy (CP), and physiotherapy (PT) is frequently recommended after injection. The frequency, duration, and specific PT interventions are not well-defined in the literature and decisions about PT service delivery are unclear.

**Materials & Methods/Matériel & méthods:** We developed and validated a questionnaire for this cross- sectional survey. The survey link was sent to a purposeful sample of 31 PTs in Canada who work with children with CP followed by snowball sampling via email. The survey was open from June 2011 to October 2011.

**Analysis/Analyse:** Comparison between regions, age of child, functional level of the child and types of goals was completed using percentages and number of total responses.

**Results/Résultats:** 56 responses were received across all 10 provinces. Differences found in the amount of therapy provided related to the functional level of the child, and the goals of the intervention. Differences in reported treatment strategies were seen relative to the functional level of the child. Few differences in reported treatment were related to the age of the child or to the geographical location. Conclusions: Treatment varied depending upon the identified goals and the functional level of the child. Early goal identification and setting with the child and family should be used to guide treatment options. Further studies into outcomes of PT treatment after BtA injection are needed to determine how different therapy protocols impact pre-set functional goals.

**Keywords/Mots clés:** Cerebral Palsy, Botulinum Toxin type A, Physiotherapy

# Abstract: A036 – Effects of head flexion position on rate of isometric muscle force development in minor hockey players

**Published:** 2013-05

**Authors:** L Pelland, I Gilchrist

**Affiliations:** Queen’s University, School of Rehabilitation Therapy, Kingston, ON

**Purpose & Objectives / Intention & objectifs:** To quantify the effects of static head flexion posture on the rate of isometric force development (RFD) of neck musculature in male hockey players, 8 to 12 years old. Relevance/Pertinence: RFD of neck musculature has been postulated to dampen the magnitude of linear and rotational acceleration of the head following a force application. Understanding how RFD is modulated by the direction of force application and head posture is fundamental to designing effective concussion risk management strategies in minor hockey.

**Materials & Methods/Matériel & méthods:** Subjects were male hockey players, 8 to 12 years old. RFD (N/ms) was measured by linear load cell (2048 Hz) using a modified MCU dynamometer (BTE Technologies). Subjects performed maximal voluntary isometric contractions (MVICs) along flexion/extension and lateral bending axes of neck motion. Subjects performed three trials in each direction in head neutral and 20deg of flexion postures.

**Analysis/Analyse:** Effects of force direction and head posture were evaluated by repeated measures ANOVA.

**Results/Résultats:** RFD was independently modulated by direction of force (P=0.05) and head posture (P=0.03). In neutral, RFD was facilitated in extension (P≤ 0.02; 153.5 (39.2) N/ms extension; 80.4 (26.4) N/ms flexion; 60 (9.6) N/ms lateral bending). Head flexion posture increased RFD by 52% in extension and 157% in flexion (P≤ 0.02), with no effects in lateral bending (P≥ 0.12) Conclusions: Head posture influences the initial force generating capacity of the neck in direction-specific ways. These results will help to identify directional planes that may increase the vulnerability to higher head accelerations post- contact and guide sport-readiness training and coaching.

**Keywords/Mots clés:** paediatric concussion, neck strength, neck biomechanics

# Abstract: A038 – Sit-to-stand biomechanics in younger and older adults

**Published:** 2013-05

**Authors:** C Gray, T Guy, D Yoon, KY Lui, E Culham

**Affiliations:** School of Rehabilitation Therapy, Queen’s University, Kingston, ON

**Purpose & Objectives / Intention & objectifs:** Determine effect of age on temporal, weight bearing and center of pressure (COP) and center of mass (COM) excursion measures during sit-to-stand.

**Relevance/Pertinence:** Falls in older adults occur during dynamic tasks including rising to standing from a sitting position. Comprehensive analysis of sit-to-stand is needed to a better understand the challenges associated with this complex task.

**Materials & Methods/Matériel & méthods:** Fifteen young adults (24.3±2.1 years) and 15 older adults (76.3±5.2 years) participated. We used three AMTI force plates and the OPTOTRAK 3020 motion analysis system to obtain measures of sit-to-stand time and time and percent spent in phase one (start to seat off) and phase two (seat off to end), total and dominant limb vertical ground reaction force (GRF) at seat-off, and COP and COM excursion in each phase of STS.

**Analysis/Analyse:** Independent t-tests.

**Results/Résultats:** The percent of total time in phase one was lower in older adults (40.9±5.1% versus 45.4±4.8 in young group; p<0.05). There was no difference in vertical GRF at seat off between groups; participants in both groups placed approximately 50% of body weight on the dominant limb at seat off. Medial lateral excursion was significantly higher in older adults in both phase one and phase two of STS. There were no differences in antero-posterior excursion of the COP or COM. **Conclusions:** Greater excursion of the COP and COM in the frontal plane in both phases of STS is a novel finding and suggests that older adults have greater medial lateral instability throughout the sit-to-stand task.

**Keywords/Mots clés:** sit-to-stand, ageing, biomechanics

# Abstract: A039 – Physical Therapy for Persons with Neurological Conditions in Ontario Home Care and Long Term Care

**Published:** 2013-05

**Authors:** K Berg, J Hsueh

**Affiliations:** University of Toronto, Department of Physical Therapy, 160-500 University Avenue, Toronto, ON

**Purpose & Objectives / Intention & objectifs:** To examine patterns of utilization of physiotherapy services among patient with neurological conditions in Ontario’s home care and long term care (LTC) system.

**Relevance/Pertinence:** This research illustrated the pattern of physical therapy service use by persons with neurological disorders. It characterizes the key similarities and differences between the neurological conditions and compares current service trends to best practices guides to identify areas for improvement.

**Materials & Methods/Matériel & méthods:** Subjects included persons with Amyotrophic lateral sclerosis (ALS), traumatic brain injury (TBI) and Alzheimer’s and related dementia in Ontario’s publicly funded home care and long term care.

**Analysis/Analyse:** Quantitative longitudinal analysis was conducted using Ontario RAI-HC and RAI 2.0 assessment data between 2003 and 2010. The assessments were linked to service utilization data collected by CIHI.

**Results/Résultats:** At the time of admission into home care, 11.4% of neurological clients received physiotherapy and these clients received an average of 4.6 physiotherapy sessions during their stay. Many of the clients received only 1 physiotherapy (16.2%) visit. A substantially larger proportion of head injury clients in LTC (60.9%) received physiotherapy than in home care (32.4%). Similar results were seen in persons with ALS and Alzheimer’s and related dementia. Conclusions: Physical and occupational service use in Ontario’s home care and long term care system vary widely by neurological disorder and location. Unlike residents in long term care, home care clients are less likely to receive physiotherapy sessions despite having identified problem areas that could be addressed by rehabilitation.

**Keywords/Mots clés:**

# Abstract: A040 – Sit-to-stand transfer mechanics in healthy older adults: A comprehensive investigation of a portable lifting-seat device

**Published:** 2013-05

**Authors:** D Rutherford, S Hurley, C Hubley-Kozey

**Affiliations:** Dalhousie University, Schools of Physiotherapy and Biomedical Engineering, Halifax, NS

**Purpose & Objectives / Intention & objectifs:** To evaluate lower extremity mechanics and muscle activation associated with the sit-to-stand transfer using a portable lifting-seat device and compare these data to an unassisted transfer in young and healthy older adults.

**Relevance/Pertinence:** Rising from a chair is a demanding motor task and an essential component for independent mobility. Physiotherapists prescribe lifting-seat devices, but objective evidence for their effectiveness is lacking.

**Materials & Methods/Matériel & méthods:** Using a cross-sectional, experimental design, bilateral lower extremity and low back musculature electromyography, three-dimensional leg and trunk motion and ground reaction forces were recorded from ten young (mean age=25) and ten healthy older (mean age=69) adults during 5 trials of i) no assist and ii) assisted transfers. Trunk, hip, knee and ankle angles were derived and moments of force were calculated using inverse dynamics. All data were time normalized to represent the period of seat-off to standing.

**Analysis/Analyse:** Peak sagittal plane joint angles, moments of force and muscle activity were calculated. Analysis of variance models test for main effects and interactions (α = 0.05).

**Results/Résultats:** Trunk, hip and knee angles were significantly reduced and dorsiflexion increased with assisted transfer (p<0.05). Peak hip and ankle joint moments were reduced (p<0.05) and no change found in knee moments (p>0.05). Peak muscle activity was lower during the assisted transfer (p<0.05). Findings were similar between age groups. Conclusions: In general, variables indicative of sit-to-stand functional demand were reduced with lifting-seat device use. Data provide a framework for future recommendations on product use by physiotherapists and research pertaining to the advancement of adaptive seating.

**Keywords/Mots clés:** Sit-to-stand, Lifting seat device, Electromyography, Biomechanics, Older Adults

# Abstract: A041 – The Canadian Survey of Health, Lifestyle and Aging with Multiple Sclerosis; A Preliminary Report

**Published:** 2013-05

**Authors:** M Ploughman, JD Fisk, M Stefanelli, S Beaulieu, C Harris, S Hogan, K Knox, RA Marrie

**Affiliations:** * Eastern Health & Memorial University, St. John’s NL,; † Dalhousie University, Halifax NS,; ‡ University of Saskatchewan, Saskatoon SK, §University of Manitoba, Winnipeg MB

**Purpose & Objectives / Intention & objectifs:** Early indicators of disease severity do not predict long term outcome in multiple sclerosis (MS). We surveyed older people with MS who are a wealth of knowledge about aging with a chronic disease.

**Relevance/Pertinence:** Physiotherapists can learn from older people with MS and use their expertise to influence lifestyle modification to affect successful aging with MS.

**Materials & Methods/Matériel & méthods:** We are surveying a purposeful sample of 1000 people in Canada who are 55 years of age and older with MS symptoms more than 20 years, recruited from MS clinics and through newspaper ads and MS Society chapters. The survey is comprised of patient-reported outcomes measuring exercise, diet, alcohol use, smoking, social support, financial security, activities, mental and cognitive health, functional ability, co-morbidity, health-related quality-of-life (QoL) and disease characteristics. We present the preliminary analysis of the first 240 surveys here.

**Analysis/Analyse:** How factors relate to one another and to the primary outcomes (QoL and disability) will be analyzed using structural equation modeling however we use descriptive statistics and simple regression for this preliminary analysis (p<0.05).

**Results/Résultats:** The 184 females and 56 males (aged 64±5.8yrs) reported living with MS for 33±9 years. 52% described moderate to extreme impact of MS on daily life. Social support, resilience, financial stability and mental health are significantly associated with both higher self-reported QoL (R .34, .32, .24, .23 respectively) and less disability (R .15, .19, .20, .15 respectively). **Conclusions:** Financial stability and resilience may influence health and aging with MS by impacting the person’s ability to adapt to change, modify environments and overcome barriers.

**Keywords/Mots clés:** quality of life, aging, multiple sclerosis

# Abstract: A043 – Effects of Interventions that Aim to Increase Exercise Adherence in People with Arthritis: A Best Evidence Synthesis

**Published:** 2013-05

**Authors:** K MacPherson, AM Ezzat, J Leese, LC Li

**Affiliations:** * University of British Columbia, Vancouver, BC; † Arthritis Research Centre of Canada, Vancouver, BC; ‡ Child and Family Research Institute, Vancouver, BC

**Purpose & Objectives / Intention & objectifs:** To determine the effectiveness of interventions aiming to increase exercise adherence to prescribed exercise programs in adults with arthritis using best-evidence synthesis.

**Relevance/Pertinence:** Exercise is a central component in physiotherapy treatment of arthritis, however 30–60% of patients do not follow prescribed exercises.

**Materials & Methods/Matériel & méthods:** A literature search was conducted from 1947 to December 2010 using nine databases. Eligible studies included: participants minimum 18 years old with arthritis; intervention(s) aiming to improve adherence to prescribed exercise; a control/comparison group; and, an exercise adherence outcome.

**Analysis/Analyse:** Four assessors scanned titles and abstracts, extracted data, and assessed study quality (Pedro scale). Relative difference of exercise adherence was calculated for each study.

**Results/Résultats:** The search strategy revealed 13 articles. Interventions varied from knowledge-based programs to motivational-based strategies. Three of four studies on rheumatoid arthritis (RA) were rated high quality and found that interventions were superior to a control (relative difference (RD)=20.9%-66.7%). Four of six studies on osteoarthritis (OA) were rated high quality, with only one reporting a statistically significant difference between groups (RD=32.2%-106.3%). Two low quality studies included a mix of patients with OA and RA; one reported a statistically significant result (RD=42.1), while the other did not provide sufficient data to determine statistical significance. **Conclusions:** With three high quality studies in RA, there is strong evidence supporting the effectiveness of adherence interventions. For OA, evidence neither supports nor disproves the effectiveness of these interventions. In mixed types of arthritis, the evidence is unclear. Our results suggest that future research on exercise adherence interventions should target OA.

**Keywords/Mots clés:** Exercise adherence, arthritis, systematic review

# Abstract: A045 – Efficacy of the McKenzie System of Mechanical Diagnosis and Therapy for knee osteoarthritis: a randomized controlled trial

**Published:** 2013-05

**Authors:** R Rosedale, R Rastogi, S Willis, F Filice, B Chesworth B, S May, S Robbins

**Affiliations:** McGill University, Montreal, QC; * London Health Sciences Centre, London, ON; † Western University, London, ON; ‡ Sheffield Hallam University, Sheffield, UK

**Purpose & Objectives / Intention & objectifs:** To examine the efficacy of the McKenzie System of Mechanical Diagnosis and Therapy (MDT) treatment of knee derangements in patients with knee osteoarthritis compared to physiotherapy consistent with best practice guidelines and controls.

**Relevance/Pertinence:** Due to the high prevalence of knee osteoarthritis, it is important to identify which patients will benefit from treatment and to establish which treatments are effective at limiting disease burden.

**Materials & Methods/Matériel & méthods:** Participants (n=180) with knee osteoarthritis were recruited from a tertiary healthcare centre and were randomized to intervention or control groups. The intervention group was assessed by MDT physiotherapists. Those classified as derangements (MDT-derangement) received MDT end range exercises and those classified as non-responders (MDT-nonresponders) were given physiotherapy consistent with best practice guidelines. Pain and function were assessed at baseline, two weeks and three months using the P4 pain scale and Knee Injury and Osteoarthritis Outcome Score (KOOS).

**Analysis/Analyse:** Two-way mixed model analysis of covariance, with adjustment for baseline scores examined main effects of intervention and time.

**Results/Résultats:** MDT-derangement group had significantly lower pain (P4, KOOS) and improved function (KOOS) compared to MDT-nonresponders and controls at two weeks (p<0.01). At three months, MDT- derangement group had significantly lower pain than the other groups and significantly better function than controls (p<0.05). Conclusions: MDT treatment was effective at decreasing pain and improving function in patients with knee osteoarthritis although the effect was diminished at three months. MDT classifies patients into subgroups that are likely to respond to specific exercises and should be utilized to treat peripheral joint conditions.

**Keywords/Mots clés:** Knee, Osteoarthritis, McKenzie, randomized control trial

# Abstract: A046 – Predicting Acute Recovery of Physical Function Following Total Knee Arthroplasty

**Published:** 2013-05

**Authors:** SM Robbins, R Ravi, TL McLaughlin

**Affiliations:** * School of Physical and Occupational Therapy, McGill University, Montreal, QC; † University Hospital, London Health Sciences Centre, London, ON

**Purpose & Objectives / Intention & objectifs:** To explore predictors of physical function recovery during acute inpatient rehabilitation after total knee arthroplasty (TKA).

**Relevance/Pertinence:** Long term predictors of TKA success have been found but few studies have investigated short term recovery. Identifying patients that could have delayed recovery will assist in discharge planning and resource allocation.

**Materials & Methods/Matériel & méthods:** Participants (n=72) underwent TKA due to end-stage knee osteoarthritis and participated in an acute inpatient rehabilitation program in this observational study. Dependent variables included length of stay and physical function measures taken on post-operative day three: Timed Up and Go Test (TUG) and Western Ontario and McMaster Universities Osteoarthritis Index physical function subscale (WOMAC-function). Potential predictors measured on post-operative day one included age, sex, body mass index, pain intensity, contra-lateral knee extensor strength, comorbidities, and pain catastrophizing.

**Analysis/Analyse:** Multiple linear regression analyses explored relationships between dependent variables and potential predictors.

**Results/Résultats:** Decreased TUG performance was significantly associated with increased age and increased co-morbidities (R2=0.20, p<0.05). Increased pain intensity was significantly associated with worse WOMAC-function scores although only a small portion of variance in the WOMAC-function was explained (R2=0.08, p<0.05). Longer length of stay was significantly associated with increased age and increased co- morbidities (R2=0.17, p<0.05). Conclusions: Age and co-morbidities predicted physical performance and length of stay in inpatient rehabilitation following TKA. Pain intensity related to patient’s self-assessment of their physical function status. More resources should be allocated to older patients with increased co- morbidities to ensure successful short term recovery.

**Keywords/Mots clés:** total knee arthroplasty, recovery, predictors, physical function, rehabilitation

# Abstract: A047 – Evidence to Guide which Patient Reported Outcome Measure to Choose in Total Knee Arthroplasty Candidates

**Published:** 2013-05

**Authors:** DM Kennedy, PW Stratford, S Denis, P Dickson, J Andrion, JD Gollish

**Affiliations:** Sunnybrook Holland Orthopaedic & Arthritic Hospital, Toronto, ON; School of Rehabilitation Science, McMaster University, Hamilton & Department of Physical Therapy, University of Toronto, Toronto, ON

**Purpose & Objectives / Intention & objectifs:** To compare the cross-sectional validity of the Knee Injury and Osteoarthritis Outcome Score (KOOS), KOOS-Physical Function Short Form (KOO-PS), and Lower Extremity Functional Scale (LEFS) in total knee arthroplasty (TKA) candidates with osteoarthritis.

**Relevance/Pertinence:** Physiotherapists treating the arthroplasty population lack comparative evidence to select from the growing number of patient reported functional outcome measures.

**Materials & Methods/Matériel & méthods:** TKA candidates were assessed (n=334) using the KOOS, LEFS, 6-minute walk test (6MWT) and Timed-up-and-go (TUG). KOOS-PS scores were abstracted from the full KOOS.

**Analysis/Analyse:** We assessed cross-sectional validity by correlating self-report measures’ scores with a performance score obtained by pooling information from the 6MWT and TUG. Among measure correlations were compared using Meng’s test for dependent data. We also assessed cross-sectional validity using Receiver Operating Characteristic (ROC) curve analysis where the reference standard was whether or not patients required a walking aid for the 6MWT.

**Results/Résultats:** The sample included 209 females and 125 males, mean (SD) age of 63.3 (9.9) years. Cross-sectional correlation coefficients with the pooled performance score were: KOOS Function in daily living (ADL) 0.41, KOOS Sport 0.29, KOOS-PS 0.33, and LEFS 0.49. LEFS correlation with the pooled performance score was significantly greater (p<0.006) than the KOOS ADL, KOOS Sport, and KOOS-PS. ROC curve areas were KOOS ADL 0.70, KOOS Sport 0.68, KOOS-PS 0.68, and LEFS 0.76. Only the comparison between the LEFS and KOOS-PS was statistically significant (p=0.039). **Conclusions:** The KOOS-PS is not recommended as the measure of choice in TKA candidates. Although results were in favor of the LEFS, more research is warranted.

**Keywords/Mots clés:** Patient Reported Outcome Measures, Osteoarthritis, Total Knee Arthroplasty

# Abstract: A048 – A new paradigm to study strategies for obstacle circumvention and spatial navigation in a virtual environment post-stroke

**Published:** 2013-05

**Authors:** A Darekar, A Lamontagne, J Fung

**Affiliations:** † School of P & OT, McGill University, Montreal, QC; * Feil & Oberfeld CRIR Research Center, Jewish Rehabilitation Hospital, Laval, QC

**Purpose & Objectives / Intention & objectifs:** Sensorimotor and perceptuo-cognitive dysfunctions post- stroke often lead to difficulty in dealing with obstacles during community ambulation. The purpose of this study is to assess the different strategies utilized for obstacle avoidance using innovative virtual environments (VE) involving perceptuo-locomotor (PL) and perceptuo-motor (PM) tasks.

**Relevance/Pertinence:** This study improves our understanding of the motor and perceptual strategies used for obstacle circumvention in young adults as well as those post-stroke.

**Materials & Methods/Matériel & méthods:** This pilot study included 8 healthy young subjects (age 18 ± 0.46 years) as well as an elderly person (68 years) and one post-stroke individual (43 years, left hemiparesis). They navigated with a joystick while sitting (PM) or walking (PL) in a virtual room (7m by 5m) with a central target (7m ahead) and three movable obstacles located on a 3.5m-radius and approaching randomly from straight- ahead, or 30º left/right. The instruction was to navigate to the target while avoiding collision with the moving obstacle.

**Analysis/Analyse:** Descriptive statistics were used to calculate differences in clearance from the obstacle between participants.

**Results/Résultats:** The post-stroke subject maintained smaller clearance as compared to the older subject or the healthy young group in both the PM and PL tasks and preferred to circumvent towards the non-paretic side irrespective of the obstacle approach direction in walking. A virtual collision was experienced only in the post-stroke subject during walking with the obstacle approaching from the paretic side. Conclusions: Smaller clearances, regardless of PL or PM tasks, are indicative of a higher risk of collisions, as exhibited by the post- stroke individual.

**Keywords/Mots clés:** virtual reality, visuo-spatial perception, navigation, locomotion, rehabilitation

# Abstract: A049 – Can persons with visuospatial neglect after stroke safely avoid moving obstacles while walking?

**Published:** 2013-05

**Authors:** G Aravind, A Lamontagne

**Affiliations:** School of Physical & Occupational Therapy, McGill University, Montreal, QC; Feil and Oberfeld Research Center, Jewish Rehabilitation Hospital, Research cite of CRIR, Laval, QC

**Purpose & Objectives / Intention & objectifs:** The ability to negotiate commonly encountered moving obstacles such as people and vehicles might be impaired in persons with post-stroke visuospatial neglect(VSN), whose ability to detect and respond to objects on the contralesional side is altered.

**Relevance/Pertinence:** Recovery of safe and independent community walking is a crucial goal for rehabilitation post-stroke.

**Materials & Methods/Matériel & méthods:** Twelve participants with VSN were tested in a virtual environment consisting of a target and 3 obstacles, one of which randomly approached from head-on or 30° left/right. Participants pressed a joystick button on perception of a moving obstacle (perceptuo-motor task) and walked towards the target while avoiding a collision with the obstacle (locomotor task). Detection times (perceptuo-motor task) and minimum distances (MD) from obstacles (locomotor task) were measured.

**Analysis/Analyse:** Outcomes were compared across directions using repeated measures ANOVAs. Relations between locomotor and clinical outcomes were derived using correlation coefficients.

**Results/Résultats:** Seventy-five percent of participants collided with contralesional and/or head-on obstacles while walking. Participant maintained smaller distances from the colliding obstacles. For contralesional and head-on obstacles, colliders showed greater delay in detection, and in initiation of avoidance strategy than the non-colliders. There were no significant relationships between the locomotor and clinical outcomes. Conclusions: Persons with VSN are at the risk of colliding with dynamic obstacles approaching contralesionally or head-on. Maintenance of smaller MDs may reflect a spatial misrepresentation on the neglected side. Delays in response to the obstacles (both perceptual and locomotor) contribute to the colliding tendencies. Clinical measures of neglect may not be adequate to identify functional limitations in persons with neglect.

**Keywords/Mots clés:** Visuospatial neglect, walking, virtual reality, collisions

# Abstract: A050 – Effect of lower limb forced use strategies on weight-bearing and balance during sit-to-stand in people with stroke

**Published:** 2013-05

**Authors:** CK Gray, E Culham

**Affiliations:** School of Rehabilitation Therapy, Queen’s University, Kingston, ON

**Purpose & Objectives / Intention & objectifs:** To investigate the effect of forced use strategies on affected limb weight-bearing and peak-to-peak mediolateral (ML) and anteroposterior (AP) centre of mass (COM) displacement during sit-to-stand (STS) in people with stroke.

**Relevance/Pertinence:** Several forced use strategies have been proposed to improve STS performance and decrease fall risk in people with stroke. The effect of these strategies on weight-bearing symmetry and balance remains unclear.

**Materials & Methods/Matériel & méthods:** Fifteen people with stroke participated (66+11.15years). We used three AMTI force plates and the OPTOTRAK 3020 motion analysis system for data collection. Strategies included placement of solid (2.54, 5.08, 7.62 and 10.16 centimeters) or foam (2.72 and 6.63 kg density) blocks under the unaffected limb, and placing the unaffected limb ahead (one quarter and one half foot length) of the affected limb.

**Analysis/Analyse:** Repeated measures ANOVA

**Results/Résultats:** All strategies increased affected limb weight-bearing at seat-off compared to baseline (p<0.01); the largest increase was observed with the half foot forward position. There was a nonsignificant overall effect of ML COM displacement (p>0.1), however, pairwise comparison demonstrated an increase in ML COM displacement compared to baseline using the foam blocks (p<0.05). AP COM displacement was greater with use of the foam blocks and asymmetrical foot positions compared to baseline (p<0.05). Conclusions: Placement of the unaffected limb half a foot length ahead of the affected limb may be the most effective strategy for increasing affected limb weight-bearing. The two foam blocks increased ML and AP COM displacement suggesting that the foam block strategies challenge the balance system.

**Keywords/Mots clés:** Stroke, sit-to-stand, forced use strategies

# Abstract: A051 – Inducing lower-limb gait-like movements in persons with hemiparesis using complex localized muscle vibration

**Published:** 2013-05

**Authors:** C Duclos, C Kemlin, J-O Dyer, D Gagnon

**Purpose & Objectives / Intention & objectifs:** To determine whether the application of a vibration pattern, that produces gait-like sensory activity, can induce gait-like movements among hemiparetic participants in a relaxed standing position. Muscle vibrations induce illusions of movement, motor responses and cerebral activity similar to voluntary contraction. It could therefore be possible to induce complex gait-like illusions and movements by applying vibrations appropriately.

**Relevance/Pertinence:** This complex sensorimotor stimulation could serve to increase the duration of task-oriented locomotion training in persons with hemiparesis.

**Materials & Methods/Matériel & méthods:** Four healthy participants and seven patients with chronic hemiparesis due to stroke (Chedoke McMaster Stroke assessment (/7): leg [3-6], foot [2-4]) were included. Twelve vibrators were activated on lower limbs’ flexor and extensor muscles groups according to a gait-like pattern, organized in 1- or 2-seconds cycles. The subjects were supported by a body weight support system. Lower-limb kinematic data were recorded using a NDI Certus motion capture system.

**Analysis/Analyse:** The amplitude and period of cyclic movements induced by the vibrations were analyzed at the knees and hips with descriptive statistics.

**Results/Résultats:** Alternated movements of flexion and extension at the knees and hips were measured in response to the applied vibrations, with small amplitude (1.4±1.5° to 4.9±8.8°) and period of 1- and 2- seconds for more than 80% of cycles recorded. Conclusions: The application of a complex pattern of vibration can trigger, in hemiparetic subjects, rhythmic movements of small amplitude in the absence of voluntary command. It could provide a means of early and intensive rehabilitation training in this population.

**Keywords/Mots clés:** Vibration, Gait, Rehabilitation, Mass practice

# Abstract: A055 – Hip position enhances involuntary but not voluntary pelvic floor muscle activation in women with urinary incontinence

**Published:** 2013-05

**Authors:** C Auchincloss, L McLean

**Affiliations:** Queen’s University, School of Rehabilitation Therapy, 31 George St, Kingston, ON

**Purpose & Objectives / Intention & objectifs:** Pelvic floor muscle (PFM) activation is a key component of continence function.The purpose of this study was to determine if PFM activation was altered when women with urinary incontinence varied their hip position while performing different tasks including rest (tonic activation), maximum voluntary contractions (MVCs) of the PFMs, and maximal effort coughs (involuntary activation).

**Relevance/Pertinence:** Determining optimal training positions to facilitate PFM activation may enhance therapeutic outcomes.

**Materials & Methods/Matériel & méthods:** A repeated measures design was used. Electromyography (EMG) was recorded using differential suction electrodes located over the PFMs while participants performed the study tasks in supine and while standing, repeating them in a neutral hip posture, with the hips in internal rotation, and with hips in external rotation.

**Analysis/Analyse:** A general linear model was used to determine differences in EMG amplitude across the different hip positions (alpha=0.05).

**Results/Résultats:** Fourteen women with stress urinary incontinence (38–69 years old) participated. In supine, EMG activation amplitudes recorded during MVCs of the PFMs were consistent across hip positions (p=0.928). PFM tonic activity was significantly higher in the hip internally and externally rotated positions compared to the neutral hip position (p=0.003). During coughing EMG amplitudes were higher when the hips were internally rotated compared to neutral (p=0.04). Changes in hip rotation position when standing produced no significant differences in PFM EMG amplitudes. Conclusions: In conclusion, in supine, hip rotation appears to influence involuntary PFM activation in women with urinary incontinence. Treatment programs may include voluntary and involuntary tasks in a variety of hip postures to facilitate PFM activation.

**Keywords/Mots clés:** Pelvic floor muscles, Pelvic floor muscle training, Stress Urinary Incontinence, EMG, Hip position

# Abstract: A056 – Neutral lumbopelvic postures enhance pelvic floor muscle activation in women with urinary incontinence

**Published:** 2013-05

**Authors:** C Auchincloss, L McLean

**Affiliations:** Queen’s University, School of Rehabilitation Therapy, 31 George Street, Kingston, ON

**Purpose & Objectives / Intention & objectifs:** Effective activation of the pelvic floor muscles (PFMs) aides in maintaining continence.The aim of this study was to determine if changes in lumbopelvic posture altered PFM activation in women with urinary incontinence.

**Relevance/Pertinence:** Optimizing PFM activation may lead to enhanced outcomes of PFM training programs.

**Materials & Methods/Matériel & méthods:** Electromyography (EMG) was recorded using differential suction electrodes located over the PFMs while participants were at rest (ie. tonic activity), performed pelvic floor maximum voluntary contractions (PFVCs) and performed maximal effort coughs. Tasks were performed in lying and standing in a neutral posture, with an increased lumbar lordosis, and with a decreased lumbar lordosis.

**Analysis/Analyse:** A general linear model was used to determine differences in peak EMG amplitudes recorded in the different postures.

**Results/Résultats:** Fourteen women with stress urinary incontinence participated. In lying, tonic EMG activity was highest in the hyperlordoticposture (p=0.003), but when PFVCs were performed EMG amplitudes were lower in the hyperlordotic posture compared to the neutral and hypolordotic postures (p=0.001).EMG amplitudes did not differ across the postures during coughing (p=0.217). In standing, there was no significant difference in tonic activity across the postures (p=0.925). When standing with a hypolordotic posture, EMG activation amplitudes were lower during both voluntary contraction and coughing (p=0.05 and p=0.02, respectively) compared to the neutral or hyperlordotic postures. Conclusions: In conclusion, similar to what was previously found in continent women, voluntary and involuntary PFM activation may be optimal when performed in neutral/habitual postures. PFM training in different lumbopelvic postures may lead to enhanced training effects due to increased difficulty.

**Keywords/Mots clés:** Pelvic floor muscles, pelvic floor muscle training, stress urinary incontinence, lumbopelvic postures, EMG

# Abstract: A057 – A randomized comparison of physiotherapy and individual cognitive behavioural therapy in the treatment of provoked vestibulodynia – Pain and pelvic floor muscle outcomes

**Published:** 2013-05

**Authors:** S Thibault-Gagnon, C Goldfinger, CF Pukall, L McLean, S Chamberlain

**Affiliations:** * School of Rehabilitation Therapy; † Department of Psychology; ‡ Department of Obstetrics and Gynecology, Queen’s University, Kingston, ON

**Purpose & Objectives / Intention & objectifs:** Physiotherapy (PT) and cognitive behavioural therapy (CBT) have demonstrated some effectiveness in treating provoked vestibulodynia (PVD); however, their effectiveness has not been compared. This study aimed to compare the effectiveness of PT and individual CBT (ICBT) in reducing pain and improving pelvic floor muscle (PFM) tone and relaxation capacity, in women with PVD.

**Relevance/Pertinence:** Results will inform physiotherapists about the relative benefits of PT and ICBT in the treatment of PVD, to help optimize treatment planning.

**Materials & Methods/Matériel & méthods:** 20 women with PVD were randomized to 8 sessions of either PT or ICBT. Measures of pain, PFM tone, and PFM relaxation capacity were compared from pre- to post- treatment between the treatment groups.

**Analysis/Analyse:** Mixed between-within ANOVAs were used to test the treatment effectiveness.

**Results/Résultats:** Results indicate the following statistically significant and clinically meaningful improvements for both treatment groups, with no significant group differences: fewer non-sexual activities causing regularly experienced vulvar pain, fewer instances of sexual intercourse causing vulvar pain, reduced intercourse pain intensity and unpleasantness, and improved ability to relax the PFMs following a contraction. Women in the PT group demonstrated statistically significant and clinically meaningful reductions in PFM tone and fewer sexual activities causing regularly experienced vulvar pain, while women in the ICBT group demonstrated a clinically meaningful, but not statistically significant reduction in tone, and no meaningful change in the proportion of sexual activities causing vulvar pain. **Conclusions:** PT and ICBT appear to be equally effective at improving most outcomes; however, PT may be more effective at improving some elements of pain and PFM functioning.

**Keywords/Mots clés:** Provoked vestibulodynia, physiotherapy, cognitive behavioural therapy, pain, pelvic floor muscles

# Abstract: A058 – The effect of prospective monitoring and early physiotherapy intervention on arm morbidity following surgery for breast cancer - a pilot study

**Published:** 2013-05

**Authors:** C Singh, Vera M De, KL Campbell

**Affiliations:** * Fraser Health Authority, Surrey, BC; † Department of Physical Therapy, University of British Columbia, Vancouver, BC

**Purpose & Objectives / Intention & objectifs:** This study compared pre-operative education, prospective monitoring and early physiotherapy (intervention) versus pre-operative education alone (control) on arm morbidity after surgery for breast cancer.

**Relevance/Pertinence:** Significant arm morbidity is reported following surgery for breast cancer, yet physiotherapy is not part of usual care.

**Materials & Methods/Matériel & méthods:** A prospective quasi-experimental pre-test, post-test, non- equivalent group design compared two clinical sites, with Site A receiving the intervention (n=41) and Site B receiving the control intervention (n=31). At baseline (pre-op) and 7-months post-operative, shoulder range of motion (ROM), upper extremity strength, upper extremity circumference, pain, upper extremity function and quality of life were assessed.

**Analysis/Analyse:** To compare outcomes post-operatively at 7 months, we calculated the difference between post- and pre-operative values for continuous variables and compared these using independent sample t-tests. For categorical variables, we compared proportions post-operatively using chi-square tests.

**Results/Résultats:** The intervention group maintained shoulder ROM at 7-months compared to a decrease in the control group, particularly for shoulder flexion (0.63°±9.44 versus -6.1°±15.0, p=0.03). A lower incidence of arm morbidity and lymphedema was observed with the intervention compared to control, along with improved quality of life, however, this was not statistically significant. Baseline characteristics and surgical approaches differed between groups, which may have impacted the findings. Conclusions: Surveillance and early physiotherapy could be an effective intervention to identify and treatment arm morbidity following surgery for breast cancer. This pilot study provides the foundation for a larger more definitive trial.

**Keywords/Mots clés:** lymphedema; upper extremity; breast cancer, muscular strength and range of motion

# Abstract: A059 – Barriers to Returning to Physical Activity Following Gastrointestinal Cancer Surgery

**Published:** 2013-05

**Authors:** D Lai, Tung Sabrina

**Affiliations:** * Physiotherapy Professional Practice Leader, Mount Sinai Hospital, Toronto, and Lecturer, Department of Physical Therapy, University of Toronto, ON; † Faculty of Medicine, University of Toronto, Toronto, ON

**Purpose & Objectives / Intention & objectifs:** Patients discharged home after undergoing cancer surgery often experience difficulty regaining pre-operative physical function and resuming normal activity. The purpose of this study was to identify common functional limitations and barriers to resuming pre-operative level of physical function following gastrointestinal (GI) cancer surgery.

**Relevance/Pertinence:** Physical activity is known to improve the quality of life of cancer survivors. Physiotherapists can play a key role in helping patients optimize their function after surgery.

**Materials & Methods/Matériel & méthods:** 16 patients who underwent surgery for GI cancer participated in a telephone interview 6–8 weeks after surgery. The interviews followed a questionnaire in which participants rated their pre and post surgery level of function related to 10 activities. Participants were also asked to rate the extent to which 23 barriers in six domains (individual, physical, psychosocial, knowledge, healthcare access and medical) prevented them from returning to pre-operative activity and lifestyle.

**Analysis/Analyse:** Data were analyzed using descriptive statistics.

**Results/Résultats:** 75% of the participants experienced a decline in their functional level after surgery in at least one activity. Activities most frequently reported to pose functional challenges were household cleaning (50%), transportation (43.8%), laundry and stair climbing (31.3%). Transportation was the activity with the most marked decline in function; for those affected, pre-surgery status of complete independence changed to complete dependence post surgery. The most commonly reported barriers to resuming pre-operative physical activity were fatigue, fear of injury and depression. Conclusions: Patients commonly experience difficulty resuming physical activity following GI cancer surgery, and would benefit from physiotherapy intervention to improve function and optimize recovery.

**Keywords/Mots clés:** Physical Activity, Function, Gastrointestinal Cancer Surgery

# Abstract: A062 – Perceived benefits and barriers as predictors of improved physical activity in cancer survivors

**Published:** 2013-05

**Authors:** D Pringle, L Eng, C Brown, X Shen, O Halytskyy, M Mahler, C Niu, J Villeneuve, R Charow, C Lam, RM Shani, K Tiessen, D Howell, SMH Alibhai, W Xu, JM Jones, G Liu

**Affiliations:** Princess Margaret Hospital, Ontario Cancer Institute, University Health Network, Departments of Medicine, Biostatistics, Epidemiology, and Psychiatry, University of Toronto and Dalla Lana School of Public Health, Toronto, ON

**Purpose & Objectives / Intention & objectifs:** We investigated the roles of access to exercise information and perceived benefits of physical activity (PA) in relation to PA change after cancer diagnosis.

**Relevance/Pertinence:** PA improves physical function, overall health, and quality of life in cancer survivors, but patients often do not meet published PA guidelines.

**Materials & Methods/Matériel & méthods:** In a cross-sectional study, cancer survivors recruited from an academic cancer centre completed a self-administered questionnaire that examined exercise levels before and after diagnosis, and associated barriers/perceptions. Diagnosis and treatment information were obtained via chart review.

**Analysis/Analyse:** Regression analyses identified factors associated with increases/decreases in PA comparing pre-diagnosis and post-treatment time periods.

**Results/Résultats:** With a recruitment rate of 73%, 542 cancer survivors participated. 48% were male; median age was 56 years; all major solid/haematological cancers were represented. Fewer than 25% met weekly PA guidelines after treatment and only 27% either increased their activity or maintained levels above guidelines. Increased PA was associated with fewer perceived barriers (p=0.001) and improved health status (p<0.001). Participants who perceived increased benefits of PA on their quality of life, fatigue, and survival were more likely to increase their activity following diagnosis (p<0.001, p=0.01, and p=0.005, respectively). 20% reported receiving information regarding PA and cancer, but this was not associated with improved PA. **Conclusions:** Patient attitudes are the best predictor of maintaining/improving PA in cancer survivors. More effective health promotion interventions are needed to encourage PA and promote its benefits within this population. GL and JMJ are co-senior authors.

**Keywords/Mots clés:** physical activity, cancer, barriers, perceptions, access to information, behaviour change

# Abstract: A063 – Group Health Coaching: A Mixed Methods Evaluation of a Program in Support of Chronic Disease Self-Management

**Published:** 2013-05

**Authors:** SP Dufour, JA Richardson

**Affiliations:** School of Rehabilitation Science, Faculty of Health Science, McMaster University, Hamilton, ON

**Purpose & Objectives / Intention & objectifs:** To evaluate a program in support of chronic disease self- management (CDSM) that is founded on a ‘health coaching’ approach and includes supervised exercise component, delivered within a private physiotherapy setting.

**Relevance/Pertinence:** Programs focused on CDSM currently delivered within Ontario primary health care teams do not include an exercise component. Based on current epidemiological trends, guidance and monitoring by a provider who has expertise in both exercise prescription and underlying pathophysiology related to chronic conditions represents an important consideration.

**Materials & Methods/Matériel & méthods:** A mixed methods study, based on theory-driven program evaluation was completed. The quantitative component consisted of a before-after study. Four performance measures and four self-report measures were assessed in 18 adults (62.1 ± 11.99 years) with varying chronic diseases over an 8-week intervention period. The qualitative component was based on qualitative description and used focus groups to collect data from participants.

**Analysis/Analyse:** Summary statistics and paired t-tests were used to evaluate the quantitative data. An inductive thematic approach using data from participant focus group and reflective journals maintained by the researchers constituted the qualitative analysis.

**Results/Résultats:** Twelve participants completed the study. Significant improvements were seen in the 4- meter Walk Test (p < 0.001), Rapid Assessment of Physical Activity score (p = 0.010) and the Visual Analogue Scale-Fatigue (p = 0.011). Additionally, six themes were generated. Conclusions: This study demonstrated that a program in support of CDSM founded on a health coaching approach and delivered by a physiotherapists, may represent a beneficial adjunct service in the management of chronic conditions within primary health care.

**Keywords/Mots clés:** Health Coaching, Chronic Disease Self-Management, Physical Activity, Primary Health Care

# Abstract: A065 – The role of attention in fall avoidance - evaluation of dual task interference with postural and visual working memory tasks in young and older adults: does capacity limitation influence postural responses?

**Published:** 2013-05

**Authors:** M Woollacott

**Affiliations:** Department of Human Physiology, University of Oregon, Eugene, OR

**Purpose & Objectives / Intention & objectifs:** A critical issue of clinical practice relates to the continuum of function across age groups, specifically the frequency of falls among older adults (OA). The purpose of the study was to examine the effect of aging under dual task conditions by pairing cognitive and postural tasks and comparing performance on each between young adults (YA) and OA.

**Relevance/Pertinence:** (included above)

**Materials & Methods/Matériel & méthods:** We refined the technical aspect of the dual task paradigm by pairing a visual change detection task (that provides an estimate of visual working memory capacity (VWMC)) with varied postural tasks, including sitting, quiet stance and balance recovery from support surface perturbations.

**Analysis/Analyse:** Repeated measures one-way ANOVA was used to evaluate VWMC and postural measures for 34 YA and 39 OA. Both groups showed a significant decline in VWMC following perturbation, with the OA also showing a significant lower VWMC during the control condition compared to YA (YA: 3 (2.8±0.6) and OA:2 (1.8±0.7)).

**Results/Résultats:** Behavioral data showed a significant increase in stepping responses for OA in the dual task context, but not young adults. Kinematic data showed a significant increase in the number of trials that OA went up on toes (p<0.05) coincident with a significant increase in tibialis anterior (TA) amplitude (p<0.05) whereas YA used a typical ankle strategy. Conclusions: The results indicate the importance of developing dual task training activities that include up on toes and stepping strategies as OA were forced to shift from higher frequencies of an ankle strategy to these strategies under complex postural conditions.

**Keywords/Mots clés:** posture, balance, aging, dual task

# Abstract: A067 – Being a community physiotherapist

**Published:** 2013-05

**Authors:** DM Connelly

**Affiliations:** School of Physical Therapy, University of Western Ontario, London, ON

**Purpose & Objectives / Intention & objectifs:** To understand the experience of community-based physiotherapists providing health care in the homes of older adults.

**Relevance/Pertinence:** The demand for home and continuing care services is increasing without a consequent increase in the numbers of physiotherapists working in the community. With only a small minority of Canadian physiotherapists choosing to work in the community, the profession of physiotherapy needs to address how best to foster and support community-practicing physiotherapists as an underrepresented area of practice.

**Materials & Methods/Matériel & méthods:** Interpretive phenomenology methodology guided one-on-one interviews to explore the present and lived reality of the meanings, motives, and emotions of physiotherapists about their experiences working in the context of peoples’ homes.

**Analysis/Analyse:** Interviews were read and reread using interpretive analysis in a reflective process to identify themes with the aim of achieving sensitization to nuances in the words of the participants.

**Results/Résultats:** Emerging themes reflected the variety of roles assumed, flexibility and creativity required, and challenges and differences that were made in the care of older adults at home. Social conventions and interpersonal skills were foundational to the caring interactions. Conclusions: Community physiotherapists negotiate tensions in their practice arising from time constraints, multiple roles, and knowledge demands to achieve optimal outcomes for their clients within situations of complexity – older adults requiring health care living at home.

**Keywords/Mots clés:** community, experience, older adults

# Abstract: A087 – Ultrasound imaging to investigate the musculotendinous architecture of supraspinatus pre- and post-operative full thickness rotator cuff tears

**Published:** 2013-05

**Authors:** R Sachdeva, C Gerow, N Heynen, J Jiang, M Lebersback, B Quest, L Tasker, SY Kim

**Affiliations:** School of Physical Therapy and Health Sciences Graduate Department, University of Saskatchewan, Saskatoon, SK

**Purpose & Objectives / Intention & objectifs:** To determine the feasibility of using ultrasound to investigate the fiber bundle changes within the supraspinatus pre and post-operative full-thickness rotator cuff tears.

**Relevance/Pertinence:** Rotator cuff tears commonly involve the supraspinatus and are associated with fiber bundle changes which influence the functional properties of the muscle. Thus, understanding the fiber bundle changes associated with repair and recovery is critical to guide rehabilitative protocols.

**Materials & Methods/Matériel & méthods:** In this observational cohort study, five subjects (4M/1F) with full thickness tendon tears were included. Ultrasound protocol developed by Kim et al. (2010) was used to perform scans pre-operatively, and at 1 (post-op1), 3 (post-op2), and 6 (post-op3) months post-operatively in three positions: 0°, 60° passive, and 60° active glenohumeral abduction (except at post-op1 where active muscle contraction was contraindicated). Parameters measured were: fiber bundle length (FBL), pennation angle (PA), and muscle thickness (MT).

**Analysis/Analyse:** Architectural data collected from images was analyzed using SPSS. Non-parametric tests were used to examine the mean change of FBL, PA, and MT between pre- and post-operative assessments.

**Results/Résultats:** Preliminary results were not statistically significant (p<0.05) but strong trends were found. In the anterior region, mean FBL increased while mean PAs decreased at post-op1 in 0° and 60° passive glenohumeral abduction respectively. At post-op3, these parameters returned to their pre-op length. No trends were seen for MT. Fiber bundles could not be visualized in the posterior region. Conclusions: Ultrasound is feasible for assessing pre- and post-operative fiber bundle changes within the supraspinatus. Trends found could potentially affect force production and excursion velocity of the muscle and thus should be further investigated in a larger study.

**Keywords/Mots clés:** supraspinatus, rotator cuff tears, surgery, ultrasound, muscle architecture, rehabilitation

# Abstract: A088 – Test-retest reliability of the sport concussion assessment tool 2 (scat2) for uninjured children and young adults

**Published:** 2013-05

**Authors:** M Chan, JV Vielleuse, S Vokaty, MA Wener, I Pearson, I Gagnon

**Affiliations:** School of Physical and Occupational Therapy, McGill University, Trauma Programs, The Montreal Children’s Hospital, McGill University Health Center, Department of Pediatrics, McGill University, Montreal, QC

**Purpose & Objectives / Intention & objectifs:** To establish test-retest reliability of the Sport Concussion Assessment Tool 2 (SCAT2) in healthy elementary school children and young adults.

**Relevance/Pertinence:** The SCAT2 has been advocated as a standard for the recognition of concussions in sports for both adults and children as young as 10 years old but limited evidence is available regarding its psychometric properties particularly in the pediatric population.

**Materials & Methods/Matériel & méthods:** Twenty-two children (age 10.3 ± 0.59 years) and 73 young adults (age 22.6 ± 1.8 years). Eight student physiotherapists administered the SCAT2 on two occasions, approximately one week apart. Test-retest reliability was assessed for the total SCAT2 score and for the SCAT2 components separately.

**Analysis/Analyse:** Test-retest reliability was estimated using the Intraclass Correlation Coefficient (ICC).

**Results/Résultats:** ICC for the total SCAT2 score were fair for both groups of participants (ICC=0.367 for young adults and 0.446 for children). The ICCs were also below 0.5 for individual components of the SCAT2 in young adults. In children, individual components of the SCAT2 yielded better reliability than did the total score (Balance ICC=0.725; SAC ICC=0.523; symptom severity ICC=0.488). Conclusions: The results indicate that in view of the low levels of test-retest reliability achieved for the total SCAT2 score, professionals should be cautious when using change in score over time for clinical decisions. Recommending the use of the SCAT2 as a standard of care to diagnose a concussion or to ascertain recovery from one may be premature at this time.

**Keywords/Mots clés:** SCAT2, assessment tool, concussion, test retest reliability, intraclass correlation coefficient

# Abstract: A089 – Inter-rater and intra-rater reliability of shoulder position sense measurement tools

**Published:** 2013-05

**Authors:** AK Vafadar, J Cote, P Archambault

**Affiliations:** * School of Physical and Occupational Therapy; † Associate professor, Department of Kinesiology and Physical Education; ‡ Associate Professor, School of Physical and Occupational Therapy; § McGill University, Montreal, QC

**Purpose & Objectives / Intention & objectifs:** When position sense in the shoulder is compromised, the likelihood of injury is increased. Currently available measurement tools are both time-inefficient and costly. The purpose of this study was to estimate the inter-rater and intra-rater reliability of three measurement tools that are simple and at the same time affordable for all clinicians. These tools were: laser pointer (pointing at a vertical scale at a fixed distance), inclinometer and goniometer.

**Relevance/Pertinence:** Reliable and simple measurement tool can help the physiotherapists to regularly assess position sense and therefore to better manage musculoskeletal problems in the shoulder

**Materials & Methods/Matériel & méthods:** Twenty-five healthy subjects were asked to actively flex their dominant shoulder with eyes closed, starting with their arm along their body. They were told to stop when they reached to a pre-established target. They then had to reproduce the movement from memory, with the difference taken as error in position sense. Subjects performed 10 repetitions for each target and for each tool. Two experimenters tested each subject separately for inter-rater reliability. For the intra-rater reliability, subjects were tested again in 24 hours after the first session.

**Analysis/Analyse:** Intra-class correlation coefficient (ICC) was used to estimate the inter-rater and intra-rater reliability.

**Results/Résultats:** Laser pointer was shown to have the highest reliability (inter-rater=0.86, intra-rater=0.78) followed by inclinometer (inter-rater=0.67, intra-rater=0.70) and goniometer (inter-rater=0.5, intra- rater=0.6). Conclusions: The result of this study showed that the laser pointer technique has “excellent” reliability and the inclinometer and goniometer have “fair to good” reliability in the measurement of shoulder position sense. Laser pointer technique is very simple and can be used in any physiotherapy clinic

**Keywords/Mots clés:** Position sense, Measurement tool, Shoulder joint, Reliability, Proprioception

# Abstract: A091 – Effect of Box Taping as an adjunct to Comprehensive Exercise Program in correction of Forward Shoulder Posture – A Pilot study

**Published:** 2013-05

**Authors:** K Raja, MG Balthillaya

**Affiliations:** * Associate Professor, Department of Physiotherapy Manipal, College of Allied Health sciences, Manipal University, Manipal, Karnataka, IN; † Assistant Professor, Department of Physiotherapy, Manipal College of Allied Health sciences, Manipal University, Manipal, Karnataka, IN

**Purpose & Objectives / Intention & objectifs:** To determine additive effects of box taping on scapular alignment in asymptomatic subjects with forward shoulder posture. There is a need for evidence on the effectiveness of scapular taping as an adjunct to corrective exercise in clinical practice for upper quarter postural correction.

**Relevance/Pertinence:** Both interventions can be used as cost effective measures in preventing and treating upper quarter syndromes where forward shoulder posture is involved as an etiological factor.

**Materials & Methods/Matériel & méthods:** Thirty eight asymptomatic subjects with forward shoulder posture were randomised into intervention and control groups. Both groups received a supervised exercise program and postural advice for 15 sessions over 3 weeks. In addition, box taping was applied to the intervention group. Static and dynamic scapular alignment was determined by the Pectoralis minor length test, T3 distance, Normalised scapular abduction ratio and the Lateral scapular slide test, recorded at baseline, 7th and 15th sessions.

**Analysis/Analyse:** Differences overtime and between the treatment groups were determined using a mixed-model analysis of variance (ANOVA).

**Results/Résultats:** Twenty participants completed the study. Participants in both intervention and control groups were similar at the baseline. Both groups showed improvement in all outcome measures except for dynamic scapular alignment (p≤ 0.05) by the 15th session. There was no difference between groups on any of the outcome measures (p ≤ 0.05). Conclusions: Both interventions were well tolerated and resulted in improvements in postural indicators in asymptomatic subjects with forward shoulder posture. The study did not demonstrate any adjunctive benefit to scapular taping.

**Keywords/Mots clés:** Rounded shoulder posture, taping, scapular alignment, rehabilitation, scapular muscle strengthening

# Abstract: A092 – Effect of Postural Reconstruction, a neuromuscular physiotherapy approach, on genu varum and mild idiopathic scoliosis: a case study

**Published:** 2013-05

**Authors:** C Destieux, N Gaudreault, P Vautravers

**Affiliations:** Institut de Reconstruction Posturale, Université de Strasbourg, Strasbourg, FR; École de réadaptation, Faculté de médecine et des sciences de la santé, Université de Sherbrooke, Sherbrooke, QC et Faculté de médecine, Université de Strasbourg, Strasbourg, FR

**Purpose & Objectives / Intention & objectifs:** Genu varum and idiopathic scoliosis are postural disorders commonly observed in adolescents. This case report aims to document the effect of postural reconstruction on these postural disorders. Effective reduction of these conditions might prevent degenerative joint pathology, chronic pain and activity limitation.

**Relevance/Pertinence:** Postural Reconstruction, a neuromuscular physiotherapy approach, proposes a new paradigm for attempting to reduce postural disorders, postulating that these conditions originate from dysfunction of muscle tone regulation.

**Materials & Methods/Matériel & méthods:** Clinical case: A 16-year-old boy suffering from knee pain and presenting with bilateral asymmetrical genu varum and mild scoliosis. Intervention: The therapeutic principle of Postural Reconstruction, is to indirectly regulate muscle tone through a facilitation-inhibition mechanism using the phenomenon of motor overflow. Outcomes: Pain (mm visual analogue scale), intercondylar space (cm) and lumbar gibbosity and Cobb angles, measured at baseline (T0), 6 month (T1), 12 month (T2) and 26 month (T3).

**Analysis/Analyse:** Descriptive analysis.

**Results/Résultats:** Pain intensity at baseline was 40 mm for the left knee and 20 mm for the right knee; intercondylar space was 7 cm, Cobb angles were 18° and 13° for the right lumbar and left thoracic curve, respectively. Knee pain disappeared rapidly. At the end of the follow-up period (T3), the intercondylar space was reduced by 4 cm and the Cobb angles were reduced by 7°. The reduction in lumbar gibbosity was not clinically significant (2°). Conclusions: This non-invasive physiotherapy method is promising for reduction and long-term retention of morphological disorders. The next step would be to demonstrate the effect in a case series study design.

**Keywords/Mots clés:** Postural Reconstruction, physiotherapy, neuromuscular facilitation, genu varum, scoliosis

# Abstract: A093 – Fukuda and Babinski-Weil: A comparison of two clinical tests of vestibulospinal functions – A pilot study

**Published:** 2013-05

**Authors:** N Paquet, A Taillon-Hobson

**Affiliations:** School of Rehabilitation Sciences, University of Ottawa, Ottawa, ON

**Purpose & Objectives / Intention & objectifs:** Fukuda ‘stepping’ and Babinski-Weil (BW) ‘star walking’ are clinical tests that presumably detect underlying unilateral vestibular hypofunction (UVH). It is hypothesised that weight bearing in UVH is increased to the impaired side. Both tests demonstrate greater body rotation and larger horizontal displacement during walking with eyes closed as compared to people with normal vestibulospinal function. However, little is known about the characteristics of a normal BW performance. Therefore the objectives of this pilot study are to describe the rotations and displacements during BW in healthy individuals and to compare them with those obtained on the Fukuda.

**Relevance/Pertinence:** Balance assessment in clients with vertigo/dizziness

**Materials & Methods/Matériel & méthods:** Thirteen young healthy participants performed three trials of both tests. The Fukuda involved stepping in place with eyes closed for 50 steps. The BW involved repeating three cycles of three steps of forward and backwards walking with eyes closed while attempting to return to the starting position. Final feet positions were marked on the floor with tape. Lateral (x) and longitudinal (y) distances relative to the start line were measured using a ruler and body rotation was measured using a goniometer.

**Analysis/Analyse:** Non-parametric Mann-Whitney U tests were conducted.

**Results/Résultats:** On average, final feet positions (x and y distances) and body rotation were similar for both tests. However, BW demonstrated less variability of measures than Fukuda, especially for rotations (BW: -11°±30°, Fukuda:-38°±108°). Conclusions: BW outcomes may be less variable among subjects because it is made of only 18 steps, while Fukuda involves 50 steps.

**Keywords/Mots clés:** Vestibular rehabilitation, Vestibulospinal test, Balance assessment

# Abstract: A094 – People After Mild Stroke or Transient Ischemic Attack – An Overlooked Population

**Published:** 2013-05

**Authors:** M MacKay-Lyons, G Gubitz, N Giacomantonio, H Wightman, D Marsters, K Thompson, C Blanchard, G Eskes, S Ferrier, S Slipp, M Freeman, F Peacock

**Purpose & Objectives / Intention & objectifs:** Although non-disabling stroke (NDS) and transient ischemic attack (TIA) are regarded as relatively benign conditions, they actually can signal further vascular events or death. The purpose of this study was to describe baseline characteristics of participants in the Program of Rehabilitative Exercises and Education to avert Vascular Events after NDS and TIA (PREVENT) Trial.

**Relevance/Pertinence:** Understanding the clinical presentation of NDS/TIA is an important step to identifying appropriate secondary preventive interventions.

**Materials & Methods/Matériel & méthods:** Prior to randomization (PREVENT program versus usual care), 125 patients <1 month post-NDS/TIA underwent assessment of comorbidities, vascular risks, exercise capacity, and walking endurance.

**Analysis/Analyse:** Descriptive and correlational analyses.

**Results/Résultats:** Mean age of participants was 65+11 years, 66% male, and 66% with a smoking history. Most prevalent co-morbidity was arthritis (41%), followed by diabetes (19%), coronary artery disease (12%), and atrial fibrillation (11%). 2-year risk of second stroke or death was high in 24%, moderate in 34%, and low in 42%. Resting systolic blood pressure was 130+18 mmHg. During symptom-limited exercise testing participants attained 93+10% of predicted maximal heart rate and peak respiratory exchange ratio of 1.18+0.1 and 9% were positive for ischemia. High variability was found in peak oxygen consumption (19.6+6.1 ml/kg/min, 40% to 117% of predicted values) and 6-minute walk distance (males: 482+104 m, 86+14% of predicted; females: 410+99 m, 82+12% of predicted). **Conclusions:** A substantial proportion of this cohort post-NDS/TIA demonstrated vascular comorbidities, hypertension, ischemia on stress testing, and high prevalence of moderate/high risk of second stroke or death. Clinical correlates included reduced aerobic capacity and walking endurance, both potentially modifiable through exercise interventions.

**Keywords/Mots clés:** stroke, risk factors, secondary prevention, rehabilitation

# Abstract: A096 – Une version franco-québécoise du sport concussion Assessment Tool 2 (Scat2) : outil d’évaluation pour commotions cérébrales dans le sport 2 – Québec (Scat2-QC)

**Published:** 2013-05

**Authors:** I Pearson, J Boyd, ME Boyer-Rémillard, M Pilon-Piquette, P McKinley

**Affiliations:** École de physiothérapie et d’ergothérapie, Université McGill, Montréal, QC

**Purpose & Objectives / Intention & objectifs:** Traduire le Sport Concussion Assessment Tool 2 (SCAT2) selon la langue française québécoise et vérifier son acceptabilité pour la population franco-québécoise.

**Relevance/Pertinence:** La langue franco-québécoise comporte des caractéristiques particulières différentes d’un français international. La version française existante du SCAT2 comprend certains mots pouvant rendre sa compréhension difficile pour une population franco-québécoise.

**Materials & Methods/Matériel & méthods:** Le SCAT2 original a été traduit selon une approche modifiée de la méthode de traduction et d’adaptation d’outil tel que proposée par l’organisation mondiale de la santé. Une traduction parallèle a d’abord été réalisée. Une révision de cette traduction par un comité a ensuite permis d’obtenir une version préliminaire SCAT2-Qc. Une traduction parallèle inverse a ensuite été effectuée et comparée à la version originale. La version préliminaire a été modifiée par la suite. La version finale a ensuite été obtenue grâce aux commentaires/suggestions lors d’un essai de l’outil sur deux sujets sains et lors la comparaison du SCAT2-Qc avec la version française existante par trois réviseurs du domaine de la santé. La version finale SCAT2-Qc a finalement été testée sur 12 sujets sains (âge: 24 ans ± 2 ans) afin de s’assurer de son acceptabilité.

**Analysis/Analyse:** Discussion avec comité d’expert.

**Results/Résultats:** Les 12 sujets sains ont exécuté les directives du SCAT2-Qc sans erreur et sans avoir eu recours à des explications supplémentaires. Conclusions: Les étapes de traduction rigoureuses entreprises ont permis de créer le SCAT2-Qc pouvant maintenant être utilisé de façon valide dans le milieu sportif et scientifique québécois.

**Keywords/Mots clés:** commotions cérébrales, outil d’évaluation, SCAT2, traduction, validité de contenu

# Abstract: A097 – Community physiotherapists’ experiences in promoting chronic disease self-management with rural community-dwelling older adults

**Published:** 2013-05

**Authors:** L Graham, DM Connelly

**Affiliations:** Health and Rehabilitation Sciences, Western University, London, ON

**Purpose & Objectives / Intention & objectifs:** To understand community physiotherapists’ experiences of promoting chronic disease self-management among their elderly clients.

**Relevance/Pertinence:** Chronic disease self-management is a priority area identified by Canadian provincial governments and is a growing health care issue within the aging population demographic. Community physiotherapists have a significant role in promoting chronic disease self-management at home which could be further supported to optimize client health and provincial governments’ ‘aging at home’ strategies.

**Materials & Methods/Matériel & méthods:** A constructive phenomenology methodology guided semi- structured interviews with six community physiotherapists who had a caseload of at least 50% rural community-dwelling older adults.

**Analysis/Analyse:** Interviews were analyzed using a constant comparative method to construct themes representative of the physiotherapists’ experiences and actions in promoting chronic disease self- management.

**Results/Résultats:** Physiotherapists’ experiences were characterized by the importance of time to establish rapport with clients prior to initiating dialogue about self-management; and time (length and allowable number of visits) as a barrier to promoting chronic disease self-management. The outcome of previous client-physiotherapist interactions influenced participants’ approach to promoting chronic disease self- management. Physiotherapists incorporated collaborative efforts to educate clients about their chronic conditions, and integrated knowledge of clients’ physiotherapy goals, physical and social environments, and the involvement or availability of other health care practitioners and community services. Conclusions: Time and previous client interactions may strongly influence a community physiotherapist’s efforts to promote chronic disease self-management. Community physiotherapists adopt a holistic practice approach, integrating knowledge of multiple influences on client self-management behaviours when promoting chronic disease self-management with rural community-dwelling older adults.

**Keywords/Mots clés:** community physiotherapy, chronic disease, self-management, phenomenology, constructivism

# Abstract: A098 – Power training in patients with knee osteoarthritis: a pilot study on feasibility and efficacy

**Published:** 2013-05

**Authors:** D Pelletier, C Gingras-Hill, P Boissy

**Affiliations:** * Faculty of Physical Education and Sports, Department of Kinesiology, Université de Sherbrooke; † Faculty of Medicine and Allied Health, Department of Surgery, Université de Sherbrooke, QC; ‡ Research Centre on Aging, Health and Social Services Centre - University Institute of Geriatrics of Sherbrooke (CSSS-IUGS), QC

**Purpose & Objectives / Intention & objectifs:** To explore the feasibility and efficacy of using a power training exercise program for the quadriceps femoris (QF) in elderly women with knee osteoarthritis (OA).

**Relevance/Pertinence:** Power training with elastic bands is a feasible treatment modality in elderly women with knee OA. Improvements in participants’ muscle power, pain and self-reported functional status were demonstrated.

**Materials & Methods/Matériel & méthods:** Design: One group quasi-experimental with pre and post intervention measurements. Participants: Seventeen women (mean age 60.3 ± 6) with knee OA pain. Setting: Outpatient physiotherapy clinic. Interventions: Bilateral QF exercise program (24 sessions in 8 weeks) consisting of 3 series of 10 repetitions of flexion-extension as fast as possible with 40 % of their one-repetition maximum (1-RM).

**Analysis/Analyse:** Knee function and associated problems using the Knee injury Osteoarthritis Outcome Score (KOOS) questionnaire and the weekly mean pain score from pain diaries using visual analogue scale (VAS) were used as primary outcomes. QF strength (QFS), power (QFP) and work (QFW) measured with an isokinetic dynamometer were used as secondary outcomes.

**Results/Résultats:** Significant improvements (p<0.05) were noted on the five categories of the KOOS. Significant decrease (p<0.01) was noted in pain intensity on VAS. The QFP and QFW increased significantly on both sides (p<0.05). Exercise compliance of 99.5% for 16 participants. Conclusions: A short power training exercise program is a feasible training modality with knee OA patients. Significant functional improvements can be achieved. Further studies must be conducted to better understand the effects of the program parameters and the generalizability of the findings.

**Keywords/Mots clés:** Osteoarthritis, quadriceps muscle, muscle strength, exercise therapy, resistance training

# Abstract: A099 – The feasibility and acceptability of Neuromuscular Electrical Stimulation (NMES) in Patients with Advanced Cancer

**Published:** 2013-05

**Authors:** TY Windholz, T Swanson, BL Vanderbyl, RT Jagoe

**Affiliations:** McGill Cancer Nutrition Rehabilitation Program Clinic, Jewish General Hospital, Montreal, QC

**Purpose & Objectives / Intention & objectifs:** The McGill Cancer Nutrition-Rehabilitation Program addresses weight loss, nutritional symptoms and impaired functional status in ambulatory patients with advanced cancer. Exercise training is a central component of the intervention plan. Regular attendance for hospital- based exercise training is difficult for patients with poor performance status, or those that live far from hospital.

**Relevance/Pertinence:** The objective was to assess the feasibility and acceptability of Neuromuscular Electrical Stimulation (NMES) to facilitate improvements in physical functioning for this patient population.

**Materials & Methods/Matériel & méthods:** Patients who were unable to attend regular exercise training were recruited. All assessment, instruction, and follow-up was performed by the study physiotherapist. Participants were asked to use NMES at home to both quadriceps for 30 minutes daily for 6 weeks. Compliance was measured using patient-completed diary. Acceptability and feasibility were assessed using compliance scores and questionnaire. Physical functioning was assessed at the start and end of study using performance status (PS), repeated sit-to-stand (STS), 6-minute walk test (6MWT).

**Analysis/Analyse:** 15 participants were recruited (9(60%) male, mean(SD) age 68(9) years), 13(87%) had PS 2-3. 5(33%) patients did not complete the study (4 for medical reasons, 1 patient dropped out due to machine discomfort).

**Results/Résultats:** For patients completing treatment, mean compliance was 57%. NMES was well-received overall, with patients reporting 6 weeks’ treatment as acceptable (mean score 3.9/5) and helpful (mean score 4/5). Assessment of physical functioning showed a general improvement trend after NMES: PS (0.4, p=0.03), STS (1.2s). Conclusions: Home NMES is a feasible and acceptable exercise intervention with potential beneficial functional impact even in advanced cancer patients.

**Keywords/Mots clés:**

# Abstract: A100 – A Population Based Study to Evaluate the Impact of Hysterectomy on Work-Related Low Back Injury Rates in Healthcare Workers

**Published:** 2013-05

**Authors:** C Backman, M Koehoorn, RL Franche, E Parent

**Affiliations:** * Professor and Chair, Occupational Science and Occupational Therapy, UBC, Vancouver, BC; † Associate Professor, UBC Population and Public Health, Vancouver, BC; ‡ Adjunct Professor, UBC School of Population and Public Health, Vancouver, BC; § Assistant Professor, Physiotherapy, University of Alberta, Edmonton, AB

**Purpose & Objectives / Intention & objectifs:** Women with abdominal hysterectomy have been identified as a high risk group for low back injury in clinical practice. After caesarian section, hysterectomy is the most common surgery for Canadian women. Work related injury claim rates for the healthcare sector are rising when compared with the all-sectors rate. Low back strain accounts for 25% of claims. The goal of this study was to assess if abdominal hysterectomy was contributing to the rate of low back injury in these female workers.

**Relevance/Pertinence:** Discovery of contributing factors to low back injury rates could help to inform prevention initiatives.

**Materials & Methods/Matériel & méthods:** We conducted a retrospective cohort study measuring incidence density rates of work-related low back injury in healthcare workers with and without hysterectomy using data extracted from the Population Data BC linked databases. The initial extract included 37,057 female healthcare workers but after removal of individuals with previous back pain/injury (a significant risk factor for subsequent back injury) 10,538 remained. Of these, 83 individuals with hysterectomy went on to back injury within a five year follow up window.

**Analysis/Analyse:** Incidence Density ratios were compared in the hysterectomy and no hysterectomy groups. Due to the small number of hysterectomies with the outcome, stratification by hysterectomy type, age, or other variable(s) was not possible.

**Results/Résultats:** A non-significant incidence density rate ratio of 1.21 was noted in the exposed group.

**Conclusions:** Exclusion criteria may have been too restrictive. Further analysis of the full cohort is warranted.

**Keywords/Mots clés:** Population-based, hysterectomy, work-related low back injury, female, healthcare worker

# Abstract: A101 – Performance of subjects with a lateral ankle sprain after an early neuromuscular rehabilitation program

**Published:** 2013-05

**Authors:** M Perron, H Moffet, H Bouyer, M Bastien, LJ Hébert

**Affiliations:** Université Laval, Faculté de médecine, département de réadaptation, Québec, QC

**Purpose & Objectives / Intention & objectifs:** The aim of this quasi-experimental study is to explore the effects of two rehabilitation approaches on ankle disability and functional performance of subjects with lateral ankle sprain.

**Relevance/Pertinence:** Chronic ankle instability frequently persists after lateral ankle sprain. Although neuromuscular training improves function in peoples with chronic instability, little is known on its effectiveness compared to conventional rehabilitation in the early stages of recovery.

**Materials & Methods/Matériel & méthods:** We randomized fifteen soldiers with lateral ankle sprain to either a 6-week neuromuscular training group (8 subjects) or to conventional rehabilitation (7 subjects). Neuromuscular training included progressive one-leg balance and coordination exercises while conventional approach involved PRICE modalities, joint mobilizations and home exercises. We used an aged-matched group of 10 healthy subjects as reference group. Participants filled out the FADI questionnaire and performed the Multiple Hop Test 8 weeks after the injury.

**Analysis/Analyse:** We used the Kruskal-Wallis test and Mann-Whitney tests with Bonferroni correction for group comparisons.

**Results/Résultats:** We found no significant difference between groups with ankle sprain in the FADI and Multiple Hop test scores. All scores in the conventional group differed from those of the healthy group. However, the sport module and the total FADI scores of the neuromuscular group did not differ from those of the healthy group (p=0.09 and p=0.13) and were very close to reference values (median: neuromuscular group: sport: 92%, total FADI: 98%; reference values 100%). Conclusions: These preliminary results suggest that neuromuscular training facilitates return to sport activities after lateral ankle sprain and results in a better ankle function in daily activities.

**Keywords/Mots clés:** Ankle sprain, Exercices program, Performance

# Abstract: A102 – Effect of pulsed electromagnetic field therapy on experimental pain: a double-blind, randomized study

**Published:** 2013-05

**Authors:** K Beaulieu, P Beland, A Belletete, A Couture, M Pinard, H Corriveau, G Leonard

**Purpose & Objectives / Intention & objectifs:** To evaluate the analgesic effect of pulsed electromagnetic field (PEMF) therapy using an experimental pain paradigm.

**Relevance/Pertinence:** Clinical studies suggest that PEMF therapy can decrease pain. To date, however, it remains difficult to determine if the analgesic effect observed in patients is attributable to a direct effect of PEMF on pain or to an indirect effect of PEMF on inflammation and healing.

**Materials & Methods/Matériel & méthods:** Twenty-four healthy subjects (mean age 22 ± 2 years; 8 males) participated in the experiment. Real (3 mA, 60 Hz) or sham PEMF was administered for 10 minutes on the right forearm of every participant using a double-blind, randomized, cross-over design. Experimental pain was evoked with a 9 cm2 thermode, applied on the ventral aspect of the right forearm (two minutes stimulation at constant temperature, subject adjusted). Pain intensity and unpleasantness was evaluated using a 0–100 numerical scale before and after the application of PEMF.

**Analysis/Analyse:** For each condition (i.e., sham and real PEMF), repeated-measures analysis of variance (ANOVA) were performed to compare pain scores before and after the application of PEMF.

**Results/Résultats:** We observed no change in pain intensity and unpleasantness scores following the application of real or sham PEMF therapy (all p-values > .12). Conclusions: Although we cannot exclude the possibility that PEMF therapy can decrease pain via its effect on healing and inflammation, the present observations suggest that PEMF therapy does not directly influence pain.

**Keywords/Mots clés:** Pain, electrotherapy, electromagnetic field

# Abstract: A103 – Influence of Music on Gait Speed in Fibromyalgia

**Published:** 2013-05

**Authors:** S Prasanna, NE Mayo, MJ Simmonds

**Affiliations:** * School of Physical and Occupational Therapy, McGill University, Montreal, QC; † Centre for Interdisciplinary Research in Rehabilitation of Greater Montreal (CRIR), Montreal, QC; ‡ Department of Physical Therapy, University of Texas Health Science Center, San Antonio, TX

**Purpose & Objectives / Intention & objectifs:** Fibromyalgia is associated with widespread pain, movement slowing, fatigue and depression. Music is known to decrease pain, improve movement and mood while reducing perceived exertion during movement. Whether music can improve movement, while reducing pain and improving mood in people with fibromyalgia remains unknown. The objective of this study was to examine the effects of music on gait speed, pain, mood and perceived exertion in people with fibromyalgia.

**Relevance/Pertinence:** Music can be a potential tool for individuals with chronic pain to move faster without increasing pain levels and thus be easily transferred to the clinic.

**Materials & Methods/Matériel & méthods:** Single subject alternating treatment design was used. Eight women with fibromyalgia (mean age 50±9.1 years) walked for three minutes in each of the three music conditions - fast (140 beats/min), slow (80 beats/min) and control (no music). The test walk path included area covered by a GAITRite instrumented walkway. Outcomes included gait speed, pain, mood and perceived exertion.

**Analysis/Analyse:** Visual analysis was combined with statistical analysis using the split middle technique. Graphed data was analyzed for changes in levels, trends and variability within and across conditions.

**Results/Résultats:** Visual analysis showed that average gait speed was higher with fast music and lower with slow music. Statistical analyses showed that four subjects walked faster with fast music (p<0.05). Pain, mood and perceived exertion did not change despite increased gait speed. Conclusions: Results provide evidence supporting the use of music, especially of fast tempo, to improve gait speed and movement efficiency in people with fibromyalgia.

**Keywords/Mots clés:** Fibromyalgia, Music, Gait

# Abstract: A104 – The Pathoanatomical Response to Flexion Exercises for the Discs, Spinal Canal and Lateral Foramen Is Different between Responders and Non-responders to a 4-week Flexion Exercise Program for Low Back Pain

**Published:** 2013-05

**Authors:** EC Parent, S Dhillon, J Fritz, A Long

**Affiliations:** * University of Alberta Department of Physical Therapy, Edmonton, AB; † University of Alberta, Department of Radiology, Edmonton, AB; ‡ University of Utah Division of Physical Therapy, Salt Lake City (Utah), US; § Bonavista Physical Therapy, Calgary, AB

**Purpose & Objectives / Intention & objectifs:** To compare the pathoanatomical changes on MRI resulting from flexion exercises between responders and non-responders to a flexion exercise program for low back pain (LBP).

**Relevance/Pertinence:** McKenzie flexion exercises provide functional benefits for some patients with LBP. Understanding of how exercises affect lumbar pathoanatomy may help identify patients benefiting from flexion and improve exercises.

**Materials & Methods/Matériel & méthods:** Forty-nine volunteers with LBP, aged 18–65 years and with >20% Oswestry disability participated a 4-week flexion exercise program. At baseline, we obtained lumbar MRI’s (T12-S1) immediately before and after performing repeated flexion exercises. We digitized lumbar canal diameter on mid-sagittal images and disc perimeters, lateral foramen diameters, and canal area on mid-disc axial images with high reliability. Patients experiencing >50% improvement in Disability at 4 weeks were deemed responders.

**Analysis/Analyse:** We normalized MRI measurements by vertebra dimensions to account for subject size. We used independent t-tests to compare MRI changes between responders and non-responders.

**Results/Résultats:** Disability improved by 27±32 with 12 deemed responders versus 37 not. At L5S1, responders had a significantly larger increase in disc area and larger decreases in the area and perimeter of the spinal canal than non-responders on the baseline MRIs. At L1L2, responders experienced more reduction in lateral foramen diameter on the painful side and an increase in anterior disc height rather than a decrease in non-responders. No significant differences were observed at L2L3, L3L4, or L4L5. Conclusions: Patients with LBP responding to a 4-week flexion exercise program show different pathoanatomical response to flexion exercises measured by MRI at baseline than non-responders.

**Keywords/Mots clés:** Low back pain, Exercises, Magnetic resonance imaging, Central foramen, Lateral foramen, Disc

# Abstract: A107 – Perception of optic flow and regulation of walking speed in young-old adults in comparison to young adults

**Published:** 2013-05

**Purpose & Objectives / Intention & objectifs:** This study aims to identify whether there is a relationship between speed discrimination and modulation of walking speed and to contrast discrimination thresholds and gait speed adaptations in young versus young-old adults.

**Relevance/Pertinence:** Walking speed is often decreased in older age and can be influenced and improved by the perception of optic flow (OF).

**Materials & Methods/Matériel & méthods:** Young-old adults (N=12, mean age = 68.83 ± 4.39 years) and young adults (N=12, mean age = 23 ± 2.41 years) participated in two experiments. Experiment 1: subjects were shown two scenes depicting forward motion along a virtual corridor in a helmet-mounted display (HMD). Subjects were instructed to choose which of the two appeared to move faster. Experiment 2: subjects walked on a self-paced treadmill while wearing the HMD. Participants were exposed to OFs at speeds ranging from 0.25 to 2 times their comfortable speed. Each test trial was paired with a control trial performed at comfortable speed with matching OF. During the test trial, subjects were instructed to walk the distance within the same time as during the preceding control trial.

**Analysis/Analyse:** Speed discrimination thresholds were determined by a two alternative forced choice paradigm (2AFC).Speed modulation was determined by the ratio of gait speed changes in the test trial over the control trial.

**Results/Résultats:** Thresholds were greater in young-old adults.The slope ratio was greater in young adults. Conclusions: Age affects the modulation of gait speed and the perception of OF speed. A significant relationship between discrimination and modulation of walking speed was not observed.

**Keywords/Mots clés:**

# Abstract: A108 – Processing Strategies Parents Use to Synthesize Health Information Related to their Child

**Published:** 2013-05

**Purpose & Objectives / Intention & objectifs:** To explore the evolution of parent learning and the strategies they used to process health information related to their child and their condition

**Relevance/Pertinence:** Relevant information is vital for parents of children with developmental condition in understanding and adjusting to the uncertainty of the disability, in supporting their child and helping them gain control. In spite of the widespread dissemination of information materials, parent dissatisfaction is frequently reported. However, limited evidence exists on how parents process the information once provided.

**Materials & Methods/Matériel & méthods:** The study utilized phenomenological design. A purposive sample of eleven parents were interviewed using in-depth face-to-face interviews using an interview guide.

**Analysis/Analyse:** A thematic analysis process was employed as the data analysis method. A codebook was created influenced by adult learning theoretical frameworks.

**Results/Résultats:** A thematic map was created based on four inter-related theme, which emerged from the data and included Motivation, Understanding, Doing and Belonging. Three of these themes, Understanding, Doing and Belonging highly correlated with The Taxonomy of Learning Objectives Domains, Cognitive, Affective and Psycho-Motor. **Conclusions:** With the essential role information fulfills in long-term management of children with developmental disabilities, and based ongoing parental dissatisfaction with information provision regardless of the amount being provided, there is a need to develop parental education across learning domains and to assist with learning challenges Parent learning progress should be monitoring as a key outcome of rehabilitation interventions.

**Keywords/Mots clés:** Parent Education, Children with Disabilities, Information Processing

# Abstract: A109 – Effects of exercise on symptoms, cognitive and motor performance tasks using the sport concussion assessment tool (scat2) in healthy young adults and children

**Published:** 2013-05

**Authors:** N Boutros, MC Norcia, J Sammouda, CL Tran, I Pearson, I Gagnon

**Affiliations:** * School of Physical and Occupational Therapy, McGill University, QC; † Trauma Programs, The Montreal Children’s Hospital, McGill University Health Center, QC; ‡ Department of Pediatrics, McGill University, Montreal, QC

**Purpose & Objectives / Intention & objectifs:** To determine the effect of high-intensity exercise on SCAT2 score and its components in healthy young adults and children.

**Relevance/Pertinence:** As a sideline concussion assessment tool, the SCAT2 is often administered by physiotherapists after high-intensity exercise. To confidently use the SCAT2 to recognize the signs and symptoms of a concussion, it is important to understand how exercise alone impacts performance on the test.

**Materials & Methods/Matériel & méthods:** 76 healthy young adults (aged 22.5 ± 1.9 years) and 13 children (aged 10.7 ± 0.4 years) participated in the study. The SCAT2 was used as a primary outcome measure to evaluate the participants at rest (T1), 2 minutes (T2), and 25 minutes (T3) post-exercise. The exercise consisted of the Léger Test.

**Analysis/Analyse:** Two-way repeated measures ANOVA was used to determine the impact of exercise on SCAT2 score and its components over time.

**Results/Résultats:** Exercise significantly lowered the total SCAT2 score for adults and children immediately post-exertion (T2) (p=0.003). Scores returned to baseline level after 25 minutes of rest (T3). Balance and self- reported symptoms were the components of the SCAT2 most affected by exercise, while cognition remained stable over testing times. Children’s scores were significantly lower at baseline, suggesting the need for age- related norms. Conclusions: High intensity exercise has an impact on the domains assessed by the SCAT2. In order to eliminate the effects of exercise when testing individuals on the field post-injury, professionals should allow for proper rest before using the SCAT2 to diagnose a concussion.

**Keywords/Mots clés:**

# Abstract: A110 – Inter-rater reliability of functional screening tests for rowers

**Published:** 2013-05

**Authors:** J Schearer, J McGivery, Huizen J Van, B Chesworth, S DiCiacca

**Affiliations:** * Physiotherapist, Stittsville Mainstreet Physiotherapy, Stittsville, ON, Canada; Murray M, Physiotherapist, Stadium PhysiOsteo, Montreal, QC; † Owner/Physiotherapist, Advanced Health & Physio, Moncton, NB; ‡ Physiotherapist, OrionHealth Rehabilitation & Assessment Centers Surrey, BC; § Associate Professor, School of Physical Therapy, University of Western Ontario, London, ON; ¶ Physiotherapist, Fowler Kennedy Sport Medicine Clinic, London, ON and IST lead, Rowing Canada Aviron, London Training Centre, London, ON

**Purpose & Objectives / Intention & objectifs:** To assess the inter-rater reliability of screening tests applied to rowers.

**Relevance/Pertinence:** As the sport of rowing has grown in popularity, so has the incidence of rowing- related injury. A pre-season screening tool will help physiotherapists identify injury risk and assist in injury prevention.

**Materials & Methods/Matériel & méthods:** Rowers (n=66) were recruited from the Ottawa Rowing Club and the Western University Rowing Team. Participants performed 12 screening tests designed to assess musculoskeletal strength and flexibility relevant to rowing. Physiotherapists (n=2) independently assessed test performance. Test order was random and results blinded.

**Analysis/Analyse:** The unweighted kappa statistic (k) was calculated for each test.. k=0.60 was chosen as the threshold for acceptable agreement.

**Results/Résultats:** Tests with the highest level of agreement included the bilateral squat, long sit flexibility, supine arm hang, and ankle dorsiflexion (k=0.64-0.84). Tests with the lowest level of agreement included the modified Thomas test-method A: with the hip positioned in extension (k=0.20) and the ergometer test (k=0.36). The following five tests demonstrated inconsistent reliability findings between the left and right sides: the prone and supine active straight leg raise, unilateral bridge, unilateral squat and the prone plank. Conclusions: Tests displaying high agreement should be kept in the screen. Those demonstrating poor agreement should be omitted or modified. Tests that demonstrated lateral disparity in agreement findings, but still met the threshold for acceptable agreement on one side, should remain in the screen. Further evaluation of the rowing screen’s ability to predict injury is necessary.

**Keywords/Mots clés:** evaluation, injury prevention, movement, rowing, reliability

# Abstract: A111 – Fitness in military personnel: Are they training to meet test requirements only?

**Published:** 2013-05

**Authors:** S Roopchand–Martin, G Nelson, S Smith

**Affiliations:** School of Physical Therapy, University of the West Indies, Mona Campus, Kingston, JM

**Purpose & Objectives / Intention & objectifs:** The Army Physical Fitness Test (APFT) battery consists of push- ups, sit-ups and a 2-mile run test, which covers only the cardiorespiratory and muscular endurance domains of fitness. This basic test is still used by many military facilities today, even though many of their activities also require flexibility, agility and power. It is suspected that military personnel engage in training specific to the APFT requirements, rather than comprehensive training. Our study sought to explore this by assessing other fitness parameters in military personnel whose annual test requirements is the basic APFT.

**Relevance/Pertinence:** Physical Therapists are well-positioned to advise military facilities on fitness testing practices when required.

**Materials & Methods/Matériel & méthods:** All participants (n = 620) were required to complete the APFT battery, the sit-and-reach flexibility test, the Illinois agility test and the standing broad jump test. The order of testing was randomized to control for carry-over effects.

**Analysis/Analyse:** Raw data for each test were entered into the relevant fitness calculators on www.exrx.net and www.topendsports.com websites. Performance was ranked as ‘poor’, ‘fair’, ‘average’, ‘good’ or ‘excellent’. Descriptive statistics was used to interpret the data.

**Results/Résultats:** For the APFT battery, more than 95% of participants had fitness scores of ‘average’ and above. Participants scored below ‘average’ in tests of flexibility (100%), agility (41.5%) and lower-limb power (87.1%). Conclusions: The results suggest that participants in the study were primarily engaging in training that was specific to the APFT requirements. Facilities using the APFT only should consider expanding this to include other parameters of fitness.

**Keywords/Mots clés:** Army Physical Fitness Test, training, fitness

# Abstract: A113 – Superficial and deep abdominal muscle activity in adults with and without cystic fibrosis

**Published:** 2013-05

**Authors:** A Taiilon-Hobson, S Aaron, L McLean, M Bilodeau

**Affiliations:** * Research Associate, Elisabeth Bruyere Research Institute, Ottawa, ON; Associate Professor, University of Ottawa, Rehabilitation Sciences, Ottawa, ON; † Chief, Respiratory Medecine, Ottawa Hospital, Ottawa, ON; Senior Scientist, Ottawa Health Research Institute, Ottawa, ON; ‡ Professor, Rehabilitation Sciences, Queen’s University, Kingston, ON; § Director, Rehabilitation Sciences, University of Ottawa, Ottawa, ON; Director, Ageing and Movement, Elisabeth Bruyere Research Institute, Ottawa, ON

**Purpose & Objectives / Intention & objectifs:** The purpose of this study was to investigate the recruitment patterns of the deep and superficial abdominal muscles between a group of individuals with stable cystic fibrosis (CF) and a control group when performing a ten-second abdominal hollowing (AH) task. A second objective was to compare the between-muscle activation patterns. The chronic coughing required in (CF) may be considered a repetitive muscular activity and potentially contribute to recruitment imbalances of the abdominal mucles.

**Relevance/Pertinence:** Abdominal muscle imbalances are associated with repetitive muscle activity, mechanical low back pain, as well as urinary stress incontinence. These patterns are correctable in physiotherapy.

**Materials & Methods/Matériel & méthods:** Twenty-eight participants (14 with CF and 14 controls) performed a one-minute sustained AH exercise in supine at a target pressure of 5 mmHg on a pressure biofeedback unit (PBU).

**Analysis/Analyse:** Surface electromyography (EMG) of the abdominal muscles was recorded and calculated as a percentage of a maximum contraction (%max) using Spike version 5.06.

**Results/Résultats:** A three-way ANOVA showed a muscle x time interaction but no between group differences. A significant decrease in the IO/TrA muscle activity and a significant increase in the RA Up muscle activity during the 60 seconds of sustained muscle activity were found. Conclusions: Adults with stable CF who have a chronic cough recruit their superficial and deep abdominal muscles during a sustained AH task in a similar fashion to a control group. Therefore chronic coughing does not seem to contribute to imbalances of abdominal muscle activation in this patient population.

**Keywords/Mots clés:** abdominal mucles; electromyography; abdominal hollowing exercise; core stabilisation; PBU

# Abstract: A114 – Chest wall volumes and respiratory muscle activity in patients with Chronic Obstructive Pulmonary Disease during loaded breathing

**Published:** 2013-05

**Authors:** MA Coutinho, KS Moraes, SM Lage, DSR Vieira, VF Parreira, RR Britto

**Affiliations:** Departamento de Fisioterapia. Universidade Federal de Minas Gerais, Minas Gerais, BR

**Purpose & Objectives / Intention & objectifs:** The purpose was to evaluate the chest wall volumes, breathing pattern and the activity of sternocleidomastoid and abdominal muscles during inspiratory loaded breathing in patients with Chronic Obstructive Pulmonary Disease.

**Relevance/Pertinence:** Breathing pattern is abnormal in patients with Chronic Obstructive Pulmonary Disease. The inspiratory muscle training is used to improve the functional capacity, however it is necessary to identify if this technique do not compromise once more the breathing pattern.

**Materials & Methods/Matériel & méthods:** We evaluated the chest wall volumes and breathing pattern by optoelectronic plethysmography and muscle activities by surface electromyography at rest and during inspiratory loaded breathing (30% of maximal inspiratory pressure) of 13 male patients with COPD.

**Analysis/Analyse:** The mean rest values were compared to those during inspiratory load breathing by Student’s t-test or Wilcoxon’s test (p<0.05).

**Results/Résultats:** At rest and during inspiratory loaded breathing, the main responsible for tidal volume was the abdominal compartment. Inspiratory loaded breathing increased the volumes of chest wall, rib cage and abdomen. It also increased (p<0.05) the rib cage end-expiratory volume, chest wall end-inspiratory volume and rib cage end-inspiratory volume as well as the minute ventilation. There was a significant increase in sternocleidomastoid activity during inspiratory loaded breathing. It was found correlations between sternocleidomastoid´s activity and volume of chest wall (r=0.558; p=0.005) and volume of abdomen (r=0.425; p=0.038). Conclusions: Therefore, the inspiratory load breathing in patients with COPD are characterized by an increased mobility in the abdomen and to a higher activity of sternocleidomastoid activity without worsening breathing pattern.

**Keywords/Mots clés:** Chest wall, breathing exercises, chronic obstructive pulmonary disease, electromyography, respiratory muscle training

# Abstract: A115 – Hell Rise Test in the assessment of individuals with peripheral arterial obstructive disease

**Published:** 2013-05

**Authors:** DP Monteiro, ACR Lages, ML Basilio, COM Pires, MLV Carvalho, RJ Procopio, RR Britto

**Affiliations:** Departamento de Fisioterapia, Universidade Federal de Minas Gerais, Minas Gerais, BR

**Purpose & Objectives / Intention & objectifs:** The heel rise test is a clinical instrument proposed to assess the performance of the triceps surae muscle. This study evaluated if these test is sensitive in differentiating the functional capacities of individuals with peripheral arterial obstructive disease (PAOD).

**Relevance/Pertinence:** To plan more individual physiotherapy for individuals with PAOD it is necessary to identify the functional performance.

**Materials & Methods/Matériel & méthods:** Individuals with PAOD were assessed by the heel rise test, the Walking Impairment Questionnaire (WIQ) and the Shuttle Walk Test (SWT) to analyze: number of plantar flexions performed in the heel rise test, time (seconds) and velocity (plantar flexions per second) to perform the plantar flexions up to the point of volunteer fatigue; maximum distance walked in the SWT and scores obtained in each WIQ domain.

**Analysis/Analyse:** Based on the maximum distance walked in the SWT (cutoff = 380m) the individuals were separated in two groups. The comparison were conducted by Student t test or Mann Whitney U (p<.05).

**Results/Résultats:** Twenty-five individuals (14 male) with mean age of 63.36±9.83 years were included. The number of plantar flexions and time to perform the heel rise test were sensitive to differentiate individuals with distinct functional capacities (p=0.003 and 0.009, respectively). The number of plantar flexions was also sensitive to differentiate individuals of extreme classes on the WIQ domain (p=0.008). **Conclusions:** The heel rise test is an easy, cheap and sensitive tool to be used in clinical practice for the assessment of individuals with PAOD with distinct functional capacities.

**Keywords/Mots clés:** Peripheral arterial disease, Heel Rise Test, Walking Impairment Questionnaire, Functional Capacity, Shuttle Walk Test

# Abstract: P001 – Physiotherapy research in private practice as a means to promote community awareness. A concussion model

**Published:** 2013-05

**Authors:** S Shatil

**Affiliations:** Neuphysio Rehabilitation, London, ON; * Associate Professor, Department of Physiotherapy Manipal, College of Allied Health sciences, Manipal University, Manipal, Karnataka, IN; † Assistant Professor, Department of Physiotherapy, Manipal College of Allied Health sciences, Manipal University, Manipal, Karnataka, IN

**Overview & Objectives/Résumé & objectifs:** Using the innovative HeadSmart program as a model, this proposal session will demonstrate how clinical physiotherapy research can be applied to the private practice setting. A framework of how to effectively implement such research into practice with a vision of professional and business growth will be presented.

Upon completion of this proposal session, participants will:

1. Be able to discuss current topics in concussion management, including baseline testing, sideline management, rehabilitation and return to play as they relate to private practice.
2. Explore how answering relevant private practice clinical physiotherapy questions through research can reach populations at a grass roots level, acknowledge the barriers that private practitioners face while implementing research projects, and problem solve through such barriers.
3. Brainstorm their own research ideas and create a plan to implement them into the private practice setting as a means to increase awareness, raise the presence of physiotherapy in the community, and grow physiotherapy business.

**Relevance/Pertinence:** Physiotherapists in private practice are uniquely positioned to engage communities in change through innovative programs. As Physiotherapists, we ask clinical questions everyday, some of which have evidence to support answers, and others which rely on our individual clinical judgment. Physiotherapists regularly educate patients, other health care practitioners, and the community at large. We have knowledge of research methods and exposure to clinical research as a result of our Graduate Level of training. Typically, private practice physiotherapy maintains a focus on quality assessment and treatment of injury and disease in a profit generating business model. It is important for private practitioners to give back to both our profession and to our communities at large. Clinical research can be creatively applied in the private practice setting as a means to increase our growing body of evidence and to reach larger populations who may not otherwise access Physiotherapy, driving business as well as community awareness.

**Target Population/Population cible:** This session will be of interest to entrepreneurial physiotherapists including clinicians, managers, leaders and practice owners who wish to involve research in their practice.

**Supporting Evidence/Résumé des faits:** Physiotherapists in private practice have limited resources available to support clinical research endeavors. It is difficult to access funding through typical siurces as an independent practitioner. Even so, the Physiotherapy profession, is encouraged and developed to partake in research endeavors. Athough, the majority of published evidence in Physiotherapy comes from academic or hospital research, private physiotherapists are becoming increasingly interested in creating research based roles within their practice. The HeadStart Program is an innovative private practice research program that has led to strategic alliances, securing of funding for concsussion research, and community awareness events. The program grew out of one physiotherapists desire to create change within a community through education and awareness of current conucssion evidence, defining gaps in knowledge and clearly asking relevant questions to advance what is already accepted in concusison management.

**Session Format/formule de la session:** This session will be an interactive learning format with the opportunity to brainstorm in small groups throughout, resulting in the creation of a plan for implementing unique research ideas into private clinical practice.

**Conclusions & implications:** As demonstrated through the example of the HeadSmart program, Physiotherapists in private practice are ideally positioned to ask relevant clinical questions, coordinate research endeavors and form strategic alliances within communities that result in positive change for professionals and community members alike.

**Keywords/Mots clés:** Physiotherapy, Business, Research, Concussion

# Abstract: P039 – Physical Therapy for Persons with Neurological Conditions in Ontario Home Care and Long Term Care

**Published:** 2013-05

**Authors:** K Berg, J Hsueh

**Affiliations:** University of Toronto, Department of Physical Therapy, 160-500 University Avenue, Toronto, ON

**Purpose & Objectives / Intention & objectifs:** To examine patterns of utilization of physiotherapy services among patient with neurological conditions in Ontario’s home care and long term care (LTC) system.

**Relevance/Pertinence:** This research illustrated the pattern of physical therapy service use by persons with neurological disorders. It characterizes the key similarities and differences between the neurological conditions and compares current service trends to best practices guides to identify areas for improvement.

**Materials & Methods/Matériel & méthods:** Subjects included persons with Amyotrophic lateral sclerosis (ALS), traumatic brain injury (TBI) and Alzheimer’s and related dementia in Ontario’s publicly funded home care and long term care.

**Analysis/Analyse:** Quantitative longitudinal analysis was conducted using Ontario RAI-HC and RAI 2.0 assessment data between 2003 and 2010. The assessments were linked to service utilization data collected by CIHI.

**Results/Résultats:** At the time of admission into home care, 11.4% of neurological clients received physiotherapy and these clients received an average of 4.6 physiotherapy sessions during their stay. Many of the clients received only 1 physiotherapy (16.2%) visit. A substantially larger proportion of head injury clients in LTC (60.9%) received physiotherapy than in home care (32.4%). Similar results were seen in persons with ALS and Alzheimer’s and related dementia. Conclusions: Physical and occupational service use in Ontario’s home care and long term care system vary widely by neurological disorder and location. Unlike residents in long term care, home care clients are less likely to receive physiotherapy sessions despite having identified problem areas that could be addressed by rehabilitation.

**Keywords/Mots clés:**

# Abstract: P011 – Sport Related Concussion

**Published:** 2013-05

**Authors:** K Schneider, C Emery

**Affiliations:** * Sport Injury Prevention Research Centre, University of Calgary, Calgary, AB; † Associate Professor, Faculty of Kinesiology, University of Calgary Associate Professor, Community Health Sciences, Faculty of Medicine, University of Calgary, AB; Associate Professor, Paediatrics, Faculty of Medicine, University of Calgary, AB; Adjunct Associate Professor, Department of Physical Therapy, Faculty of Rehabilitation Medicine, University of Alberta, AB; Adjunct Associate Professor, Department of Physical Therapy, Faculty of Medicine, University of British Columbia, BC

**Overview & Objectives/Résumé & objectifs:** Learning Objectives:

1. Examine the epidemiology of concussion, including the incidence, risk factors and public health burden of concussion in the population.
2. Identify mechanism of injury, pathophysiology, gender and age differences and currently available assessment techniques.
3. Identify evidence-based treatment and return to sport guidelines for sport related concussion.

Overview: This session will be an interactive session that includes a review of the most current evidence surrounding sport related concussion in addition to practical sessions to practice the assessment and treatment techniques currently available.

**Relevance/Pertinence:** Sport related concussion is a commonly occurring injury in today’s world. The physiotherapist will often encounter individuals who have suffered a sport related concussion. Recent research suggests that physiotherapy treatment may be of benefit in this population. An understanding of signs and symptoms of concussion, evidence based assessment and treatment techniques, prognosis and prevention strategies are of utmost importance to the practicing clinician.

**Target Population/Population cible:** Clinicians working with a clientelle that may be susceptible to a sport related concussion. This session is appropriate for practitioners of all levels of experience and practice.

**Supporting Evidence/Résumé des faits:** Unintentional injuries result in more potential life years lost before age 70 than any other single health problem in Canada. In fact, sport and recreation is the leading cause of injury in youth (Emery et al, 2006). Cross-sectional survey data in Alberta estimates the rate of adolescent sport injury requiring medical attention to be 40 injuries/ 100 adolescents/ year. Concussions account for >10% of this total burden and >20% of the injury burden in some sports such as youth ice hockey. While physical activity prevents all-cause morbidity and mortality associated with a sedentary lifestyle (Blair et al, 1995, Blair et al, 1989, Helmrich et al, 1994), concussions can become a barrier to active living. Reduction of sport and recreation-related concussion would improve quality of life through the maintenance and promotion of active living. This is a critical issue in health care and in the promotion of health and wellness in our communities. There is a rapidly growing societal need to address sport & recreation injury and specifically concussion and its future health impact in young age groups during the time they are most active (and when the benefit of injury prevention strategies may be the greatest).

This session will include an evidence-based perspective examining the burden, risk and prevention of sport- related concussion across the spectrum of recreational participant through elite athlete. There will be an emphasis on the youth population participating in sport and recreation. An interdisciplinary approach to risk factor identification and concussion prevention in sport will be emphasized. Although contact sports like football and hockey expose participants to a higher risk of concussion, they can occur in any sport or recreational activity, even with the use of protective headgear and safety equipment. This presentation will provide original data and evidence from the literature related to risk factor identification and prevention strategies for sport-related concussion.

Sport related concussion is believed to be a functional rather than structural injury and may be caused by a direct blow to the head or by force transmission to the head (McCrory et al, 2009). A variety of symptoms and signs may occur following a sport related concussion. The Sport Concussion Assessment Tool 2 is a commonly used clinical tool in individuals who have suffered a sport related concussion (McCrory et al, 2009). While all concussions are individual and should be managed individually, an understanding of how to administer this tool and other evidence based assessment measures will assist the physiotherapist in initial assessment and ongoing management of this common injury.

The majority of individuals recover in the first 10 days following a sport related concussion (Benson et al, 2011). However, up to 30% may have symptoms of longer duration resulting in long term symptoms, decreased activity levels, decreased ability to perform activities of daily living and an altered ability to perform occupational and/or school activities. This also increases the risk for secondary health issues as well as burden on the public health care system.

Current treatment guidelines following a sport related concussion are rest, both physical and cognitive, followed by gradual exertion (McCrory et al, 2009). Some of the most common symptoms following a sport related concussion are headaches, dizziness and neck pain, all of which may be emanating from the cervical spine (Schneider et al, 2011). The vestibular system may also contribute to symptoms of dizziness and may be amenable to physiotherapy treatment (Alsalaheen et al, 2010). Recent research suggests that physiotherapy treatment for the cervical and vestibular systems results in a decrease in time to medical clearance to return to sport (Schneider et al, 2009, 2012). Thus, the physiotherapist is in a unique position to potentially alter the course of recovery in athletes following a concussion. Evaluation of the cervical and vestibular systems as potential sources of dysfunction will allow a greater understanding as to the source of persistent symptoms and if physiotherapy treatment is indicated. Results of a cohort study evaluating baseline values of balance, cervical, dynamic visual acuity and vestibular clinical measures and their subsequent affect on concussion risk will be reviewed. The evidence surrounding other treatment techniques for this injury will also be reviewed.

**Session Format/formule de la session:**

1. Introduction: 5 minutes
2. Epidemiology (including risk factors and prevention strategies): 15 minutes
3. Assessment and treatment techniques for the physiotherapist (including practical time): 30 minutes
4. Review and interactive discussion with the audience: 10 minutes

**Conclusions & implications:** This evidence-based approach to risk factor identification and prevention of sport and recreation-related concussion should be a key consideration for all physiotherapists in clinical practice who work with children or adults who are sport participants or elite athletes. Concussion is a commonly occurring injury today and the current treatment is rest followed by graded exertion. Some of the persistent symptoms that occur may by originating from systems that have been shown to respond to physiotherapy treatment. Thus, the cervical spine and vestibular systems should be evaluated as potential sources of symptoms following a concussion and treated appropriately. The physiotherapist is in a unique role to assess and treat individuals who have suffered a sport related concussion.

**Keywords/Mots clés:** Sport-related concussion, physiotherapy, epidemiology, assessment, treatment

# Abstract: P018 – Split-belt Treadmill Walking: A Novel Therapy for Asymmetric Gait

**Published:** 2013-05

**Authors:** KE Musselman, JF Yang, AJ Bastian

**Affiliations:** * Dept. of Neuroscience, Johns Hopkins School of Medicine and Kennedy Krieger Institute, Baltimore, MD, US; † Dept. of Physical Therapy, University of Alberta, Edmonton, AB

**Overview & Objectives/Résumé & objectifs:** Motor learning can be facilitated if errors are augmented during motor training. In other words, increasing errors may teach the learner how to correct the errors. It can be challenging, however, to amplify errors during a motor task like walking. We use a split-belt treadmill to induce errors during walking. A split-belt treadmill has a separate belt for each leg. When the belts move at different speeds, spatiotemporal errors in the gait pattern result. These errors drive the nervous system to learn a new gait pattern. Because of its ability to augment motor errors and modify gait patterns, the split-belt treadmill is an emerging approach to the study and correction of asymmetric gaits. In this session we will discuss the research findings and clinical applications of the split-belt treadmill paradigm for both adult and pediatric populations. At the end of this interactive session, participants will be able to:

1. Identify the patients who are most likely to respond to split-belt treadmill therapy;
2. Specify the appropriate training parameters for split-belt treadmill therapy in chronic stroke; and
3. Discuss key differences in split-belt treadmill walking between adults and young children, and how these differences may impact pediatric treadmill training.

**Relevance/Pertinence:** Regaining the ability to walk is often a focus of physiotherapy following a stroke or other injury to the central nervous system. Asymmetries in the spatiotemporal characteristics of gait limit walking speed (Olney et al. 1994) and hence, function. Moreover, these asymmetries can be particularly difficult to correct. The split-belt treadmill is showing great promise as a training device for hemiplegic and other asymmetric gaits. Understanding how the split-belt treadmill is being used in clinical practice and research will benefit physiotherapists and the care they provide to their patients.

**Target Population/Population cible:** This session will be relevant to physiotherapists involved with gait re- training, especially those who work with neurological populations. It will also be of interest to researchers in the areas of gait and motor learning, and to individuals who have sustained damage to the central nervous system.

**Supporting Evidence/Résumé des faits:** We use a split-belt treadmill to study how the nervous system learns and executes both temporary and long-term changes in walking. When able-bodied adults first walk on such a treadmill, their walking is asymmetric, e.g., the step lengths of the two legs are unequal (Reisman et al. 2005). These asymmetries gradually disappear after several minutes of continued split-belt walking. This process is called adaptation. When the belts are run at the same speed following split-belt walking, the walking is asymmetric in the direction opposite to that seen initially (called an aftereffect), suggesting the normal motor output has been modified (Bastian 2006).

The cerebellum is vital for this type of learning, as decerebrate cats with impaired cerebellar function (Yanagihara and Kondo 1996) and humans with cerebellar damage (Morton and Bastian 2006) do not show adaptation or aftereffects with split-belt walking. Cerebral structures, however, seem less essential for adaptation. Decerebrate cats (Yanagihara et al. 1993) and individuals with stroke (Reisman et al. 2007) can successfully adapt their walking pattern after a single session of split-belt walking. Likewise, children who have undergone a hemispherectomy retain the ability to adapt the spatial characteristics of their gait (e.g., step lengths) during split-belt walking, but show reduced adaptation of temporal characteristics (e.g., double support time) (Choi et al. 2009). Taken together, these findings suggest that an intact cerebellum, but not cerebrum, is required to learn new gait patterns on a split-belt treadmill.

Since individuals with cerebral stroke show motor adaptation, the split-belt treadmill may be a useful therapeutic tool for this group. Many individuals with stroke walk with asymmetries in spatial or temporal parameters. A single, 15-minute session of split-belt walking can temporarily correct these asymmetries as long as the individual’s asymmetry (i.e., error) is augmented during training, rather than reduced (Reisman et al. 2007). Furthermore, individuals with stroke show transfer of the newly learned gait pattern (i.e., aftereffect) from the split-belt treadmill to over-ground walking (Reisman et al. 2009). Thus, a single session of split-belt walking can result in temporary gait symmetry during over-ground walking.

One of our goals as therapists is to achieve permanent, rather than temporary, gait symmetry. Can repetitive training on the split-belt treadmill lead to long-term changes in walking? Recent work in chronic stroke shows that repeated split-belt walking (i.e., 12 one-hour sessions spaced over 4 weeks) results in a more symmetric gait during over-ground walking for up to three months post-training (Reisman et al. 2010, Reisman et al. in submitted). We are currently studying how other walking parameters, such as metabolic cost, change with long-term split-belt training, as well as comparing the outcomes of split-belt treadmill therapy with that of traditional treadmill training.

Asymmetric gait patterns are seen in pediatric populations as well; for example, in children with hemiplegic cerebral palsy or following hemispherectomy (Choi et al. 2009). We have found that young children (i.e., <5 years of age) can adapt the temporal characteristics of walking, but have difficulty with spatial adaptation (Musselman et al. 2011, Vasudevan et al. 2011). This finding is not surprising given that the cerebellum, which is vital for motor adaptation, continues to develop over the first few years of life (Altman and Bayer 1997, Lavezzi et al. 2006). Compared with adults, children show greater transfer of the newly learned pattern from the treadmill to over-ground walking (Torres-Oviedo et al., in preparation). This may suggest that split-belt walking has greater therapeutic potential in children. Yet, young children show less retention of the new gait pattern acquired during a single-session of split-belt walking (Musselman and Bastian, in preparation). Thus, the therapeutic benefits may take longer (i.e., require more repetitions or exposures) to be realized in children than adults.

**Session Format/formule de la session:** The session will involve a lecture component, a small-group activity and whole-group discussion. A case example will be provided to reinforce the concepts covered in the lecture.

**Conclusions & implications:** The split-belt treadmill is a relatively new therapeutic device that is being used by both researchers and clinicians to study and re-train walking after a cerebral injury. Through error augmentation, split-belt walking can correct asymmetries in adult hemiplegic gait, leading to safer gait patterns and greater independence in mobility. Through the split-belt treadmill paradigm we are learning that children are not little adults; their motor adaptation and learning are unique in several ways. Thus, they may not respond optimally to the training approaches designed for adults.

**Keywords/Mots clés:** split-belt treadmill, walking, stroke, pediatrics

# Abstract: P019 – Spasticity and movement: what’s new?

**Published:** 2013-05

**Authors:** A Mullick, A Blanchette, K Moïn-Darbari, MF Levin

**Affiliations:** * School of Physical and Occupational Therapy, McGill University, 3654 Promenade Sir William Osler, Montreal, QC; † Centre for Interdisciplinary Research in Rehabilitation, Jewish Rehabilitation Hospital, 3205, Place Alton-Goldbloom, Laval, QC; ‡ Department of Rehabilitation, Faculty of Medicine, Universite Laval, 1050 ave de la Medecine, Quebec City, QC; § Neuroscience Program, Faculty of Science, McGill University, 853 Sherbrooke Street West, Montreal, QC

**Overview & Objectives/Résumé & objectifs:** At the end of the session the participants will: 1) Be aware of the current controversies surrounding the measurement of spasticity; 2) Understand the principles underlying the new clinical measure and its application; 3) Discuss the relationship between spasticity and disordered movement patterns.

In this session, we will discuss the problems that surround the currently available clinical tools used for the measurement of spasticity and their impact on the evaluation and management of spasticity. We will then introduce a new clinical measure of spasticity based on the principles of motor control that overcomes, to a large extent, the limitations of the commonly used methods. Finally, we will discuss the relationship between spasticity and disordered movement patterns during functional task performance. Results of relevant research studies will be presented and discussed.

**Relevance/Pertinence:** Spasticity is an impairment commonly encountered in neurological physical therapy practice that does not have a universally accepted measurement tool. An objective, physiological measure of spasticity that is sensitive to identifying subtle changes, such as the angular measure of the threshold of spasticity, will help to improve the ability of clinicians to determine the effectiveness of treatment. In addition, as the threshold of spasticity is inextricably related to disordered motor control, understanding of this measure will help therapists gain greater insight of the impairment and its functional implications. Therapists will be able to understand how spasticity is related to weakness, postural instability, and abnormal activation of individual muscles in specific joint ranges with a level of precision not presently available in the clinical setting.

**Target Population/Population cible:** This session will be of interest to clinicians, educators and researchers interested in neurological populations with spasticity.

**Supporting Evidence/Résumé des faits:** A number of systematic reviews have questioned the validity of evaluation tools traditionally used to measure spasticity in the clinical setting (Malhotra et al. 2009, Sorensen et al. 2006). As an example, the most widely used clinical measure; the Modified Ashworth Scale (Bohannon and Smith 1987) measures the total muscular resistance to passive movement rather than stretch reflex excitability per se.

Although the presence or absence of spasticity may be identified using current clinical scales, the accuracy of determination of spasticity severity and the relationship between severity level and deficits of voluntary movements remain elusive. A large number of objective measures, classified as being electrophysiological, biomechanical or functional, have been described for spasticity, however none has consistently proven superior to the others.

An alternative approach to the measurement of spasticity is the evaluation of the excitability of motoneurons (stretch reflex threshold - SRT) resulting from both descending and segmental influences (Levin and Feldman 1994). It has been well established that in healthy subjects, the regulation of stretch reflex thresholds may be a major mechanism underlying the control of posture and movement (Feldman and Levin 1995). In healthy individuals, any level of force can be produced by a muscle at any angle within the joint range. Previous studies have shown that persons with central nervous system lesions have deficits in their ability to modulate muscle activity (SRT) throughout the entire or specific part of the range. The SRT measure identifies where, in the joint range, problems in modulation of muscle activity begin (Levin et al. 2000). According to the SRT measure, a higher value indicates a higher threshold and therefore a lower level of spasticity.

Previous research has reported the validity of the SRT as a measure of spasticity (Levin and Feldman 1994, Levin et al. 2000) and showed that higher values of the SRT in elbow flexors and extensors are correlated with lower scores on the Composite Spasticity Index (less hyperactive phasic reflexes and lower resistance to stretch) and less arm motor impairment according to the Fugl-Meyer scale (Levin et al. 2000). Good test- retest reliability of SRT measurement has also been demonstrated for children with cerebral palsy (Jobin and Levin 2000). In 2009, Calota et al. implemented this approach to spasticity measurement in a portable device that can be integrated in the assessment battery for spasticity in a clinical setting. The device has moderately high intra- and inter-reliability of SRT measurements for patients with moderate to high spasticity due to stroke (Calota et al. 2009).

**Session Format/formule de la session:** Background and Rationale will first be presented in lecture format (25 minutes). Results of research studies related to this background will then be presented and discussed (25 minutes). This will be followed by an open discussion between presenters and participants (10 minutes).

**Conclusions & implications:** The SRT measure overcomes most of the limitations posed by the currently available clinical tools used to measure spasticity. As the SRT relates spasticity and deficits in voluntary movement control and is also sensitive to subtle changes in spasticity, it may guide clinical decision-making.

**Keywords/Mots clés:** Spasticity, motor control, stretch reflex, central nervous system lesion

# Abstract: A116 – Translation, cross-cultural adaptation and validation of the Knee Outcome Survey-Activities of Daily Living Scale in Canadian French

**Published:** 2013-05

**Authors:** JF Esculier, JS Roy

**Affiliations:** * Centre for Interdisciplinary Research in Rehabilitation and Social Integration, Quebec Rehabilitation Institute, 525, Boulevard Wilfrid Hamel, Quebec City, QC; † Department of Rehabilitation, Faculty of Medicine, Laval University, Pavillon Ferdinand-Vandry, 1050, avenue de la Médecine, Quebec City, QC

**Purpose & Objectives / Intention & objectifs:** To perform the translation and cross-cultural adaptation of the KOS-ADLS questionnaire to Canadian French, and to evaluate its psychometric properties.

**Relevance/Pertinence:** Clinicians working with French Canadian individuals suffering from knee pain need valid, reliable and responsive outcome measures to adequately assess symptoms and functional limitations of their clients and to evaluate the effectiveness of their interventions.

**Materials & Methods/Matériel & méthods:** In accordance with standard procedures, the original English version of the KOS-ADLS was translated and cross-culturally adapted to Canadian French. Forty individuals (36.1±8.3 yrs, 21 females) with knee disorders completed the KOS-ADLS on three occasions (baseline, 2 days, 4 weeks). Questions about usual (VAS-U) and worst pain (VAS-W) were also completed to evaluate convergent validity (baseline), while a global rating of change question was completed to evaluate reliability (2 days) and responsiveness (4 weeks) of the Canadian French KOS-ADLS.

**Analysis/Analyse:** Pearson correlations were used to determine convergent validity; intraclass correlation coefficient (ICC), standard error of measurement (SEM) and minimal detectable change (MDC) were used to measure reliability; and standardized response mean (SRM) was used to quantify responsiveness to change.

**Results/Résultats:** Moderate correlations were obtained between French Canadian KOS-ADLS and VAS-U (r=-0.559, p<0.01) and VAS-W (r=-0.507, p<0.01). Test-retest reliability was excellent (ICC=0.93; 95%CI=0.86-0.97), while SEM (3.4%; 95%CI=2.8-4.4) and MDC (9.4%) were relatively low and similar to the original version. Finally, the Canadian French KOS-ADLS proved to be highly responsive to change (SRM=1.55). **Conclusions:** The Canadian French version of KOS-ADLS presents adequate psychometric properties and can therefore be used by clinicians working with individuals with knee disorders.

**Keywords/Mots clés:** Questionnaire, Outcome measure, Validation, Canadian French, Knee pain

# Abstract: A002 – Physiotherapists in Hemodialysis Units - Facilitators of Exercise Rehabilitation Programs for Ontarians Living with End-Stage Renal Disease

**Published:** 2013-05

**Authors:** S Ma, J Lui, D Brooks, TL Parsons

**Affiliations:** * Queen’s University, School of Rehabilitation Therapy, Kingston, ON; † University of Toronto, Department of Physical Therapy, Toronto, ON

**Purpose & Objectives / Intention & objectifs:** The primary objective is to characterize the prevalence and nature of exercise rehabilitation programs in hemodialysis facilities across Ontario. Exercise can effectively improve aerobic fitness, muscle composition, and quality of life amongst persons with end-stage renal disease who require long-term hemodialysis treatments. Yet surprisingly, rehabilitation services and physiotherapists are rarely involved in the care provided to these patients.

**Relevance/Pertinence:** This study is the first in Canada to report on the prevalence of hemodialysis units with exercise programs. This is important information to bring to physiotherapists as they play a key role in the management of chronic kidney disease.

**Materials & Methods/Matériel & méthods:** We used FluidSurveys(Trademark) to create an online survey containing questions regarding each hemodialysis unit’s renal program and its exercise components. We distributed the survey with an online consent form via email to 95 dialysis facilities across Ontario as identified from the Canadian Organ Replacement Register.

**Analysis/Analyse:** Survey answers were reported as a ratio between the frequency count of each response and the total number of respondents.

**Results/Résultats:** We received responses from 58 dialysis facilities, yielding a 61% response rate. Of those, only 8 facilities offered exercise programs, which included intra-dialytic and cardiac rehabilitation programs. Patient education regarding exercise and the presence of an exercise specialist such as a physiotherapist appeared to be missing elements of the 50 non-exercise facilities. **Conclusions:** Introducing a physiotherapist to the dialysis team for safe exercise prescription and monitoring may be a key to offering more exercise options to Canadians living with end-stage renal disease.

**Keywords/Mots clés:** Hemodialysis, End-stage renal disease, Exercise rehabilitation, Physiotherapist

# Abstract: A008 – Survey of interprofessional practices of physiotherapists working in the private sector with adults with low back pain

**Published:** 2013-05

**Authors:** K Perreault, CE Dionne, M Rossignol, D Morin

**Affiliations:** * Centre for Interdisciplinary Research in Rehabilitation and Social Integration, Institut de réadaptation en déficience physique de Québec, Québec City, QC; † Université Laval, Québec City, QC; ‡ URESP, Centre de recherche FRQS du Centre hospitalier universitaire (CHU) de Québec, Québec City, QC; § Institut national d’excellence en santé et en services sociaux, Montréal, QC; ¶ Department of Epidemiology, Biostatistics and Occupational Health, McGill University, Montréal, QC; ** Vieillissement, Centre de recherche FRQS du Centre hospitalier universitaire (CHU) de Québec, Québec City, QC; †† Institut universitaire de formation et de recherche en soins, Université de Lausanne, Lausanne, CH

**Purpose & Objectives / Intention & objectifs:** The objective of this study was to describe the interprofessional practices of physiotherapists working in private settings with adults with low back pain.

**Relevance/Pertinence:** In the last decades, there have been increasing demands for all health providers, including physiotherapists, to take part in interprofessional practices in health systems everywhere, in order to improve clinical outcomes for people with complex health problems, such as low back pain. To date, very little is known of physiotherapists’ interprofessional practices, especially in the private sector, where people with low back pain often consult.

**Materials & Methods/Matériel & méthods:** This was a cross-sectional quantitative survey of randomly-selected Québec physiotherapists. Participants (n=327) answered a purpose-designed online survey

**Analysis/Analyse:** We conducted descriptive analyses of primary variables.

**Results/Résultats:** The proportion of response was 67.1 %. Participants reported most frequent interactions with other providers by written or oral messages sent via their clients (respectively daily/weekly for 55.1 % and 24.1 % of physiotherapists), face-to-face unplanned discussions (41.9 %) and faxed/mailed letters (23.2 %). Most frequent stated interactions were with other physiotherapists, family physicians, physiotherapy assistants and occupational therapists. Conversely, less frequent interactions were said to occur with acupuncturists, psychologists, certain medical specialists and chiropractors. **Conclusions:** This study provides new insights into the interprofessional practices of physiotherapists working in the private sector with adults with low back pain. Based on our results, further examination of the most appropriate means of interacting with other providers is warranted. Our findings also suggest there is room for improving physiotherapists’ interprofessional practices with other providers involved in the management of low back pain.

**Keywords/Mots clés:** Interprofessional practices, physiotherapists, survey, low back pain, private sector

# Abstract: A027 – Patient prioritization preferences among entry-level students: importance of chronic pain

**Published:** 2013-05

**Authors:** M Laliberté, DE Feldman

**Affiliations:** Programme de physiothérapie, École de réadaptation, Faculté de Médecine, Université de Montréal, QC

**Purpose & Objectives / Intention & objectifs:** The aim of this study was to investigate the preferences of entry-level physiotherapy students regarding prioritization of patients for physiotherapy services. Specifically, we wer interested in preferences regarding patient with chronic pain.

**Relevance/Pertinence:** With rising demands for physiotherapy services in an aging population with a high prevalence of chronic disease and disability, and no corresponding increase in resources, triage strategies are needed. Several factors can influence decisions related to triage of patients and management of wait lists; however, not all are equally justifiable.

**Materials & Methods/Matériel & méthods:** Participants were entry-level physiotherapy students (n= 249) who attended a professional ethics course prior to embarking on their first clinical placement. Following a group discussion, students were asked to prioritize patients (Priority 1 to 5) corresponding to fictitious patient vignettes.

**Analysis/Analyse:** Chi-square analysis revealed a statistically significant difference between the different patient scenarios with respect to their priority category (χ216= 931.0, p <0.01).

**Results/Résultats:** Physiotherapy entry-level students prioritized the patient with chronic pain and the post- operative patient. The elderly patient and the one with cognitive impairment were both given low priority. **Conclusions:** Patients with chronic pain are highly prioritized by physiotherapy students. The literature shows that chronic pain and chronic conditions in general are assigned the lowest priorities among physiotherapy department triage tools. There may be a shift in attitude towards patients with chronic pain between the theoretical (student) phase and the practical (clinician) phase. Physiotherapists may need to question their triaging paradigms and ensure that these processes are based on evidence and not on bias.

**Keywords/Mots clés:** Health care rationing, resource allocation, health priority, health resources, triage

# Abstract: A028 – Alberta Collaborative Prescribing Model: A Demonstration Project

**Published:** 2013-05

**Authors:** I Muir, D Millette, S Lee, D Cooney, D Eberhart

**Affiliations:** * Physiotherapy Alberta, College and Association, Edmonton, AB; † Alberta College of Pharmacists, Edmonton, AB

**Purpose & Objectives / Intention & objectifs:** To test the feasibility of a collaborative prescribing model between physiotherapists and pharmacists and evaluate if the model provides a positive patient experience by facilitating access to timely drug therapy.

**Relevance/Pertinence:** The model explores a new way of delivering care with potential to facilitate timely drug therapy and improve patient outcomes.

**Materials & Methods/Matériel & méthods:** Seven physiotherapists and five pharmacists participated. The physiotherapists identified patients with musculoskeletal injuries meeting the inclusion criteria then referred them to the pharmacist. The pharmacists assessed the patient to determine appropriate drug therapy. The providers communicated ongoing goals throughout the episode of care. Evaluation included a patient questionnaire administered 2–4 weeks post prescription and provider telephone interviews at the end of the pilot. The patient questionnaire examined their experience with the model the provider interviews examined the collaborative process

**Analysis/Analyse:** Questionnaire and telephone responses were analysed using descriptive statistics.

**Results/Résultats:** 32 patients participated. 97% found the model very convenient, 97% felt at ease with the model, 97% believed that the model was good for their recovery and 81% reported no safety concerns. Of those reporting safety concerns, 99% were addressed. The physiotherapists and pharmacists reported a high satisfaction (average of 4.5 out of 5). **Conclusions:** The project demonstrates the feasibility of a new collaborative model for the prescription of timely drug therapy for musculoskeletal problems. The model is highly efficient, acceptable and safe to the patient, promotes collaboration between physiotherapists and pharmacists and has potential for wider-scale implementation.

**Keywords/Mots clés:** Collaborative practice, physiotherapist, pharmacist

# Abstract: A029 – Physiotherapy reporting in program management using multi-criterion decision analysis - strengthening the role of the Physiotherapy Department

**Published:** 2013-05

**Authors:** S Brolin, K Doull

**Affiliations:** Fraser Health, Surrey, BC

**Purpose & Objectives / Intention & objectifs:** To outline the process undertaken to determine the allied health reporting model (including physiotherapy) across a large multi-site organization moving to a program management reporting structure.

**Relevance/Pertinence:** Various organizational structures exist across health care organizations in Canada. As a result, high variability in reporting relationships for physiotherapists and their leaders also exist. Numerous published reports indicate negative aspects of traditional reporting models with program management. As a result, proactive work is required to ensure both organizational effectiveness while also maintaining professional integrity during times of organizational structure change.

**Materials & Methods/Matériel & méthods:** Multi-criterion decision analysis was conducted to evaluate five different reporting models for physiotherapy (and other allied health professions) into a Program Managment organizational structure. Following this well documented model for decision-making, the reporting models were rated for their ability to meet nine key strategic imperatives of the organization and program management model.

**Analysis/Analyse:** A total score (sum of scores across all strategic imperatives) for each of the five possible reporting structures were evaluated, with and without weighting applied to different strategic imperatives.

**Results/Résultats:** The highest scoring reporting model for physiotherapy was a hybrid model. In hospitals, physiotherapists would continue to report to Departments, as this met the needs of both Programs and professional integrity. In community settings (e.g. Home Health) physiotherapists would report directly to program managers. **Conclusions:** Using an objective analysis model, physiotherapy reporting in program management can be achieved in a way that meets program needs and professional integrity, and actually strengthens the role of the department in hospital settings.

**Keywords/Mots clés:** Program managment, reporting structure

# Abstract: A031 – The solidarity cooperative: A new clinical model to increase accessibility to physiotherapy services

**Published:** 2013-05

**Authors:** N Gaudreault, C Apinis, A Masetto, M Tousignant, M Couture, J Desrosiers, P Cossette

**Affiliations:** Faculté de médecine et des sciences de la santé, Université de Sherbrooke, Sherbrooke, QC; Clinique universitaire de réadaptation de l’Estrie (CURE), Sherbrooke, QC

**Purpose & Objectives / Intention & objectifs:** Most people in Quebec do not have insurance coverage for private physiotherapy services. The objective is to present the development and implementation of a new physiotherapy clinical model, the solidarity cooperative that emerged from the association between various local forces.

**Relevance/Pertinence:** This model facilitates increased accessibility to physiotherapy services, ensures training for physiotherapy students and promotes the development of research.

**Materials & Methods/Matériel & méthods:** We used evaluative research methods to design a logic clinical model. Key informant interviews were conducted to document needs and feasibility and to build consensus for an administrative entity. An inventory of resources (human, equipment, infrastructure) was carried out and private companies were contacted to determine their interest in forming a partnership. A survey among patients without insurance coverage helped to estimate their willingness to pay (yes/no and price range) for treatment from physiotherapy trainees.

**Analysis/Analyse:** Descriptive analysis for survey data.

**Results/Résultats:** 471 patients responded to the survey, with 38% and 31% agreeing to pay $10-$20 and $20-$30 per treatment respectively. An agreement was signed between the project promotors and the University for use of the infrastructure, services and equipment. Physiotherapy trainees worked under a senior physiotherapist to provide treatment to the patients. A multi-membership or solidarity cooperative, composed of support members, user members and worker members was created. **Conclusions:** Given their associative nature, solidarity cooperatives offer a new strategy for the emergence of partnerships between various local forces. This model increases accessibility to rehabilitation by offering affordable services to patients without insurance coverage.

**Keywords/Mots clés:** Cooperative, physiotherapy services, clinical model

# Abstract: A052 – Validation of an advanced practice physiotherapy model of care in an orthopaedic outpatient clinic

**Published:** 2013-05

**Authors:** F Desmeules, P Toliopoulos, JS Roy, LJ Woodhouse, M Lacelle, M Leroux, S Girard, DE Feldman, JC Fernandes

**Affiliations:** * School of Rehabilitation, Faculty of Medicine, University of Montreal, Montreal, QC; † Maisonneuve- Rosemont Hospital Research Center, University of Montreal Affiliated Research Center, Montreal, QC; ‡ Department of Rehabilitation, Faculty of Medicine, Laval University, Quebec City, QC; § Centre for Interdisciplinary Research in Rehabilitation and Social Integration, Quebec City, QC; ¶ Department of Physical Therapy, Faculty of Rehabilitation Medicine, University of Alberta, Edmonton, AB; ** McCaig Institute for Bone and Joint Health, Calgary, AB; †† Department of Physiotherapy, Sacré-Coeur Hospital, University of Montreal, Montreal, QC; ‡‡ Centre for Interdisciplinary Research in Rehabilitation, Montreal, QC; §§ Institute of Public Health Research of the University of Montreal, Montreal, QC; ¶¶ Orthopaedics Research Laboratory, Research Center, Sacré-Coeur Hospital, University of Montreal, Montreal, QC; *** Department of Surgery, Faculty of Medicine, University of Montreal, Montreal, QC

**Purpose & Objectives / Intention & objectifs:** To assess the diagnostic agreement of an advanced practice physiotherapist (APP) compared to orthopaedic surgeons as well as to assess treatment concordance, healthcare resource use, and patient satisfaction.

**Relevance/Pertinence:** In Canada, new models of care involving APPs are being implemented. However, formal validation of the efficiency and appropriateness of these models is scarce.

**Materials & Methods/Matériel & méthods:** 120 patients presenting for an initial consult for hip or knee complaints in an outpatient orthopedic hospital clinic in Montreal, Canada, were independently assessed by an APP and by three orthopaedic surgeons. Each health care provider independently diagnosed the patients and provided triage recommendations (conservative or surgical management).

**Analysis/Analyse:** Cohen’s kappas were used to assess inter-rater agreement for diagnosis and triage. Chi- Square and Student’s t-tests were done in order to compare imaging tests ordered, conservative treatment recommendations and patient satisfaction between each type of care.

**Results/Résultats:** For diagnosis, the inter-rater agreement was very high (κ=0.86; 95%CI:0.80-0.93) and high (κ=0.77; 95%CI:0.65-0.88) for triage recommendations. Significant differences were found between the two type of health care providers with respect to the type of conservative treatment recommendations made(p<0.05). No differences were found with respect to imaging tests order(p≥0.05). Patient satisfaction was significantly higher for APP care than for the surgeons care (p<0.05). **Conclusions:** APP care was found to be similar to surgeons care in terms of diagnosis, triage and use of imaging tests, while providing higher patient satisfaction. Although differences were found for the type of conservative treatment recommendations made, these results provide evidence supporting the APP model.

**Keywords/Mots clés:** Physiotherapist, Healthcare Services Research, Musculoskeletal Diseases and Professional Autonomy

# Abstract: A053 – Physiotherapy as a triage for orthopaedic surgery: an effective strategy for reducing wait times

**Published:** 2013-05

**Authors:** C Napier, R McCormack, MA Hunt, A Brooks-Hill

**Affiliations:** * Department of Physical Therapy, Faculty of Medicine, University of British Columbia, Vancouver, BC; † Department of Orthopaedics, Faculty of Medicine, University of British Columbia, Vancouver, BC

**Purpose & Objectives / Intention & objectifs:** To investigate the effectiveness of a physiotherapist as a triage for orthopaedic surgery referrals from primary care physicians.

**Relevance/Pertinence:** Physiotherapy as a triage for patients referred to orthopaedic surgery has been studied elsewhere showing over two-thirds of referrals are inappropriate and most can be managed by a physiotherapist. Within the Canadian healthcare context, this triage model is not well studied.

**Materials & Methods/Matériel & méthods:** A prospective, observational design was undertaken among patients referred to an orthopaedic surgeon for elective shoulder and knee complaints. Patients were assessed and categorized as “Surgical,” “Non-Surgical,” or “Further Investigation” by physiotherapist and surgeon sequentially. A patient satisfaction questionnaire was administered following the visit.

**Analysis/Analyse:** Level of agreement was determined using a weighted kappa statistic with a 95% confidence interval. The surgical conversion rate was calculated to assess level of appropriate referrals.

**Results/Résultats:** Substantial inter-rater agreement was demonstrated for surgical management decisions (κw = 0.77; 95% CI, 0.60—0.94). All patients reported being “satisfied” or “very satisfied” with the overall care they received from the physiotherapist. The Surgical Conversion Rate of the patients referred by the physiotherapist to the surgeon was 0.91 versus 0.22 among the patients referred by a GP or emergency physician. Conclusions: Over three-quarters of patients referred to an orthopaedic surgeon did not need to see a surgeon, and were managed by an experienced orthopaedic physiotherapist. This model could have considerable impact on reducing orthopaedic wait times in Canada by minimizing unnecessary referrals, and would also promote timely and conservative management of non-surgical conditions by physiotherapists.

**Keywords/Mots clés:** Physical Therapy Specialty; Orthopedics; Waiting Lists; Practice Guidelines as Topic; Gatekeeping

# Abstract: A060 – “Getting Back on My Feet”’: The Patient Perspective of Inpatient Oncology Rehabilitation

**Published:** 2013-05

**Authors:** L Scott, S Hollett, K Dawson, D Dimitri, M Beallor, K Berg, S McEwen

**Affiliations:** * Department of Physical Therapy, University of Toronto, Toronto, ON; † Sunnybrook Research Institute, St. John’s Rehab Program, Toronto, ON

**Purpose & Objectives / Intention & objectifs:** To explore the experiences of patients who have completed a unique inpatient oncology rehabilitation program.

**Relevance/Pertinence:** Cancer survivorship is increasing. This has led to a growing population with significant physical and psychosocial impairments requiring rehabilitation. Research investigating oncology rehabilitation programs is limited, particularly from the patient perspective.

**Materials & Methods/Matériel & méthods:** A qualitative, descriptive approach was used. The primary data source was a one-on-one semi-structured interview with three patient participants approximately two weeks post-discharge. Additionally, to better understand the population and the inpatient rehabilitation process, a focus group was conducted with four program physiotherapists.

**Analysis/Analyse:** An inductive thematic analysis was performed. Coding was conducted by two members of the research team, with a third involved to resolve discrepancies. Codes with similar elements were grouped and initial themes were named and defined. Themes were refined by reviewing the initial codes, pertinent quotes and engaging in a reflexivity process.

**Results/Résultats:** One overarching theme, “Getting Back on My Feet”, described the patient experiences with the program. They perceived the program got them back on their feet by helping them to regain physical strength and independence in meaningful functional activities, and by helping them to accept and manage limitations. Overall reconditioning, provision of resources for successful transition to home, and the opportunity to observe the success of other patients were considered key components of the program. **Conclusions:** Participants perceived the program to be a crucial contributor to enabling participation in meaningful functional activities. Further development and evaluation of inpatient oncology rehabilitation programs is supported.

**Keywords/Mots clés:** oncology, rehabilitation, patient experiences, physical therapy

# Abstract: A066 – Simulation model for hip and knee replacement wait times in an Ontario health planning region

**Published:** 2013-05

**Authors:** BM Chesworth, B Xie, S Warner, JV Bilsen, AB Sherif, C Hamilton

**Affiliations:** * School of Physical Therapy and Department of Epidemiology and Biostatistics, Western University, London, ON; † Department of Epidemiology and Biostatistics, Western University, London, ON; ‡ South West Local Health Integration Network, London, ON; § Southwest Regional Cancer Program, London, ON; ¶ Department of Epidemiology and Biostatistics, Western University, London, ON; ** Graduate Program in Health and Rehabilitation Sciences, Western University, London, ON

**Purpose & Objectives / Intention & objectifs:** To develop a simulation model for total hip and knee replacement surgery to help decision-makers understand the impact of various queuing strategies on wait times.

**Relevance/Pertinence:** The role of physiotherapists in the decision-making process for hip and knee joint replacement surgery is expanding in Canada.

**Materials & Methods/Matériel & méthods:** We used 2011 wait time data for hip and knee replacement surgery in an Ontario regional planning area (n=3068) to represent decentralized queuing for surgery (i.e. queue within individual hospitals). Using simulation software, the following alternative regional queuing scenarios were created: a centralized waiting list for all surgical candidates, a centralized waiting list for urban hospitals and a decentralized waiting list for rural hospitals, three other scenarios with different combinations of hospitals assigned to centralized and decentralized queuing methods. No data indicating involvement of physiotherapists in surgical decision-making were available.

**Analysis/Analyse:** For each scenario we calculated the mean wait time and the benchmark wait time defined as the 90th percentile.

**Results/Résultats:** The mean/90th percentile wait times for decentralized queuing in the region were 123/177 days, respectively. All other scenarios decreased these two values. Specifically, a centralized waiting list for all surgical candidates in the region decreased these values to 93/125 days, respectively. All scenarios with some centralized queuing yielded a smaller range for wait times. **Conclusions:** Centralized waiting lists mainly reduce variation in wait times, with less impact on mean or median values. They also help prevent extremely long wait times. Decision-making tools that evaluate queuing strategies for surgery need to account for physiotherapists’ involvement in the decision-making process.

**Keywords/Mots clés:** total hip and knee replacement, waiting lists, queuing strategies, decision-making

# Abstract: A068 – A Survey of the Work-Related Quality of Life Among Rehabilitation Therapy Professionals at an Urban Rehabilitation Hospital in Canada

**Published:** 2013-05

**Authors:** E Bates, J Beatty, S Brown, T Cameron, K Berg, M Gomez, M Lung

**Affiliations:** * Department of Physical Therapy, University of Toronto, 160-500 University Avenue, Toronto, ON; † St. John’s Rehab Research Program, Sunnybrook Health Sciences Centre, 285 Cummer Avenue, Toronto, ON and Graduate Department of Rehabilitation Science, University of Toronto, 160-500 University Avenue, Toronto, ON

**Purpose & Objectives / Intention & objectifs:** The objectives of this study were to evaluate the work-related quality of life (WRQoL) of Rehabilitation Therapy Professionals (RTPs); compare WRQoL across different RTP professions, between inpatient and outpatient RTPs, and between RTPs with and without weekend shifts; and explore any additional RTP-identified WRQoL factors.

**Relevance/Pertinence:** Knowledge of RTP WRQoL can aid in policy, program, and practice development, guide initiatives to enable a healthy rehabilitation workforce, and help health care organizations promote work environments that foster job satisfaction and staff retention.

**Materials & Methods/Matériel & méthods:** An anonymous online survey was emailed to 132 RTPs (physiotherapists, occupational therapists, physiotherapist and occupational therapist assistants) at an urban rehabilitation hospital. The questionnaire collected demographics, work characteristics, responses to a WRQoL Scale, and any subjective WRQoL comments.

**Analysis/Analyse:** Descriptive statistics were provided for demographic data and WRQoL values. Kruskall- Wallis was used to determine differences among professional groups; Mann-Whitney U for differences in clinical settings and between those with and without weekend shifts. A content analysis investigated common themes in the comments.

**Results/Résultats:** Fifty RTPs completed the survey. No difference was found in WRQoL among RTP professions (p = .96). Inpatient RTPs reported a higher WRQoL than outpatient RTPs (p < .05). RTPs with weekend shifts had higher WRQoL than those without (p < .05). Relationships with management, job security, and team cohesiveness were expressed as contributing to WRQoL. **Conclusions:** RTPs working in inpatient settings with weekend shifts had higher WRQoL than RTPs in outpatient setting and without weekend shifts. Good management relationships, job security, and team cohesiveness may contribute to better WRQoL.

**Keywords/Mots clés:** Work-related quality of life; Rehabilitation therapy professionals; Physiotherapists; Occupational Therapists; Physiotherapist Assistants; Occupational Therapist Assistants.

# Abstract: A069 – Exploring Client-Centred Care Experiences in In-patient Rehabilitation Settings (Pilot)

**Published:** 2013-05

**Authors:** E Bamm, P Rosenbaum, P Stratford, S Wilkins, N Mahlberg

**Affiliations:** School of Rehabilitation Science McMaster University, Hamilton, ON

**Purpose & Objectives / Intention & objectifs:** To explore supports and barriers to the development of productive partnership between clients and clinicians from the patient and family perspectives.

**Relevance/Pertinence:** Client-Centred Care (CCC) is emerging as a best practice in health care. Partnerships between patient, family, and health professionals in planning and delivery of health services are known to improve outcomes and satisfaction with care. Studies report lack of understanding of the elements involved in creating this partnership, and client-specific needs and preferences depending on personal beliefs, disease trajectory, and assertiveness.

**Materials & Methods/Matériel & méthods:** To date, four patients and one family member who had received treatment for at least 2 weeks at one of the four Rehabilitation Centres in South-Central Ontario have participated in semi-structured interviews.

**Analysis/Analyse:** A thematic analysis of transcripts was performed using the Grounded Theory approach developed by Strauss and Corbin.

**Results/Résultats:** Several major themes were extracted: 1) Compliance- the majority of clients assumed a passive position, accepting treatment with no interest in voicing their opinion; 2) family involvement in care and decision making improved both patients’ and families’ experiences of rehabilitation; 3) more information was needed about the rehabilitation progression and alternative treatment options; 4) personal attributes of clinicians (caring, positive attitude, helpfulness) were important in having a positive experience. **Conclusions:** The results of the study suggest that we need to encourage and educate clients to become motivated, well-informed, proactive participants in their care. In addition, clinicians have to create supportive and positive environment to improve health care experience of rehabilitation, and provide timely and comprehensive information.

**Keywords/Mots clés:** Client-Centred Care, partnerships in care, rehabilitation

# Abstract: A070 – Engaging patients and families to develop safety indicators for rehabilitation

**Published:** 2013-05

**Authors:** G Tardif, F Fancott, A Andreoli, M Lowe, S Sharpe, F Schwartz, J MacNeil

**Purpose & Objectives / Intention & objectifs:** This project aimed to engage patients, families, leaders and clinicians in the development of safety indicators relevant for rehabilitation settings.

**Relevance/Pertinence:** Most hospitals measure safety in terms of adverse events such as falls, infection rates and pressure ulcers. While important, this approach offers a limited view of safety in rehabilitation, and does not necessarily reflect the goals and priorities important to key stakeholders, including physiotherapists and the patients they serve, who may have broader perspectives of safety.

**Materials & Methods/Matériel & méthods:** Patients, families, leaders and clinicians (n=39) were engaged in a series of focus groups and a consensus-building q-sort process to develop and refine safety indicators for rehabilitation. From these collaborative sessions emerged nine safety priorities for rehabilitation.

**Analysis/Analyse:** The “Top 9 indicators” focus on areas related to communication, care transitions, coordination of care, staffing levels and the physical environment. Data for these indicators were collected using three validated tools that examined patient experience and satisfaction, care transitions and staff perceptions of safety culture over a 12-month period. From this pilot, a new “safety scorecard” for a rehabilitation was developed.

**Results/Résultats:** The indicators developed by the key stakeholders represent aspects of safety that are relevant and meaningful within rehabilitation settings, and provide a measurable means in which to target areas for improvement in quality and safety. **Conclusions:** This project represents a new level of engagement of patients and families at the organizational level to co-design systems and processes of care, together with clinicians and leaders, that strives to improve the safety and quality of rehabilitative care.

**Keywords/Mots clés:** Patient and family engagement, rehabilitation, safety indicators

# Abstract: A075 – Collaborative care: Applying change in a Canadian hospital

**Published:** 2013-05

**Purpose & Objectives / Intention & objectifs:** To apply change in collaborative relationships by engaging interdisciplinary individuals and groups of healthcare providers, mid-level and senior level leaders, across the hospital.

**Relevance/Pertinence:** Physiotherapists interested in building collaborative care relationships with patients, families and co-workers will learn about collaborative education and collaborative research strategies used to advance cultural change.

**Materials & Methods/Matériel & méthods:** A critical collaborative ethnography used sequential and mixed methodologies including interviews, focus groups, observational field notes and surveys, over three study phases. Healthcare employees volunteered to participate and their perspectives were examined in association with patient and family centred care education. The organization and the associated university provided research ethics clearance.

**Analysis/Analyse:** Ethnographic analysis followed the constant comparison and inductive method (LeCompte and Schensul, 1999). Frequency counts and measures of central tendency were used to analyse survey and demographic data.

**Results/Résultats:** Participants reported changed values and practices that resulted in improved collaborations. A conceptual framework of partners-in-care was created with six supporting sub-themes. Five organizational tensions were described. Over sixteen innovations were created and some were adopted to evolve the work culture toward greater collaboration. **Conclusions:** A shared conceptual framework of partners-in-care assisted the participants to make sense of the values and factors important to them with respect to collaboration. This work facilitated the development of many innovations to enhance collaborative practice within the hospital. Findings have been used for organizational development, for example, the patient centred care education was re-introduced in a new format following outcomes related to this study. Results are available for ongoing cultural change with respect to collaboration.

**Keywords/Mots clés:** critical ethnography, cultural change, organizational learning, organizational tensions, innovations, interprofessional practice

# Abstract: A081 – Thinking Beyond Our Borders: Investigating Ideal Competencies for Canadian Physiotherapists Working in Resource-Poor Countries

**Published:** 2013-05

**Purpose & Objectives / Intention & objectifs:** To explore ideal competencies for Canadian physiotherapists working in resource-poor countries, from the perspective of Canadian physiotherapists with global health experience.

**Relevance/Pertinence:** Physiotherapists from resource-rich countries working in resource-poor countries face a unique set of contexts, challenges, and shortcomings. Despite a growing number of Canadian physiotherapists working in resource-poor countries, there are currently no competencies available to facilitate meaningful engagement.

**Materials & Methods/Matériel & méthods:** We used a qualitative interpretive methodology. We employed the Essential Competency Profile for Physiotherapists in Canada, 2009 (ECP) as a basis for investigation and analysis. We conducted semi-structured, 60–90 minute, one-on-one interviews with 17 Canadian physiotherapists who have worked in resource-poor countries.

**Analysis/Analyse:** Each interview transcript was coded by two independent researchers to record recurring ideas. We organized the data into main themes to compare with existing ECP roles and identify additional competencies and novel roles that are important for physiotherapy work in resource-poor countries. We investigated relationships between recurring themes, roles, and competencies using schematic diagrams.

**Results/Résultats:** The seven ECP roles - Expert, Communicator, Collaborator, Manager, Advocate, Scholarly Practitioner, and Professional - were all viewed as important for physiotherapists working in resource-poor countries. Two roles, Communicator and Manager, had additional competencies that participants felt were important. Three novel roles, Global Health Learner, Critical Thinker, and Respectful Guest, were created to describe other competencies related to global health deemed crucial by participants. **Conclusions:** In addition to the ECP roles, supplementary competencies are recommended for meaningful physiotherapy engagement in resource-poor countries. These findings align with ideas in current global health and international development literature.

**Keywords/Mots clés:**

# Abstract: A090 – Can We Reliability Estimate Ischial Tissue Health?

**Published:** 2013-05

**Authors:** S Gabison, MC Verrier, EN Nussbaum, MR Popovic, S Mathur

**Affiliations:** Rehabilitation Engineering Lab, SCI Mobility Lab and Muscle Performance Lab, University of Toronto, Toronto; Rehabilitation Institute, Lyndhurst Centre, 520 Sutherland Drive, Toronto, ON

**Purpose & Objectives / Intention & objectifs:** To evaluate the reliability of quantification of ischial tissue health in individuals using ultrasound (US) imaging, colorimetry, and skin temperature.

**Relevance/Pertinence:** Changes in skeletal muscle and skin over the ischial tuberosity (IT) have been used as predictors of pressure ulcers in spinal cord injury (SCI). However to date, standardized assessment methods have not been developed nor have studies established standardized estimates of tissue health. Furthermore, the reliability of US imaging, colorimetry, and skin temperature has not been established.

**Materials & Methods/Matériel & méthods:** Four healthy males and six healthy females, mean age 42.8 years participated in the study. US scanning, skin temperature, skin redness, and sensation were collected over the IT of the dominant limb in side lying, pre and post one hour of sitting. Thickness and percentage of skin, muscle, and subcutaneous tissue from US images were calculated using OSIRIX Software. Estimates of skin redness were collected using Spectrophotometer. Minimal Detection Threshold was estimated using Semmes Weinstein Monofilaments.

**Analysis/Analyse:** Descriptive statistics and intraclass correlation (ICC) coefficients with confidence intervals were calculated for US, skin temperature and colorimetry measures. ICC Kappa values were calculated for sensory testing.

**Results/Résultats:** Skin thickness, muscle thickness and percentage of subcutaneous tissue can be reliability quantified in healthy individuals (ICC=0.88-0.94). Skin redness and skin temperature change following one hour of continuous sitting. **Conclusions:** While methodological challenges exist in establishing tissue health measures, reliability of quantifying tissue health is high, and can be used for comparisons with SCI populations who are at risk of developing pressure ulcers.

**Keywords/Mots clés:** ultrasound imaging, ischial tuberosity, spinal cord injury, pressure ulcer

# Abstract: A105 – Parenting needs of parents with disabilities for their school-age children

**Published:** 2013-05

**Authors:** R West, V Thelwell-Denton, R Wightman, S Loi, K Yoshida, N Barry

**Affiliations:** * The University of Toronto, Department of Physiotherapy, 500 University, Toronto, ON; † University of Toronto, Department of Physiotherapy, 500 University, Toronto, ON; ‡ Carpenter S; Centre for Independent Living, 365 Bloor Street East, Suite 902, Toronto, ON

**Purpose & Objectives / Intention & objectifs:** The available literature on parents with disabilities focuses primarily on parenting needs for their infants and pre-schoolers. As a result, the purpose of this qualitative study was to examine the parenting needs of parents with disabilities for their school-aged children (Grades 1–12).

**Relevance/Pertinence:** Physiotherapy student curriculum should emphasize the disability perspective to increase awareness of parenting roles of persons with disabilities. Physiotherapists should be educated to raise awareness regarding support services and assisting parents with disabilities to access those supports

**Materials & Methods/Matériel & méthods:** We conducted face-to-face interviews with eight parents with various disabilities, living in the Greater Toronto Area who had or currently have school-aged children. Based on interview response, we created a coding system and identified common themes using a constant comparison approach to data analysis.

**Analysis/Analyse:** Accessibility and support were identified as the most essential components of participants’ parenting needs. Negative societal perceptions of parents with disabilities and lack of access to support services were key contributing factors prompting participants to advocate for their parenting needs.

**Results/Résultats:** Participants identified four integral aspects of their parenting needs. These were: 1. Accessibility: ease of use regarding transportation, school, communication and information and resources; 2. Formal support: assistance provided through community service organizations; 3. Informal support: assistance provided by partners, family and friends 4. Advocacy for support services. **Conclusions:** This study can assist physiotherapists and other health care professionals to enhance their advocacy roles. This includes influencing policy changes and facilitating increased access to specialized support and parenting services.

**Keywords/Mots clés:** Parents with disabilities, parenting needs, disability perspective, school-age children, accessibility, support services

# Abstract: A112 – Un guide pour la pratique physiothérapique de fin de semaine en soins aigus par une nouvelle utilisation de la grille POP-DST (postoperative physiotherapy discharge scoring tool)

**Published:** 2013-05

**Authors:** B Guérin, S Picard

**Affiliations:** Institut universitaire de cardiologie et de pneumologie de Québec, Service de réadaptation, Québec, QC

**Purpose & Objectives / Intention & objectifs:** Augmenter le temps individuel d’intervention en physiothérapie respiratoire pour la clientèle postopératoire sans ajout de ressources.

**Relevance/Pertinence:** Selon une enquête canadienne, le nombre moyen d’usagers vus par jour de fin de semaine par physiothérapeute est de 11,7. Avant le présent projet, le ratio dans notre milieu pouvait atteindre 15 usagers. Notre temps de traitement devenait sous-optimal.

**Materials & Methods/Matériel & méthods:** Nous avons répertorié 97 usagers en phase postopératoire sur une période de 10 semaines. La grille d’évaluation et l’impression clinique du physiothérapeute nous ont permis d’objectiver l’évolution des usagers. De plus, nous notions si le plan infirmier seul aurait suffi à bien répondre aux besoins de l’usager.

**Analysis/Analyse:** Nous avons utilisé une méthodologie mixte.

**Results/Résultats:** En appliquant une nouvelle valeur seuil avec la grille d’évaluation, 97.9% des usagers ayant des besoins en physiothérapie respiratoire ont été ciblés. Nous avons mis en place une procédure simple permettant de récupérer les usagers non détectés avec la grille. Notre temps moyen de traitement est passé de 20 à 40 minutes.De 2009 à 2011, plus de 300 heures travaillées durant la fin de semaine ont été déplacées sur semaine tout en améliorant la disponibilité des services. **Conclusions:** Depuis février 2010 la cote de 9 est devenue notre nouvelle valeur seuil à la grille d’évaluation. Les physiothérapeutes juniors sont plus confiants dans leur prise de décision. Ce changement de pratique est très accessible et ne nécessite aucun ajout de ressources ou formation longue ou complexe. Nous sommes en attente d’une reconnaissance de pratique exemplaire par Agrément Canada.

**Keywords/Mots clés:**

# Abstract: P002 – Influencing health policy by telling our ’story’ - questioning current models in health human resource forcasting from the perspective of a decision-maker

**Published:** 2013-05

**Authors:** S Brolin

**Affiliations:** Fraser Health, Surrey, BC

**Overview & Objectives/Résumé & objectifs:**

1. To review current models of health human resource planning in physiotherapy
2. To discuss current factors that influence decision/health-policy makers
3. To propose a prospective model for health human resource planning in physiotherapy
4. Call to action for the profession

**Relevance/Pertinence:** With both a growing and aging population in Canada, the need for physiotherapy and its role in promoting health among our citizens is well established in the literature. Given these impending changes within our population, the need to forecast the numbers of physiotherapists required to meet the public need is imminent. Forecasting is a high stakes process both for health decision/policy makers due to human resource cost, physiotherapists who work in a strained system or who may go to great effort to navigate across a global health system and to the public who ultimately fund and depend on our health system.

Most published literature related to health human resource planning for physiotherapy make attempts to craft predictive models (with varying levels of detail) from a retrospective lens of utilization. Very few models have been identified that look at prospective value of the profession, and those that do utilize data elements that are not readily available. The purpose of this proposal is to engage leaders in the profession in discussion about demonstrating physiotherapy value, in clear and certain terms, that is prospective in nature and can easily be translated into ’stories’ that are likely to influence decision-makers.

**Target Population/Population cible:** Physiotherapy managers, practice leaders, supervisors or anyone that aims to plan for the physiotherapy resources needed for their organization and to influence decision-makers by telling the ’story’ of physiotherapy value.

**Supporting Evidence/Résumé des faits:** A review of current literature in health human resource planning for physiotherapy will be presented (systematic reviews, published reports on professional profile, case reviews, health policy documents, etc) that outline current models for health resource forecasting, over the past ten years.

Evidence on the factors that most influence decision making will be presented based on large longitudinal behavioural studies among health policy makers. Focusing on the hierarchical influences of data, stories and relationships, this presentation will focus on the draw-backs of current forecasting models in influencing decision/policy-making related to physiotherapy forecasting.

To support a new model of Physiotherapy resource forecasting, examples of successful strategies implemented in a large BC health authority will be shared. Pilot data demonstrating the impact of a prospective, outcomes-based model of utilization prediction will also be shared to demonstrate the impact of this construct.

**Session Format/formule de la session:** A review of prevalent health human resource models will be presented (comprising of most relevant, recent literature and health policy), with emphasis on the value of retrospective lens that these models provide. A concurrent look at the few contemporary models that look at prospective forecasting will be reviewed, and the subsequent limiations that make implementation difficult.

Before moving to a proposed model of forecasting a review of behavioural studies that outline the factors that influence decision making in health policy will be reviewed and then validated in discussion among session participants.

A new construct for resource planning in physiotherapy will be presented that is prospective in nature, keeps the outcome of the patient at the centre and focuses on telling the physiotherapy value ’story’ will then be proposed.

Sufficient time will be reserved following this presentation for open dialogue and engagement among session participants.

**Conclusions & implications:** This session hopes to bring awareness to patient-centred, outcome-focused prospective planning for physiotherapy. Focusing on the factors that influence decision-makers as the basis for planning, rather than retrospective data analysis on limited metrics will conclude in a call to action to change how we tell the physiotherapy value story and to shift the research agendas that inform this story.

**Keywords/Mots clés:** Health human resources, Health policy

# Abstract: P010 – Treating people with cancer: Opportunities for Physiotherapists exist, but what are the learning needs?

**Published:** 2013-05

**Authors:** O Cheifetz, A Smart, Dorsay J Park

**Affiliations:** * Clinical Specialist, Oncology division

**Overview & Objectives/Résumé & objectifs:** Purpose To review and explore opportunities for physiotherapists in oncology rehabilitation and share methods for establishing self-proficiency in the management of people with cancer.

At the end of the session participants will be able to:

1. Discuss the recent evidence supporting the use of exercise with people diagnosed with cancer.
2. Reflect and assess their knowledge and skills prior to working with people with cancer.
3. Plan strategies and identify resources they can use to meet identified learning needs related to working with this growing, complex population.

Literature suggests that exercise helps improve cancer treatment outcomes (e.g. reduces fatigue and emotional distress), improves cancer survival, prevents decline in physical fitness and functional abilities, and reduces the risk of recurrence of certain cancers. The presenters will review the latest evidence and relate the evidence to physiotherapy practice. Case presentations will assist participants to reflect on their current knowledge and skills with respect to the client with cancer, allowing them to identify potential areas for new learning. Strategies to facilitate the development of learning plans to address these needs will be discussed.

**Relevance/Pertinence:** People with cancer present with complex medical, physical, and functional challenges that require the intervention of competent health care provider. Cancer survivor safety is the cornerstone of cancer rehabilitation. Physiotherapists looking to expand their practice to include oncology rehabilitation need a thorough understanding of the impact of cancer and its treatments (Cristan et al, 2012). As self- directed learners, Physiotherapists need to know how to evaluate their knowledge and reflect on their required learning to ensure safe care of their oncology patients. Furthermore, as the field of cancer rehabilitation expands, it is imperative that Physiotherapists are well positioned to be identified as experts in the care of these patients and be viewed as integral team members of the cancer care team.

**Target Population/Population cible:** Physiotherapists and physiotherapist assistants interested in working with people diagnosed with cancer or those who find their clinical practice is changing to include more people with cancer; educators; leaders/managers interested in developing cancer rehabilitation programs and require information about staff learning needs to implement a safe and evidence based service.

**Supporting Evidence/Résumé des faits:** The Canadian Cancer Society estimates 186,400 new diagnoses of cancer in 2012 (Canadian Cancer Society, 2012). The rise in cancer diagnoses is expected to be 71% between 2012 and 2031, while the rise in population is predicted as 19% (Canadian Partnership Against Cancer, 2011). The lifetime probability of being diagnosed with cancer is 40% for women, and 45% for men in Canada.

More Canadians are living with cancer due to improved survival rates thank to better screening, earlier detection, and more effective treatments. In many cases, cancer is no longer considered to be a life shortening disease, but a chronic condition. The average five year survival rate for all cancers combined is 62%. Some types of cancer have poorer survival rates (lung, pancreas), while others, such as breast cancer has an 88% five year survival rate.

While more people are surviving cancer, this survival often comes with short and long term physical impairments and disability. Cancer survivors are more likely to experience disability than those who have not had cancer. (Canadian Partnership Against Cancer, 2010).

Physiotherapists play an important role in all phases of the cancer experience. Unfortunately, the evidence for cancer rehabilitation is not well established (Silver and Gilchrest, 2011). Safe exercise practices for the patient with cancer are a critical component of cancer rehabilitation. A recently published Cochrane Review (Mishra et al., 2012) concluded that when exercise interventions are compared with control interventions, those who exercised experienced a positive impact on overall health related quality of life. Safe cancer rehabilitation should be evidence based and be provided by regulated health care professionals such as physiotherapists (Cristan et al, 2012).

Unfortunately, one of the barriers to physiotherapists providing care to people with cancer is the lack of training in this specialized population (Silver and Gilchrest, 2011). As a result, few cancer survivors have access to rehabilitation professionals with the additional skill set and knowledge in cancer. For established physiotherapists, identifying these learning needs and seeking resources may seem overwhelming. One Australian qualitative study related to professional development plans for working with people with cancer lists Australian and International examples of resources to address learning needs (Cancer Australia, 2011). A similar resource has not yet been found for Canada.

**Session Format/formule de la session:** This session will be a lecture format, with opportunity for participant discussion regarding their expertise and reflection on their practice and self-directed learning methods

**Conclusions & implications:** While evidence is showing the potential impact of exercise on reducing the risk of recurrence for several types of cancers, the burden of disease and costs to the health care system remain. The role of Physiotherapists in cancer rehabilitation is evolving and the need for expertise in this area is likely to increase. Attendance at this session will assist physiotherapists to identify potential learning needs with respect to working with people with cancer and the resources available to address those needs.

**Keywords/Mots clés:** Exercise, Cancer, Rehabilitation, Education

# Abstract: P012 – Motor Control Based Therapy

**Published:** 2013-05

**Authors:** MF Levin, M Robert, M Rodriguez

**Affiliations:** School of Physical and Occupational Therapy and Department of Neuroscience, McGill University and Centre for Interdisciplinary Research in Rehabilitation, Jewish Rehabilitation Hospital, Laval, QC

**Overview & Objectives/Résumé & objectifs:** At the end of the session the participants will: 1) be able to identify major concepts in motor control; 2) understand how these concepts can explain some of the motor deficits in neurological and musculoskeletal patients; 3) describe how these concepts can be used in training programs to enhance motor recovery.

**Relevance/Pertinence:** Physiotherapists working with people who have mobility problems are interested in maximizing the recovery of movement. Devising treatments to recover or re-educate movement requires an understanding about how movement is controlled in the healthy nervous system and how motor control is affected by CNS or musculoskeletal injury.

**Target Population/Population cible:** This session will be of interest to clinicians, educators and researchers interested in motor recovery in neurological populations. The content is also applicable to patients with musculoskeletal problems.

**Supporting Evidence/Résumé des faits:** Several theories have been proposed to explain how the CNS controls movement. The trajectory and force control model assumes that the brain is directly involved in activating specific muscles and thus in generating muscle forces each time a movement is made (Kawato 1999). However, recent evidence shows that the corticospinal and other descending systems are not involved in specifying motor commands to muscles (Raptis et al. 2010; Sangini et al. 2011; Ilmane et al. 2012). The dynamical systems approach describes how the body interacts with the environment and explains this interaction in terms of physical principles, while placing less emphasis on physiological justifications of this approach (Kugler and Turvey 1987). The ecological psychology approach assumes that movements are guided by the perception of and the specific characteristics (affordances) of objects in the environment (Gibson 1966). The threshold control theory suggests that the CNS specifies referent positions that define where in space (spatial frame of reference) neuromuscular elements should work. According to this theory, movement is produced by shifting these referent positions in space (Feldman 2011). Regardless of the theoretical approach, an important advantage of the motor control system is that for any given movement it can recruit different muscles and joints, a phenomenon called motor redundancy (Lashley 1951; Bernstein 1967). Redundancy allows the system to find many different solutions or combinations of joint rotations to perform any given task, leading to a desirable amount of flexibility. Neurological or musculoskeletal injury often results in a decrease in flexibility, limiting the number of ways a movement can be performed. Because of this limitation, the system seeks to find alternative solutions to movement such as compensations. Understanding how compensations arise will help physiotherapists devise treatments to diminish undesirable movements and increase the movement repertoire in patients recovering from injury.

**Session Format/formule de la session:** The session will consist of a 50 minute presentation of the three content areas followed by a 10 minute discussion period.

**Conclusions & implications:** Physiotherapists are the primary users of knowledge about how the central nervous system controls movement. This session will provide therapists, educators and researchers with key knowledge about motor control that will inform them about a wide range of physiotherapeutic interventions aimed at recovery or remediation of disordered movement.

**Keywords/Mots clés:** motor control, neurological practice, orthopedic practice, models of practice

# Abstract: P013 – Are physical therapists being left behind? How practice innovation and Improvement science are influencing our colleagues and why we should consider joining them

**Published:** 2013-05

**Authors:** KM Stevenson

**Affiliations:** The Groundwork Strategy, 102-220 20th Street W, Saskatoon, SK

**Overview & Objectives/Résumé & objectifs:** At the end of this session, participants will be able to:

1. Discuss approaches to quality improvement in healthcare, including Lean and the Model for Improvement
2. Provide examples of how health professionals and organizations worldwide have applied quality improvement strategies in practice; and 3) Evaluate the relevance and applicability of different improvement strategies to physiotherapy practice.

**Relevance/Pertinence:** Relative to other professions, physiotherapy in Canada has been somewhat slow to focus on the essential competencies relevant to system improvement activities. Improvement science is being rapidly taken up by other professions (the Canadian Medical Association offers professional development focused on the topic) and by health systems (in Saskatchewan, there is a commitment to system-wide implementation of Lean). As well, improvement methods often targeted at physicians, such as Improved or Advanced Access strategies to reduce wait times, are incredibly pertinent to both private practice and outpatient physiotherapy. It is critical for physiotherapists to understand the models and frameworks, the language, and the theories of improvement science in order to effectively engage in, and even lead, such initiatives in their own systems.

**Target Population/Population cible:** This session will appeal to a broad range of professionals including clinicians, managers, professional leaders, educators, and researchers. It will be of particular interest to clinicians working in systems that are currently engaged in improvement activities, including Lean.￼

**Supporting Evidence/Résumé des faits:** Faced with the evidence that it takes an average of 17 years to implement clinical trial evidence into practice, health systems worldwide have increasingly embraced the science of improvement. As a community, we are gradually adopting the idea that the professional’s role involves both doing and improving their work. Simply look at the average healthcare job description or competency profile today and you will most certainly see reference to “participating in improvement activities’. There is a gap, however, between the idea that we should do this work and providing professional development specific to being successful at this work. Improvement requires more than effort or heart, it requires methodology. Improvement science, grounded in the work of W. Edwards Deming, involves frontline staff being able to continuously improve their work processes by understanding: variation, systems, organizational and individual psychology, and the process of learning. Using these methods, healthcare organizations worldwide have seen success in improving access, safety, efficiency, effectiveness, equity, and patient-centered care. Many systems are using Plan-Do-Study-Act cycles, a tool for small and rapid cycles of change, in some form or another - the Model for Improvement is very popular, but Lean is gaining more and more focus as an approach that promises both efficiency gains and better patient care.

**Session Format/formule de la session:** The session will feature lecture, reflective exercises, and small group discussion.

**Conclusions & implications:** It is not enough to want to improve or simply to ’try harder’ in the development and implementation of best practice. True and meaningful improvements in physiotherapy practice require knowledge and use of the methods of improvement science. Focus on such competencies will not only result in improved physiotherapy practice, but will also give us the tools and language to collaborate with and even lead our colleagues in interprofessional system improvement activities.

**Keywords/Mots clés:** improvement science, Lean, Model for Improvement, physiotherapy practice

# Abstract: P014 – Vestibular Therapy – options to effectively care for the dizzy and imbalanced patient

**Published:** 2013-05

**Authors:** S Sulway, J Rutka, D Pothier, W Dillon

**Affiliations:** University Health Network: Toronto General Hospital, Hertz Clinic for Meniere’s Disease and Vestibular Dysfunction, Toronto, ON

**Overview & Objectives/Résumé & objectifs:** This session will describe the need for increased capacity for effective physical therapy for vestibular patients; it will provide an overview on how to identify vestibular pathologies and describe how our clinical team was able to design and create an ambulatory Vestibular Rehabilitation program at an academic teaching hospital. Based on our wide range of outcome measures, we have shown that our program is substantially improving the lives of our patients.

Participants will learn:

1. The relevance of vestibular therapy and how to identify and diagnose the vestibular patient through focused history taking and objective examination.
2. How to design and implement an ambulatory vestibular rehabilitation program and how this could be integrated into a variety of clinical settings.
3. A program evaluation framework to measure effectiveness through validated outcome measures.

**Relevance/Pertinence:** It is estimated that 30–40% of adults over the age of 40 will have some sort of vestibular dysfunction in their lifetime (Agrawal, 2009). Dizziness and balance disorders are conditions that are often seen by physiotherapists in all types of clinical settings; they can be the primary cause for referral or can present in conjunction with other orthopaedic or neurological conditions. It is known that the vestibular system can be affected by a number of disorders or pathologies and can be unilateral or bilateral, leaving patients with dizziness, postural instability, gaze disturbances and at risk for falls. The impact of these disorders on overall function can be substantial and there is often an associated decline in quality of life. Through a focused history and physical exam, a trained physiotherapist can identify and diagnose the problem and subsequently, either provide treatments and/or make a referral for further management as required, ensuring a better managed case.

The provision of this clinical service is within the present scope of practice for physiotherapy however there appears to be a gap between the population need and the capacity of the physiotherapy profession to provide effective programs. Establishing such a program can be done with relatively minimal resources and there is growing bodies of research that can help the physiotherapist effectively assess and treat these patients using validated outcome measures.

**Target Population/Population cible:** This session is relevant primarily to clinical outpatient physiotherapists both in private and public settings. It will also benefit physiotherapists in inpatient clinical settings who encounter patients with dizziness, both as a primary reason for admission or as an associated co-morbidity.

**Supporting Evidence/Résumé des faits:** In several countries the field of vestibular therapy is more developed, and there is corresponding academic and clinical research programs that inform best practices.

In a 2011 Cochrane Review it was noted that, for individuals with unilateral vestibular loss or benign positional vertigo, there is moderate to strong evidence that vestibular rehabilitation is a safe and effective management tool based on a number of high quality randomized control trials (Hillier 2011).

The evidence is compelling that vestibular therapy is a very effective modality to treat these types of patients. It is also evident that physical therapy is an important part of a multi-disciplinary approach that is needed to effectively diagnose and treat these cases.

The program evaluation that has been implemented in our program has clearly validated the role of physiotherapy in our multidisciplinary program that also includes audiology, nursing & case management, neuro-otology, neuro-opthamology, psychiatry and clinical research. The evaluation results will be shared as part of the presentation.

**Session Format/formule de la session:** This session will be an interactive lecture and demonstration format with opportunity for participants to ask practical questions with regard to clinical presentation, program development and evaluation. Participants will be invited to offer clinical scenarios from their own practice for discussion.

Once completed participants will have increased knowledge regarding the need for vestibular therapy in the general population, an introduction to clinical presentation, the elements needed to create a vestibular therapy (training, equipment, collaboration needs, and evaluation).

**Conclusions & implications:** Dizziness and balance disorders are common in the general population - they are often a source of frustration for many clinicians and they are costly to the healthcare system. Vestibular rehabilitation is an excellent treatment for many of these patients; it is effective at improving physical symptoms, function and improving a patient’s quality of life.

We have been able to show that an effective outpatient program can be established at relatively low costs. It also taps into a market where the demand for such services is high and yet availability is low. This an opportunity for physiotherapists to learn more about the field and we hope to provide the basic information and framework to establish and evaluate a vestibular therapy program in their practice.

**Keywords/Mots clés:** vestibular, dizziness, imbalance

# Abstract: P016 – The need for equitable access to rehabilitation

**Published:** 2013-05

**Authors:** C Sulway, G Bone, E Zack

**Affiliations:** * Toronto Central LHIN, 425 Bloor Street East, Suite 201, Toronto, ON; † Spinal Cord Injury Ontario, Toronto, ON; ‡ Canadian Working Group on HIV and Rehabilitation, Toronto, ON

**Overview & Objectives/Résumé & objectifs:** This session will explore the current situation regarding access to physiotherapy services across Canada, consider recent direction and trends in public policy, and highlight the impact on population health specifically for vulnerable populations.

At the end of this session, participants will be able to:

1. Describe the challenges in access to physiotherapy and rehabilitation services, including national and select provincial levels.
2. Discuss the importance of equitable access to rehabilitation for patients and the profession; and give examples of innovative models of care that support the case for rehabilitation services in primary care and in systems undergoing transformation and change.
3. Contribute to a consultation to identify additional partnerships, collaborations and research required to promote the position of greater equitable access to rehabilitation services.

**Relevance/Pertinence:** The Canadian Physiotherapy Association has stated that all Canadians have a right to timely and reliable access to rehabilitation for treatment of illness, injury, and disease without barriers such as financial restraints or availability of the service (CPA, 2010) and it is well understood that early access and re-access to physiotherapy treatment positively affects health outcomes, physical functioning, and quality of life (eg: Pinnington 2004, NRAS 2011, APA 2005).

As health systems undergo recent change there has been a significant impact to the level of access to physiotherapy and other rehabilitation services. These changes have an impact on clients as well as the profession and need to be understood to effectively advocate for positive change and innovative models of care that integrate rehabilitation in new ways (for example primary care models and in evidence informed best practice patient pathways).

Physiotherapists need to be part of the change and well positioned to collaborate with other professions, patient advocate groups, policy makers and researchers to move this agenda forward.

**Target Population/Population cible:** This session is relevant to physiotherapists who provide care for patients with chronic and episodic conditions, health system administrators, researchers, advocates and government representatives.

**Supporting Evidence/Résumé des faits:** There are a number of policy research studies that have documented the recent changes in supply and demand of rehabilitation services, and the subsequent impact on the models of care for a variety of conditions (Landry 2008, 2011). The Canadian Working Group on HIV and Rehabilitation (CWGHR) and the Wellesley Institute, in partnership with several organizations, are embarking on a major initiative to promote discussion, awareness and, policy and program change to increase equitable access to rehabilitation services across Canada. The strategy is multi-pronged, and includes: policy advocacy, pilot projects and building awareness and support.

Physiotherapy is a key rehabilitative service for many client populations including vulnerable populations such as persons living with chronic and episodic diseases. Literature supports the role for physiotherapy for these types of conditions including HIV, multiple sclerosis, arthritis, spinal cord injury, stroke and neurological injury. The physiotherapy profession has a role to play in shaping health systems that effectively care and treat these populations. This advocacy is needed for the patients we serve.

**Session Format/formule de la session:** This session will be an interactive presentation that will include large group discussion and consultation with participants.

**Conclusions & implications:** Equitable access to physiotherapy and other rehabilitation services is an essential part of an effective, efficient, quality health care system. There is a need for research to better inform decisions and evaluate the innovative models of care that are being implemented in various provinces. A greater understanding and knowledge of the variety of issues and impact will inform positive change.

**Keywords/Mots clés:** Access, episodic disability, HIV, Spinal Cord, Multiple Sclerosis

# Abstract: D001 – Advanced Physiotherapy Practice: Evolving Roles in Musculoskeletal Physiotherapy

**Published:** 2013-05

**Authors:** F Desmeules, J Chepeha, L McLaughlin

**Affiliations:** Centre de recherche, Hopital Maisonneuve-Rosemont, Centre affilie l’Universite de Montreal, Montreal, QC; University of Alberta, Faculty of Rehabilitation Medicine, Department of Physical Therapy, Edmonton, AB; MD/ProHealth 225 Watson Ave, Oakville, ON

**Objectives/Objectifs:** Upon completion of this session, learners will be able to:

1. Understand and identify different models of advance physiotherapy practice (APP) for patients suffering form musculoskeletal disorders of the lower limb found in Canada and internationally.
2. Synthesize emerging evidence related to the benefits of APP care models.
3. Identify barriers to and facilitators of evolving APP roles in Canada.
4. Apply the role of the APP in the context of knee, hip, spine, and shoulder injuries.

**Background/Contexte:** In recent decades, the convergence of factors such as population aging, rising health care costs, and physician shortages have made health care transformation or health reform a priority in Canada and other countries. These health care system transformations include the development of new models of non-physician led teams to improve access while maintaining quality of care. Many countries now report the use of new roles assumed by physiotherapists, such as “advanced practice” or “extended scope practice” roles. These new roles generally include role enhancement and role substitution related to traditionally performed medical or controlled acts, such as: communicating a diagnosis, triaging potential surgical candidates, postoperative follow-up, ordering diagnostic tests, and prescribing/ injecting medication. The new models of care involving advanced practice physiotherapists are ultimately aimed at improving access to care, with equal or better effectiveness, while containing costs and retaining patient and other heath care provider satisfaction. In Canada, APP care implementation and specific roles vary greatly across provinces as they are closely related to provincial health care regulations and contextual differences. Differences in implementation and roles lead to a complex situation whereby the transportability of this new and emerging model of care across the country is limited. Although these new roles have been established, evidence of a systematic evaluation of these new models of care involving APPs is scarce and sometimes difficult to interpret for the clinician.

**Relevance/Pertinence:** The evaluation of APP models remains a complex issue that involves a variety of different research designs to assess structure, process and outcomes related to these new models of care. This session will give an outlook of the different APP models of care and related evidence of these new interprofessional roles for physiotherapists.

**Target Population/Population cible:** Physiotherapists and other health care professionals new to the area of APP as well as experienced clinicians looking to assimilate the latest evidence in advanced practice physiotherapy.

**Supporting Evidence/Résumé des faits:** We will present definition and different models of APP care and related evidence based on selected systematic reviews. (Desmeules et al. 2012 and McPherson et al. 2006) and will also present evidence from different models found in Canada( MacKay, et al. 2009,Aiken et al. 2008, Aiken and McColl 2008)and result from a pilot project in the Sacré-Coeur de Montréal Hospital.

Looking at the available evidence, there is a need for more methodologically sound studies to evaluate the effectiveness of emerging advanced practice/extended scope roles for physiotherapists. Despite the lack of methodological rigor, findings provide consistent, albeit low grade, evidence that for patients with musculoskeletal disorders, APP care may be as beneficial (or more so) than usual care by physicians in terms of diagnostic accuracy, treatment effectiveness, use of healthcare resources, economic costs and patient satisfaction. We we will also highlight that APP model evaluation needs to include the following outcomes: 1- medical diagnostic agreement, triaging agreement of potential surgical candidates or clinical recommendations between advanced practice physiotherapists (APPs) and physicians; 2- effectiveness and efficiency of treatment provided by APPs; 3) economic evaluations of treatments provided by APPs; 4) patients satisfaction and 5) improvement in accessibility to care.

In the context of physiotherapists working in multidisciplinary management of patients with spine pain, the development of the APP role is relatively early in its evolution as published studies are limited in this area. To determine the usefulness of advanced practice physiotherapy in a spinal population, a physiotherapist with advanced training in orthopaedics undertook a 3 month residency with a spinal surgeon. To ensure the training was effective, the physiotherapist and spinal surgeon completed a study to determine their agreement on identifying a surgical candidate. They independently assessed 2 groups of 30 patients and independently determined whether the patients were candidates for surgical treatment. The level of agreement was calculated as Kappa values using Chance Corrected Agreement. The Kappa score was 0.84.

Surgeons generally require axial imaging before scheduling consultation imposing another wait time as well as a large use of resources that may not be indicated. Many unhelpful MRI scan reports lead to unnecessary apprehension for patients and their referring doctors and frequently stimulate additional surgical consultation requests. Most spine surgeons appreciate that an expert interview and exam can identify those patients for whom axial imaging would be useful in diagnosis and treatment.

A second study was undertaken to determine whether an advanced practice physiotherapist could predict the usefulness of MRI scans in patients with back related complaints. Seventy -five patients from three separate clinical cohorts were evaluated. The physiotherapist and Orthopaedic surgeon independently predicted from the clinical interview and exam whether they believed that an MRI scan would be helpful in patient management. The level of agreement was calculated using Kappa values and found to be 0.95. Subsequently, a 2 year evaluation and review of over 1500 cases was completed. It was found that 20% of patients assessed were surgical candidates (vs 5% in the surgeon’s typical case load). Another 10% had medical/surgical issues requiring physician input illustrating the importance of responsive medical back-up. The remaining 70% were directed to resources that best suited their needs as opposed to simply being told that they were not a surgical candidate.

There appeared to be significant demand for the service as patients attended from 7 of the 14 Local Integrated Health Networks in Ontario.

Physician confidence is a critical component to the success of the program and is often identified as a barrier to interprofessional collaboration. Interestingly, the components cited as essential by the surgeon to gain this trust included the knowledge, experience and humility of the assessor. This was particularly important in managing the ‘less than straight forward’ cases. A certification program to facilitate the acquisition of the knowledge and skills required is recommended.

**Session Format/formule de la session:** This session will include the following lectures, followed by a panel discussion:

- Three Year Experience of Advanced Practice Spine Triage Program (L McLaughlin)
- The Benefits of Advanced Practice Physiotherapy For Patients With Hip and Knee Disorders (F Desmeules)
- Glenohumeral Internal Rotation Deficits in Varsity Level Overhead Athletes (J Chepeha)

**Keywords/Mots clés:** Advanced Physiotherapy Practice, Spine, Knee and Hip Arthroplasty, Shoulder Injury, Rehabilitation

# Abstract: D003 – Exploring the lived experience of internationally-educated physiotherapists working in Canada

**Published:** 2013-05

**Objectives/Objectifs:** Upon completion of this presentation, participants will:

1. Be familiar with the professional and cultural transition of internationally educated physiotherapists (IEPT) into the Canadian health care system
2. Understand the benefits, challenges and ethical dilemmas involved with IEPTs practicing in Canada
3. Be exposed to insights and experiences of IEPTs who now practice in Canada and of physiotherapists who facilitate this transition

**Background/Contexte:** This session will provide an overview of the infrastructure that facilitates IEPTs’ introduction into the Canadian health care system. Two IEPTs and a Canadian physiotherapist with bridging experience will provide insight into key element of their lived experience and reflect on the challenges and benefits of this transition. Given the shortage of peer-reviewed research concerning the experience of IEPTs, experiential learning is essential in this field until research findings are available to drive evidence based decisions. Participants will have an opportunity to network with presenters and pose questions during the session.

**Relevance/Pertinence:** Participants, who conduct research, develop policy, work in social justice or practice as physiotherapists in Canada’s multicultural community may find the first-hand narratives from the panelists insightful and relevant to their professional development goals. Many will find that the issues discussed may also be helpful in the development of their interpersonal and professional relationships that exist within this context. Those who have a more general interest concerning the role of the physiotherapy profession in an international context will also find the panelists’ lived experiences informative.

**Target Population/Population cible:** This presentation will appeal to participants interested in physiotherapy practice in an international context, policy-development for foreign credential recognition, regulatory standard setting, research and those who support IEPTs throughout the registration and workplace integration continuum.

**Supporting Evidence/Résumé des faits:** Ensuring the adequate supply of health care providers is important to the health and well-being of Canadians. Demand for physiotherapy services is expected to increase due to Canada’s aging population and increasing immigrant population. Over 25% of physiotherapists practicing in Canada are over 50 years of age, with retirement imminent (Johnson & Baumal, 2011). While the number of new Canadian graduates mitigates some of the attrition, it is not sufficient to replace those who are expected to retire. The addition of IEPTs into the Canadian health care system can help narrow the gap. However, integrating into the Canadian system as an IEPT may present particular challenges. For example, many IEPTs completed professional education programs whose pedagogy and curriculum are very different than those of Canada’s physiotherapy educational programs. Cultural traditions and language that are significantly different from Canada’s further complicate the transition. Additionally, stereotyping among Canadian physiotherapists towards IEPTs may shape the experience. Bridging programs have been developed to facilitate this transition, in order to (Johnson, 2007). Cultural understanding in the context of health care practice have been explored in the literature (Etowa & McGibbon, 2009), as has the concept of cultural competence training for newcomers to Canada (Majumdar & Cuttress, 1999). To date, however, there is still limited peer-reviewed scholarship in the physiotherapy community concerning the experience of IEPTs and of Canadian physiotherapists working to help facilitate this transition, for example in bridging programs.

**Session Format/formule de la session:** Three presenters will discuss their experiences in either transitioning into Canada’s health care system as physiotherapists, or in facilitating these transitions. Sessions will be moderated and the audience will be encouraged to pose questions and to provide their own insights.

**Keywords/Mots clés:** Internationally-educated physiotherapist, global health, international health

# Abstract: D004 – Have advocacy – will travel: The intersection of the Advocate Role and global health physiotherapy

**Published:** 2013-05

**Authors:** SR Cleaver, M Fraser, A Andreoli, C Camden

**Affiliations:** Graduate Department of Rehabilitation Science, University of Toronto, Toronto, ON

**Objectives/Objectifs:** At the end of this workshop participants will be able to:

-Discuss various interpretations of the Advocate Role
-Connect elements of equity and justice to the Advocate Role and apply these to global health physiotherapy practice
-Recognize the usefulness of critical perspectives and be able to apply these perspectives to the analysis of discourses as expressed through texts and ideas

**Background/Contexte:** Leaders in the physiotherapy profession have called for increasing advocacy activities by physiotherapists (PTs) (Rothstein, 2000; CPA Election Tool Kit, 2011), but this role was not a central consideration until the 2009 release of the Essential Competency Profile for Physiotherapists in Canada (ECP) (National Physiotherapy Advisory Group, 2009). Based upon the CanMEDS framework (Frank, 2005), the ECP describes seven roles (one of which is “Advocate”). The Advocate Role calls for “physiotherapists [to] responsibly use their knowledge and expertise to promote the health and wellbeing of individual clients, communities, populations and the profession” (National Physiotherapy Advisory Group, 2009, p. 12).

Concurrent with the incorporation of the Advocate Role into the profession’s essential competencies, there has been a growth in interest in global health among PTs. This growth is evident in the establishment of the Canadian Physiotherapy Association’s International Health Division, as well as growing numbers of international educational opportunities as part of entry-level physiotherapy programs (Pechak and Thompson, 2009).

Health and access to health care are currently distributed in a highly inequitable fashion, a reality within Canada but markedly pronounced between Canada and many other countries. Accordingly, the discipline of global health is by definition concerned with issues of equity (Koplan, 2009). Such issues are typically situated in political and socio-economic spheres, within sight of clinical practice, but beyond the reach of the profession’s traditional interventions. In response to such challenges, the ethic of the physiotherapy profession has evolved to a phase of societal-interest (Purtilo, 2000) where consideration of justice is incorporated into our professional essence (Edwards, Delany, Townsend, and Swisher, 2011). Although the Advocate Role was not developed explicitly for global health considerations, it was incorporated into Canada’s ECP during a period of important growth for the profession’s involvement in global health.

Despite this increased interest in global health and recent emphasis on the Advocate Role, there has not yet been a unified discussion of how the two phenomena intersect. As stakeholders seek to understand the Advocate Role in more nuanced and innovative ways, and interest in global health continues to expand, there are new and varying understandings of each, let alone the intersection of the two. Moreover, there has yet to be a discussion about the application of the Advocate Role in the global health physiotherapy arena, a combination with innumerable possibilities as the multiple meanings of each are layered upon each the other.

Critical perspectives are analytical strategies that not commonly used in the physiotherapy profession’s research or clinical activities. These perspectives are tools that focus upon the assumptions, power relations, contradictions and interplay of macro- and micro-level forces that underpin ideas and determine their validity (Eakin, Robertson, Poland, Coburn, and Edwards, 1996). Critical perspectives can be useful to identify reasons why certain issues are discussed (while others are ignored), what claims are accepted as “taken-for- granted” and which parties stand to benefit from how a particular issue is framed. All of these elements are matters of interest in the emerging intersection between global health physiotherapy and the Advocate Role.

**Relevance/Pertinence:** There are currently few preparatory activities available for PTs embarking upon global health activities. An improved understanding of the Advocate Role and its relation to global health allows PTs to develop appropriate activities and resources to effectively prepare for involvement in this field. Furthermore, developing PTs’ capacities to use critical perspectives is useful to better understand the complexities inherent in global health activities.

**Target Population/Population cible:** This presentation is targeted towards PTs, PT assistants and PT students seeking to better understand the potential impact of physiotherapy in global health. This session will be of particular interest to members of the profession striving to address inequities through their professional involvement, be those inequities within Canada or beyond our borders.

**Supporting Evidence/Résumé des faits:** While inclusion of the Advocate Role in the ECP highlights its importance to physiotherapy practice in general, understanding advocacy within the context of global health has only been superficially explored. In a SWOT analysis conducted with stakeholders within and outside the physiotherapy profession, Alappat et al. (2007) identified advocacy as a critical opportunity for Canadian PTs involved with global health initiatives. More recently, in an interpretive qualitative study Cassidy et al. (2012) interviewed Canadian PTs about their global health experiences and related these to the ECP roles. In so doing they discovered general support for the utility of the Advocate Role, but this strategy was limited in that there was only minimal focus on each given role. While arguing for the need for increased advocacy among Nigerian PTs, Amusat (2009) reminds us the overwhelming amount of unmet need for rehabilitation services in this low-income country and the importance of advocacy despite the paucity of physiotherapy literature on the subject.

While it seems intuitive that discussions of global health and physiotherapy should include advocacy as a central tenet, there are very few discussions about the overlap and overlay of these two issues in physiotherapy circles. Grounding this discussion to the Advocate Role aligns it with the aspirations and norms of the physiotherapy profession in Canada, while the use of critical perspectives provides an analytical lens to better illuminate the factors at play. Finally, relating these issues to principles of justice and equity places this discussion within the ethic of societal-interest that is increasingly seen as embedded within the foundation of the physiotherapy profession.

**Session Format/formule de la session:** This workshop will consist of alternating short presentations and facilitated small-group discussions. The facilitators will present key background elements of the Advocate Role, provide descriptions of global health in which principles of equity and justice are more explicitly incorporated, and introduce the notion of critical perspectives. Participants will then work in small groups to identify the assumptions contained within the descriptions and discuss their uses, with a key question being, “Who benefits from each of these understandings?” Facilitators will then guide participants into linking these concepts together, before sharing the discussions in the larger group to compare the merits of the various analyses.

**Keywords/Mots clés:** Advocacy, global health, critical perspectives, equity, justice

# Abstract: D005 – The Dynamic business plan: Being strategic to optimize business decisions

**Published:** 2013-05

**Authors:** W Coombs, D Hopkins-Rosseel

**Affiliations:** * CEO, Advantage Health, Creekside Rehabilitation Corp., 4-12912 Symons Valley Rd., NW, Calgary, AB, Private Practice Consultant (UK, Canada); † Cardiorespiratory Clinical Specialist Professor, School of Rehabilitation Therapy, Faculty of Health Sciences, Queen’s University, Kingston, ON

**Objectives/Objectifs:** Upon completion of the session participants will understand the vital nature of a dynamic business plan and be able to review and revise their business plan by:

1. Developing and utilizing a ‘dashboard’ of their unique business components,
2. Identifying their key business initiatives for the year and
3. Learning the steps of an effective strategic analysis

**Background/Contexte:** The majority of clinical business owners recognize that they require a credible business plan to fund and open their business. What is poorly understood, and rarely implemented by owners, is that a business plan must be dynamic (used for all key and ongoing business decisions) and should be critically appraised annually in order to optimize business outcomes. It is suggested that if this is not routine practice then this significant oversight, in turn, leads to diminishing clinical outcomes.

A business plan’s function is to provide the users with clear direction regarding the depth, breadth and limits of the practice’s activities both in the moment and predicting into the future. Yet, as the external environment changes, the internal decisions of the business must reflect multiple influences impacting the business. These include political and economic climate changes and clinical practice evolutions and must be measured with key performance indicators. A well-understood, well-used and critically analysed business plan continually reminds the users of the importance of the many otherwise overlooked aspects of running a successful business. Avoiding reacting to negative forces while having the adaptability to recognize and seize opportunities becomes a primary result.

**Relevance/Pertinence:** The most important goal of physiotherapy, and thus physiotherapy businesses, is to meet the client’s expectations of optimal health and function. If a physiotherapy business is not financially viable, then it is not sustainable. The obvious consequence is either poor client care or business closure. In the Canadian context, over 44% of physiotherapists are currently employed in the private sector and/or opening and running private practices (CIHI, 2012) and this number is predicted to increase. It is, therefore, paramount that our profession assists our members to not only understand and develop the skills to be effective in business but also to realize the importance of these attributes and skills.

**Target Population/Population cible:** This session will be of interest to a broad range of healthcare professionals including private practice clinic owners, managers, contractors, public and private administrators, professional leaders and individuals interested in the improvement of the business of physiotherapy.

**Supporting Evidence/Résumé des faits:** The Canadian Physiotherapy Association’s ’Cost of Business’ Survey (2008) demonstrated that although the average Canadian physiotherapy business is profitable, not all are and many are underperforming with respect to benchmarks. There are many effective business tools developed to identify challenges and create solutions that have been applied across other business sectors but business research is limited in the healthcare sector (Zelman, 2003, Hopkins-Rosseel, 2007, Gerber, 2003). As the delivery of private practice Physiotherapy service expands a better understanding of the impact on cost and quality of service has yet to be studied (Landry, 2008). It is important to get practical and relevant business tools specific to the business of Physiotherapy in the hands of Physiotherapists and ensure they are being used on a regular basis and eventually best business practices can emerge.

**Session Format/formule de la session:** This workshop will integrate didactic components with active engagement of participants in dialogue and skill development as they build a business and strategic planning framework that they can use in their practices. This session will end with a question and answer period.

**Keywords/Mots clés:** strategic planning, business plan, business models, SWOT analysis, SCOPE Planning Model

# Abstract: D006 – Integrating Physiotherapist Assistants into Physiotherapy Service Delivery Models

**Published:** 2013-05

**Authors:** S Funk, D Yardley

**Affiliations:** * Private Practice Division, CPA; † Chair

**Objectives/Objectifs:** At the end of this session, participants will be able to:

1. Identify various service delivery models that incorporate physiotherapist assistants into physiotherapy practices in this changing health care environment;
2. Discuss the essential competencies required by physiotherapist assistants working under the supervision of a physiotherapist in Canada;
3. Analyze the financial costs and benefits of the physiotherapist - physiotherapy assistant service delivery model; and
4. Develop practical tools and strategies to assist private practice owners in the implementation of the physiotherapist - physiotherapy assistant service delivery model.

**Background/Contexte:** The growing demand for health and rehabilitation services in Canada, along with stabilization of health care financing, has created a need to become more innovative in health care service delivery. Internationally and within Canada, models exist whereby Physiotherapist assistants work under the supervision of Physiotherapists to provide effective, efficient and safe care plans. Physiotherapist assistants are employed in a variety of practice settings, are accountable for their own performance and responsible for delivering ‘assigned components’ of a treatment plan that occur within a recognizable competency-level.

Healthcare reform is leading to a state of flux within Private Physiotherapy Practices. Changing patterns in health and disease, economic and resource allocation, advancements in Information-Technology, and changing patient expectations and demands now requires innovation and creative strategies to improve accessibility to quality care. Private practice physiotherapists could benefit from the implementation of additional service delivery models and consideration of evolving their roles within the private practice sector. The public sector has demonstrated earlier adoption of the Physiotherapist-Physiotherapist-Assistant integrated model of care, compared to a slower and more traditional private practice industry. It is realistic to appreciate that increasing the utilization of Physiotherapist Assistants could be one possible resolution to address increasing cost of services, an aging population, and shortages of health human resources, but questions around fiscal responsibility, liability around supervision and appropriate assignment of tasks still weigh heavily in adoption practices.

Through collaboration with various key organizations and individuals, the Essential Competency Profile for Physiotherapist Assistants in Canada (the Profile) has been undergone revision. The most recent version, released in April 2012, is outcome-based, client-focused, and has a functional framework that outlines the measurable and valid competencies of a physiotherapist assistant. The Profile is a valuable resource primarily developed for the physiotherapist and physiotherapy profession that can easily be referenced for hiring purposes, performance evaluation, and human resource development.

**Relevance/Pertinence:** There is variation across Canada with regard to the education, training, regulation, title and role of physiotherapist assistants. Physiotherapists have different levels of understanding around the physiotherapist assistant role, scope of practice, educational level, and competencies. Concern exists around informed consent, misrepresentation of the physiotherapist, level of supervision and the types of assigned tasks. Most provincial regulatory bodies have developed policies and practice standards to assist PTs in understanding their role and responsibilities when working with physiotherapist assistants. Mitigation of professional practice concerns may facilitate a shift towards physiotherapist-assistant integration to aide in the evolving health care demands and needs of the population. Moreover, increasing economic strain, government transformations, and emerging roles within physiotherapy have implications on human resources for private physiotherapy practices. Staffing of physiotherapist assistants has the potential to decrease financial stresses on a private practice physiotherapist/owner and when implemented well can maintain a high standard and quality of care.

**Target Population/Population cible:** A prior level of exposure to this topic is not required to successfully follow this session. This session will be of interest to broad range of healthcare professionals including private physiotherapy owners, private and public practice physiotherapists, physiotherapist assistants, physiotherapy educators and physiotherapy administrators interested in furthering their knowledge in the physiotherapist - physiotherapy assistant service delivery model.

**Supporting Evidence/Résumé des faits:** Consensus exists around the necessity for improvements in the performance of health systems, including creative strategies for the delivery of health services. We realize more resources are needed, but we must also seek innovative ways of harnessing and focusing both the financial and the human resources that currently exist. Task shifting is a process whereby specific tasks are moved, where appropriate, to health workers with shorter training and fewer qualifications. By reorganizing the workforce in this way, task shifting can make more efficient use of existing human resources and ease bottlenecks in service delivery. Task shifting may also involve the delegation of some clearly delineated tasks to health workers who receive specific, competency-based training (WHO, 2008). A task shifting approach represents a return to the core principles of health services that are accessible, equitable and of good quality (WHO, 2008). The utilization of a Physiotherapist-Physiotherapist Assistant model of care can increase access to physiotherapy to satisfy the demands of an aging population, changing patterns of disease, and emerging roles within the profession. Despite the potential effectiveness and sustainability of this task shiftingapproach, much debate and resistance exists around the integration of the physiotherapist assistant and their scope of practice in the private practice sector. If physiotherapists were more familiar with the revised Competency Profile for Physiotherapist Assistants in Canada (NPAG, 2011), the six units of competency that are defined could support this task shifting approach.

American physical therapists and physical therapist assistants have been working together to provide physical therapy services since 1969 (APTA, 2011). Resources and examples of delivery models from the American Physical Therapy Association will be used to highlight successes and challenges in their implementation of the physiotherapist assistant role within the profession. Furthermore, the presenters will share personal private practice business experiences, in combination with guidance from the revised “Essential Competency Profile for Physiotherapist Assistants in Canada, released in April 2012 (and its predecessor in 2002)” to propose practical strategies for successful implementation of the physiotherapist- physiotherapist assistant delivery model. The Profile will be presented as a valuable tool for private practice physiotherapists/owners to guide hiring, training, and individual performance measurements. Human resources efficiency and effectiveness will be discussed based on practical experience, provincial resources and international data. With greater uptake from private practice physiotherapists more research can be conducted to demonstrate stronger evidence around the proposed benefits of physiotherapist assistant adoption. Such benefits include: improved access to care and highly skilled physiotherapists, more comprehensive treatment, fiscally responsible management, enhanced practice environment, stronger therapeutic relationship and improved patient compliance.

**Session Format/formule de la session:** This session will be in lecture format and will include a question and answer period at the end of the presentation that will provide participants with an opportunity to share their perspectives and experiences and pose questions to the session presenters.

**Keywords/Mots clés:** Physiotherapist Assistant, Physiotherapist, Physical Therapist, client centered care, service delivery model, essential competencies, competency profile, supervision, delegation, accountability, responsibility, utilization, implementation, strategies, service demand, patient expectations, economic factors, resource distribution, primary care, quality of care
